# Associations between statins and adverse events in primary prevention of cardiovascular disease: systematic review with pairwise, network, and dose-response meta-analyses

**DOI:** 10.1136/bmj.n1537

**Published:** 2021-07-15

**Authors:** Ting Cai, Lucy Abel, Oliver Langford, Genevieve Monaghan, Jeffrey K Aronson, Richard J Stevens, Sarah Lay-Flurrie, Constantinos Koshiaris, Richard J McManus, F D Richard Hobbs, James P Sheppard

**Affiliations:** 1 Nuffield Department of Primary Care Health Sciences, University of Oxford, Oxford, UK; 2 Alzheimer’s Therapeutic Research Institute, University of Southern California, Los Angeles, USA

## Abstract

**Objective:**

To assess the associations between statins and adverse events in primary prevention of cardiovascular disease and to examine how the associations vary by type and dosage of statins.

**Design:**

Systematic review and meta-analysis.

**Data sources:**

Studies were identified from previous systematic reviews and searched in Medline, Embase, and the Cochrane Central Register of Controlled Trials, up to August 2020.

**Review methods:**

Randomised controlled trials in adults without a history of cardiovascular disease that compared statins with non-statin controls or compared different types or dosages of statins were included.

**Main outcome measures:**

Primary outcomes were common adverse events: self-reported muscle symptoms, clinically confirmed muscle disorders, liver dysfunction, renal insufficiency, diabetes, and eye conditions. Secondary outcomes included myocardial infarction, stroke, and death from cardiovascular disease as measures of efficacy.

**Data synthesis:**

A pairwise meta-analysis was conducted to calculate odds ratios and 95% confidence intervals for each outcome between statins and non-statin controls, and the absolute risk difference in the number of events per 10 000 patients treated for a year was estimated. A network meta-analysis was performed to compare the adverse effects of different types of statins. An E_max_ model based meta-analysis was used to examine the dose-response relationships of the adverse effects of each statin.

**Results:**

62 trials were included, with 120 456 participants followed up for an average of 3.9 years. Statins were associated with an increased risk of self-reported muscle symptoms (21 trials, odds ratio 1.06 (95% confidence interval 1.01 to 1.13); absolute risk difference 15 (95% confidence interval 1 to 29)), liver dysfunction (21 trials, odds ratio 1.33 (1.12 to 1.58); absolute risk difference 8 (3 to 14)), renal insufficiency (eight trials, odds ratio 1.14 (1.01 to 1.28); absolute risk difference 12 (1 to 24)), and eye conditions (six trials, odds ratio 1.23 (1.04 to 1.47); absolute risk difference 14 (2 to 29)) but were not associated with clinically confirmed muscle disorders or diabetes. The increased risks did not outweigh the reduction in the risk of major cardiovascular events. Atorvastatin, lovastatin, and rosuvastatin were individually associated with some adverse events, but few significant differences were found between types of statins. An E_max_ dose-response relationship was identified for the effect of atorvastatin on liver dysfunction, but the dose-response relationships for the other statins and adverse effects were inconclusive.

**Conclusions:**

For primary prevention of cardiovascular disease, the risk of adverse events attributable to statins was low and did not outweigh their efficacy in preventing cardiovascular disease, suggesting that the benefit-to-harm balance of statins is generally favourable. Evidence to support tailoring the type or dosage of statins to account for safety concerns before starting treatment was limited.

**Systematic review registration:**

PROSPERO CRD42020169955.

## Introduction

Cardiovascular disease is a leading cause of mortality and morbidity worldwide.^1^ Statins are effective in reducing the risk of cardiovascular disease and have been recommended in clinical guidelines as frontline treatment for the prevention of cardiovascular disease.^2^ ^3^ Various adverse events have been reported in clinical use, however, including muscle problems, liver dysfunction, renal insufficiency, diabetes, and eye conditions.^4^ Previous studies have shown that uptake and persistence with statin treatment is poor and, as a result, millions of patients could be missing out on life saving treatment.^5^ ^6^ ^7^ ^8^ This underuse is partly because of concerns about potential adverse effects,^9^ and these concerns are particularly evident when statins are used for primary prevention in asymptomatic patients without a history of cardiovascular disease. In these individuals, who have a lower average risk of cardiovascular disease, the absolute benefits of statins are smaller than in a secondary prevention population with previous cardiovascular disease events, and therefore the benefit-to-harm balance of treatment might be less favourable.^10^ Nevertheless, recent guidelines have recommended wider use of statins for primary prevention, making a large population at low risk of cardiovascular disease eligible for treatment and exposed to the risks of adverse effects.^2^ ^3^ ^11^ This increased eligibility for intervention with statins has been controversial,^12^ and better understanding of the risks of adverse effects is needed to determine the trade-off between the benefits and harms of statins in a primary prevention population.

One possible solution to improve the use of statins in primary prevention is to apply a stratified treatment strategy, to target optimal treatments in patients with the best trade-off between benefits and harms.^13^ This strategy could involve tailoring the type and dosage of statins.^14^ Current recommendations on the choice of statin regimen are mainly based on their efficacy in reducing low density lipoprotein cholesterol.^2^ ^3^ Given the different pharmacological mechanisms by which different statins have beneficial and adverse effects, however, classification of the potencies of different types of statins based on their efficacy alone might not be appropriate when considering the safety of treatment.^15^ ^16^ Understanding the dose-response relationships of adverse effects could help determine the optimal therapeutic dose range of statins for primary prevention, avoiding doses that provide little extra benefit but might cause adverse effects.^17^ ^18^

Most previous systematic reviews of statins focused on efficacy or secondary prevention populations, making it difficult to determine the specific risks of adverse effects in patients without a history of cardiovascular disease.^19^ ^20^ ^21^ Reviews that examined harms in primary prevention have provided conflicting results, particularly for muscle problems, which were inconsistently defined and involved a wide range of muscle conditions with different severities.^22^ ^23^ ^24^ ^25^ In this study, we have systematically reviewed randomised controlled trials in adults without a history of cardiovascular disease, to quantify the associations between statins and adverse events, and to examine how the associations vary by type and dosage of statin. Our aim was to better inform the use of statins in primary prevention of cardiovascular disease.

## Methods

The study was conducted according to the Preferred Reporting Items for Systematic Reviews and Meta-Analyses (PRISMA).^26^ The study protocol was registered on PROSPERO, the international prospective register of systematic reviews (registration No CRD42020169955).

### Data sources and search strategies

To maximise the efficiency of the search, we first identified studies from six large scale systematic reviews of clinical trials of treatment with statins; the most comprehensive systematic review included studies published up to March 2013.^19^ ^20^ ^21^ ^22^ ^23^ ^24^ To supplement the previous systematic reviews and to identify more recent studies, we searched PubMed/Medline, Embase (Ovid), and the Cochrane Central Register of Controlled Trials (CENTRAL) for studies published from 1 January 2013 to 1 August 2020. Supplementary table 1 describes the systematic search strategies.

### Eligibility criteria and study selection

Eligible studies were randomised controlled trials in adults (>18 years) without previous cardiovascular events that compared statins with non-statin controls or compared different types or dosages of statins and reported at least one outcome of interest. Non-statin controls included placebo, usual care, and no treatment. Statin treatments were monotherapy or add-on treatment to usual care or non-drug treatments (eg, diet or exercise). Studies where 70% or more of the participants had no history of cardiovascular disease were considered eligible to avoid excluding large trials with a small proportion of patients with cardiovascular disease and to limit the loss of information about primary prevention patients. To avoid including early phase trials for mechanistic research, studies that enrolled fewer than 100 participants or lasted for less than four weeks were excluded. Supplementary table 2 provides the full eligibility criteria. Two reviewers (TC and LA) independently selected eligible studies by screening the title and abstract and assessing the full text. Discrepancies were resolved by consensus.

### Study outcomes

The primary outcomes were adverse events that were reported in clinical practice or that came from recent large trials.^27^ ^28^ ^29^ ^30^ ^31^ These adverse events included muscle problems, liver dysfunction, renal insufficiency, diabetes, and eye conditions. To resolve the inconsistent definitions of muscle problems in trials and distinguish their clinical importance, we classified muscle problems as self-reported muscle symptoms and clinically confirmed muscle disorders, and examined these two outcomes separately. Self-reported muscle symptoms included myalgia (muscle pain), muscle weakness, and other non-specified muscle discomforts reported by trial participants, without a substantial rise in serum concentration of creatine kinase. Clinically confirmed muscle disorders included a rise in serum concentration of creatine kinase to more than 10 times the upper limit of normal and diagnosed myopathy or rhabdomyolysis, as defined in the original trials.^32^ Liver dysfunction included a rise in serum concentration of aspartate transaminase or alanine transaminase to more than three times the upper limit of normal and other diagnosed liver disorders.^33^ Renal insufficiency included any decline in renal function, the presence of proteinuria, and other diagnosed renal disorders. Diabetes (type 2 diabetes) and eye conditions (cataracts and other eye related conditions) were defined as the diagnoses in the original trials. To compare the potential harms with the benefits of statins in the same population, we also extracted data on three major adverse cardiovascular events as secondary outcomes of efficacy: myocardial infarction, stroke, and death from cardiovascular disease.^34^ ^35^

### Data extraction and quality assessment

Extracted data included information on study design, characteristics of participants, allocation of interventions, and outcome measurements. For studies identified from the previous systematic reviews, relevant data were extracted by one reviewer (TC) and checked by another (GM) with a standardised extraction form. Extracted data were also compared with the reported data in the previous reviews to ensure accuracy. For studies identified in database searches, two reviewers (TC and LA) collected the data from each study in duplicate, followed by a cross check of consistency.

The risk of bias in individual studies was assessed with the Cochrane risk of bias tool.^36^ The quality of evidence for each outcome in the pairwise meta-analyses and for any significant results in the network meta-analyses was rated according to the GRADE (Grading of Recommendations Assessment, Development, and Evaluation) process.^37^ ^38^ Two reviewers (TC and LA) independently assessed the risk of bias and the quality of the evidence. Discrepancies were resolved by discussion.

### Statistical analysis

A pairwise meta-analysis was conducted to compare statins with non-statin controls for each primary and secondary outcome.^39^ Heterogeneity among individual studies was assessed with the Q test and quantified with the I^2^ statistic.^40^ We used a fixed effects model with the Mantel-Haenszel method to calculate pooled odds ratios and 95% confidence intervals when no significant heterogeneity was detected (P>0.05 for the Q test and I^2^ <50%); otherwise, we used a random effects model with the DerSimonian-Laird method.^39^ Absolute risk difference was estimated based on the calculated odds ratio and the overall event rate across the non-statin control groups for each outcome. To compare the absolute risk differences for safety and efficacy outcomes, the event rates derived from different study durations for each outcome were transformed into comparable annual incidences.^41^ Publication bias was examined by the Harbord test of the symmetry of funnel plots when at least 10 studies were involved. Sensitivity analyses, excluding small studies, were performed when publication bias was detected.^42^ The robustness of the pooled results was inspected by leave-one-out influence analysis.^43^ Further sensitivity analyses examined pooled effects with an alternative analysis model to that used in the primary analysis, and by excluding studies that included any patients with cardiovascular disease.

We performed a network meta-analysis by the frequentist method to compare the adverse effects of different types of statins and non-statin controls.^44^ Heterogeneity among individual studies and global inconsistency across different designs of treatment comparisons in the network were assessed with a generalised Q test, based on a fixed effects assumption for heterogeneity and inconsistency.^45^ A fixed effects consistency model was used to calculate the pooled odds ratio and 95% confidence interval for each pair of treatment comparisons. In sensitivity analyses, a random effects consistency model was used to further examine the pooled results. Although a global consistency assumption was adopted, potential local inconsistency between direct and indirect evidence within each treatment comparison was explored by node splitting analysis.^46^

We used a model based meta-analysis method, which fitted the dose specific effects from a network meta-analysis to an E_max_ dose-response model, to examine the dose-response relationships of the adverse effects of individual statins.^47^ ^48^ The E_max_ model describes pharmacological dose-response relationships with key parameters E_max_, the asymptotic maximum drug effect, and ED_50_, the dose that produces half of the maximum effect.^49^ Posterior means and 95% credible intervals of the model parameters were estimated with a bayesian approach.^47^

All statistical tests had a two tailed significance level of P≤0.05. Analyses were performed with meta, netmeta, and MBNMAdose packages in R (version 3.6.3).

### Patient and public involvement

This study is part of a wider project examining the benefits and harms of drugs used for the prevention of cardiovascular disease, which was developed with the help of our patient and public adviser. In designing this programme of work, we held two focus groups with 30 older adults to discuss issues related to drugs for the prevention of cardiovascular disease and adverse events, which informed the interpretation of this work. The results of this review will be disseminated to the relevant patient communities.

## Results

**Fig 1:**
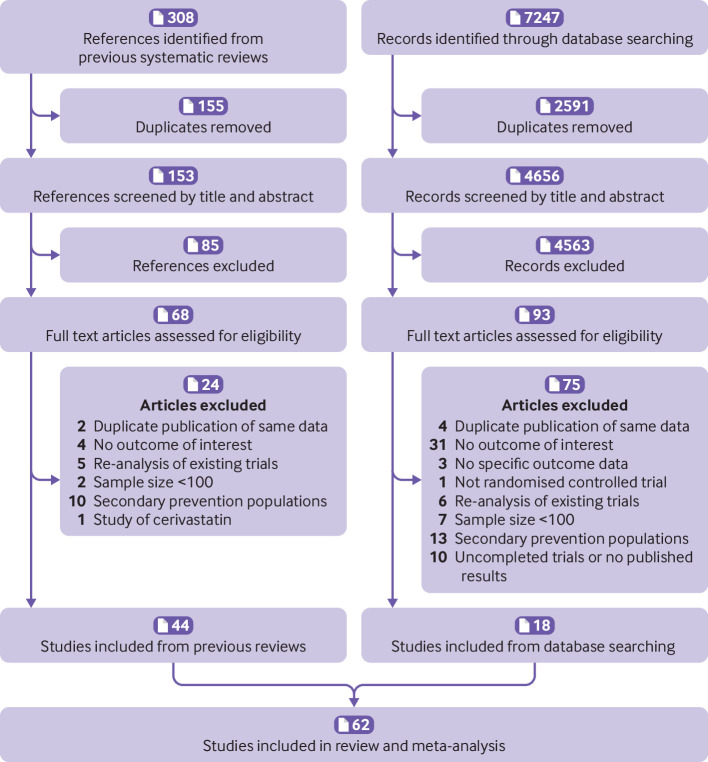
Flowchart of study selection

Our searches resulted in 7555 potentially relevant citations (308 from previous reviews and 7247 from database searches). After removing duplicates, 62 eligible studies were identified by screening of the title and abstract and assessing the full text (fig 1).^30^ ^31^ ^50^ ^51^ ^52^ ^53^ ^54^ ^55^ ^56^ ^57^ ^58^ ^59^ ^60^ ^61^ ^62^ ^63^ ^64^ ^65^ ^66^ ^67^ ^68^ ^69^ ^70^ ^71^ ^72^ ^73^ ^74^ ^75^ ^76^ ^77^ ^78^ ^79^ ^80^ ^81^ ^82^ ^83^ ^84^ ^85^ ^86^ ^87^ ^88^ ^89^ ^90^ ^91^ ^92^ ^93^ ^94^ ^95^ ^96^ ^97^ ^98^ ^99^ ^100^ ^101^ ^102^ ^103^ ^104^ ^105^ ^106^ ^107^ ^108^ ^109^ Supplementary table 3 provides a list of studies excluded after assessing the full text, with reasons for exclusion.

### Study characteristics

A total of 120 456 participants were enrolled in the included studies and followed up for a mean of 3.9 years. The mean age of all participants was 61 and 48610 (40%) participants were women. Most studies, except two, enrolled participants with hyperlipidaemia (low density lipoprotein cholesterol >3 mmol/L) or dyslipidaemia, and the common comorbidities in these participants were diabetes (11 studies), asymptomatic atherosclerosis (nine studies), and hypertension (four studies). Twenty studies included some participants with previous cardiovascular disease events, comprising 6% (7673 participants) of the total number of participants. Thirty eight studies involved a group of non-statin controls, which included placebo (35 studies), usual care (two studies), and no treatment (one study). Seven types of statins were evaluated: atorvastatin (29 studies), fluvastatin (two studies), lovastatin (five studies), pitavastatin (nine studies), pravastatin (21 studies), rosuvastatin (18 studies), and simvastatin (nine studies). The most commonly measured adverse event in these trials was clinically confirmed muscle disorders (42 studies), followed by self-reported muscle symptoms (40 studies) and liver dysfunction (38 studies). Renal insufficiency, diabetes, and eye conditions were reported in fewer studies (16, 10, and six studies, respectively). Table 1 lists the characteristics of the individual studies.

**Table 1:** Characteristics of included studies

Study, year	No of participants	Study duration	Study population			Statin treatment (dose, mg/day)	Comparator
			Comorbidities with hyperlipidaemia or dyslipidaemia	Mean age	No (%) of women		
EXCEL, 1991^50^	8245	1 year	None	56	3380 (41)	Lovastatin (20/40/80)	Placebo, different statin doses
ACAPS, 1994^51^	919	3 years	Carotid atherosclerosis	62	441 (48)	Lovastatin (20)	Placebo
PMSG-D, 1994^52^	325	4 months	Diabetes	58	159 (49)	Pravastatin (10)	Placebo
Jacobsen et al, 1995^53^	245	3 months	None	57	76 (31)	Pravastatin (20)	Placebo
KAPS, 1995^54^	447	3 years	None	57	0 (0)	Pravastatin (40)	Placebo
WOSCOPS, 1995^55^	6595	5 years	None	55	0 (0)	Pravastatin (40)	Placebo
CAIUS, 1996^56^	305	3 years	Carotid atherosclerosis	55	143 (47)	Pravastatin (40)	Placebo
Bertolini et al, 1997^57^	305	1.1 years	None	56	168 (55)	Atorvastatin (10), pravastatin (20)	Different statin types
AFCAPS/TexCAPS, 1998^58^	6605	5.2 years	None	58	991 (15)	Lovastatin (20)	Placebo
Bak et al, 1998^59^	215	6 months	None	54	0 (0)	Pravastatin (20)	Placebo
Gentile et al, 2000^60^	409	6 months	Diabetes	59	131 (32)	Atorvastatin (10), lovastatin (20), pravastatin (20), simvastatin (10)	Placebo, different statin types
ALLHAT-LLT, 2002^61^	10 355	4.8 years	Hypertension	66	5074 (49)	Pravastatin (40)	Usual care
ALERT, 2003^62^	2102	5.1 years	Renal transplantation	50	715 (34)	Fluvastatin (40)	Placebo
ASCOT-LLA, 2003^63^	10 305	3.3 years	Hypertension	63	1958 (19)	Atorvastatin (10)	Placebo
ESG-L, 2003^64^	548	3 months	None	56	318 (58)	Lovastatin (10)	Placebo
ESG-P, 2003^65^	538	3 months	None	55	301 (56)	Pravastatin (10)	Placebo
Mohler et al, 2003^66^	354	1 year	Peripheral arterial disease	68	81 (23)	Atorvastatin (10/80)	Placebo, different statin doses
STELLAR, 2003^67^	2431	1.5 months	None	58	1240 (51)	Atorvastatin (10/20/40/80), pravastatin (10/20/40), rosuvastatin (10/20/40/80), simvastatin (10/20/40/80)	Different statin types and doses
CARDS, 2004^68^	2838	3.9 years	Diabetes	62	908 (32)	Atorvastatin (10)	Placebo
DISCOVERY, 2004^69^	1024	3 months	None	61	461 (45)	Atorvastatin (10), rosuvastatin (10)	Different statin types
ESG-S, 2004^70^	1528	3 months	None	56	795 (52)	Simvastatin (10)	Placebo
Muldoon et al, 2004^71^	308	6 months	None	54	160 (52)	Simvastatin (10/40)	Placebo, different statin doses
PHYLLIS, 2004^72^	508	2.6 years	Hypertension, carotid atherosclerosis	58	305 (60)	Pravastatin (40)	Placebo
PREVEND-IT, 2004^73^	864	3.8 years	Microalbuminuria	51	302 (35)	Pravastatin (40)	Placebo
BELLES, 2005^74^	614	1 year	None	64	614 (100)	Atorvastatin (80), pravastatin (40)	Different statin types
COMETS, 2005^75^	396	1.5 months	Metabolic syndrome	58	143 (36)	Atorvastatin (10), rosuvastatin (10)	Placebo, different statin types
CORALL, 2005^76^	263	4.5 months	Diabetes	60	142 (54)	Atorvastatin (20), rosuvastatin (10)	Different statin types
HYRIM, 2005^77^	568	4 years	Hypertension	57	0 (0)	Fluvastatin (40)	Placebo
URANUS, 2005^78^	469	4 months	Diabetes	64	202 (43)	Atorvastatin (10), rosuvastatin (10)	Different statin types
ARIES, 2006^79^	774	1.5 months	None	55	503 (65)	Atorvastatin (10/20), rosuvastatin (10/20)	Different statin types and doses
ASPEN-Primary, 2006^80^	1905	4 years	Diabetes	61	724 (38)	Atorvastatin (10)	Placebo
ATOROS, 2006^81^	120	6 months	None	53	53 (44)	Atorvastatin (20), rosuvastatin (10)	Different statin types
MEGA, 2006^82^	7832	5.3 years	None	58	5326 (68)	Pravastatin (10)	No treatment
Schmermund et al, 2006^83^	467	1 year	Coronary atherosclerosis	62	276 (59)	Atorvastatin (10/80)	Different statin doses
ANDROMEDA, 2007^84^	509	4 months	Diabetes	62	199 (39)	Atorvastatin (10), rosuvastatin (10)	Different statin types
Bone et al, 2007^85^	604	1.1 years	None	59	604 (100)	Atorvastatin (10/20/40/80)	Placebo, different statin doses
Lewis et al, 2007^86^	326	9 months	Chronic liver disease	50	156 (48)	Pravastatin (80)	Placebo
METEOR, 2007^87^	981	2 years	Atherosclerosis	57	392 (40)	Rosuvastatin (40)	Placebo
JUPITER, 2008^30^	17 802	5 years	Raised C reactive protein	66	6765 (38)	Rosuvastatin (20)	Placebo
RCASS, 2009^88^	227	2 years	Cerebral atherosclerosis	63	152 (67)	Simvastatin (20)	Placebo
ASTRONOMER, 2010^89^	269	3.5 years	Aortic stenosis	58	102 (38)	Rosuvastatin (40)	Placebo
Eriksson et al, 2011^90^	352	3 months	None	60	113 (32)	Pitavastatin (4), simvastatin (40)	Different statin types
PATROL, 2011^91^	302	4 months	None	62	196 (65)	Atorvastatin (10), pitavastatin (2), rosuvastatin (2.5)	Different statin types
Ghia et al, 2013^92^	119	3 months	None	54	51 (43)	Atorvastatin (10/20)	Different statin doses
Stender et al, 2013^93^	942	3 months	None	70	528 (56)	Pitavastatin (1/2/4), pravastatin (10/20/40)	Different statin types and doses
STOMP, 2013^94^	420	6 months	None*	44	214 (51)	Atorvastatin (80)	Placebo
J-PREDICT, 2014^95^	1269	5 years	Impaired glucose tolerance*	56	482 (38)	Pitavastatin (1)	Placebo
LISTEN, 2014^96^	1018	1 year	Concurrent diabetes	66	550 (54)	Atorvastatin (10), rosuvastatin (5)	Different statin types
Sponseller et al, 2014^97^	328	3 months	None	58	164 (50)	Pitavastatin (4), pravastatin (40)	Different statin types
Nakagomi et al, 2015^98^	146	1 year	None	66	69 (47)	Atorvastatin (5), pitavastatin (1)	Different statin types
HOPE-3, 2016^31^	12 705	5.6 years	None	66	5844 (46)	Rosuvastatin (10)	Placebo
Patil et al, 2016^99^	100	2 months	None	60	49 (49)	Atorvastatin (20), pitavastatin (4)	Different statin types
Chen et al, 2018^100^	180	6 months	Cerebral atherosclerosis	61	81 (45)	Atorvastatin (20), pravastatin (20), rosuvastatin (10), simvastatin (40)	Different statin types
EMPATHY, 2018^101^	5042	5 years	Diabetic retinopathy	63	2622 (52)	Atorvastatin (7.6/13.1)†	Different statin doses
INTREPID, 2018^102^	252	1.1 years	HIV infection	50	35 (14)	Pitavastatin (4), pravastatin (40)	Different statin types
Liu et al, 2018^103^	180	6 months	Atherosclerosis	51	81 (45)	Atorvastatin (20), pravastatin (20), simvastatin (20)	Usual care, different statin types
BALANCE, 2019^104^	193	6 months	Diabetes	56	97 (50)	Rosuvastatin (5)	Placebo
METEOR-China, 2019^105^	540	2 years	Atherosclerosis	60	302 (56)	Rosuvastatin (20)	Placebo
Peng et al, 2019^106^	150	1 year	Renal artery atherosclerosis	64	57 (38)	Rosuvastatin (5/10)	Different statin doses
TRACE RA, 2019^107^	3002	5 years	Rheumatoid arthritis	61	2221 (74)	Atorvastatin (40)	Placebo
Moroi et al, 2020^108^	622	5 years	None	65	286 (46)	Atorvastatin (10), pitavastatin (2)	Different statin types
Thongtang et al, 2020^109^	150	3 months	Diabetes	59	108 (72)	Atorvastatin (40), simvastatin (20)	Different statin types

* STOMP and J-PREDICT trials did not use hyperlipidaemia or dyslipidaemia as a criterion of patient enrolment.

† A mixture of different statins and dosages was used in the trial, which were equivalent to atorvastatin 7.6 mg/day and 13.1 mg/day in the standard and intensive arms.

### Risk of bias and quality of evidence

**Fig 2:**
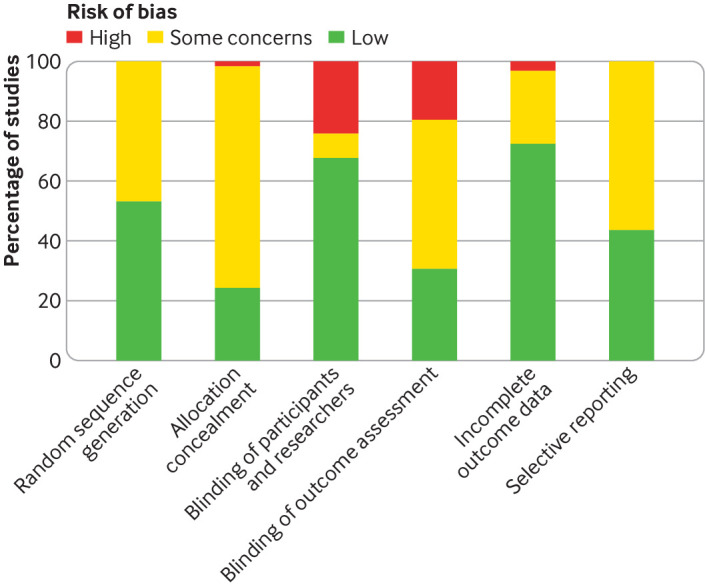
Summary of risk of bias across all included studies

Most of the included studies had a low or unclear risk of bias across all of the domains assessed (fig 2; supplementary fig 1). A few studies were judged to have a high risk of bias for blinding methods, most of which were comparisons between different statin regimens or reported clinically confirmed outcomes. For comparisons between statins and non-statin controls for the risk of self-reported muscle symptoms, which could be more susceptible to bias from blinding, only one small study with usual care control had an unclear risk of bias in blinding^103^ whereas the other 20 studies were placebo controlled trials with a low risk of bias in blinding.

The quality of evidence for comparisons between statins and non-statin controls for self-reported muscle symptoms, liver dysfunction, and the three major cardiovascular events was rated as high, with evidence for the remaining outcomes rated as moderate (table 2). In network meta-analyses, the quality of evidence for individual adverse effects of some types of statins was rated as high or moderate, whereas the quality of evidence for differences between statins was low (supplementary table 4).

**Table 2:** GRADE profile for pairwise meta-analyses

No of participants, No of RCTs; mean follow-up	Quality of evidence							Summary of findings		
	Risk of bias	Inconsistency	Indirectness	Imprecision	Publication bias	Overall certainty of evidence		Relative effect (OR (95% CI))	Anticipated absolute effect (event rate per 10 000 people throughout mean follow-up)	
									Risk with controls	Risk difference (95% CI) with statins
**Muscle symptoms**										
65 304, 21; 4.3 years	Not serious	Not serious	Not serious	Not serious	None	⨁⨁⨁⨁ High		1.06 (1.01 to 1.13)	951	56 (5 to 108)
**Muscle disorders**										
85 740, 25; 4.2 years	Not serious	Not serious	Not serious	Serious*	None	⨁⨁⨁◯ Moderate		0.88 (0.62 to 1.24)	14	−2 (−5 to 3)
**Liver dysfunction**										
54 803, 21; 3.8 years	Not serious	Not serious	Not serious	Not serious	None	⨁⨁⨁⨁ High		1.33 (1.12 to 1.58)	92	30 (11 to 53)
**Renal insufficiency**										
32 001, 8; 4.0 years	Not serious	Serious†	Not serious	Not serious	None	⨁⨁⨁◯ Moderate		1.14 (1.01 to 1.28)	343	45 (3 to 93)
**Diabetes**										
58 629, 9; 4.9 years	Not serious	Serious‡	Not serious	Not serious	None	⨁⨁⨁◯ Moderate		1.01 (0.88 to 1.16)	396	4 (−45 to 59)
**Eye conditions**										
25 328, 6; 3.8 years	Not serious	Serious§	Not serious	Not serious	None	⨁⨁⨁◯ Moderate		1.23 (1.04 to 1.47)	233	53 (8 to 105)
**Myocardial infarction**										
95 148, 22; 4.4 years	Not serious	Not serious	Not serious	Not serious	None¶	⨁⨁⨁⨁ High		0.72 (0.66 to 0.78)	292	−81 (−98 to−63)
**Stroke**										
78 473, 17; 4.7 years	Not serious	Not serious	Not serious	Not serious	None	⨁⨁⨁⨁ High		0.80 (0.72 to 0.89)	201	−39 (−55 to−22)
**Death from cardiovascular disease**										
95 959, 22; 4.4 years	Not serious	Not serious	Not serious	Not serious	None	⨁⨁⨁⨁ High		0.83 (0.76 to 0.91)	218	−36 (−52 to−19)

RCT=randomised controlled trial; OR=odds ratio; GRADE=Grading of Recommendations Assessment, Development, and Evaluation.

* The analysis was underpowered to detect a difference between groups, given the low incidences in both groups.

† Four studies reported the presence of proteinuria although the other four studies reported non-specific renal insufficiency.

‡ One study was conducted in patients with impaired glucose tolerance, which resulted in statistically significant (P=0.04) heterogeneity among the included studies.

§ One study reported cataracts, one reported diminished visual acuity, one reported eye inflammation, two reported non-specific eye and adnexa disorders, and one reported a combination of different eye conditions.

¶ Publication bias was detected by examining the asymmetry of the funnel plot for the pairwise meta-analysis of myocardial infarction, but the pooled results did not change after excluding the small studies that caused the detected bias.

### Associations of statins with adverse events

**Fig 3:**
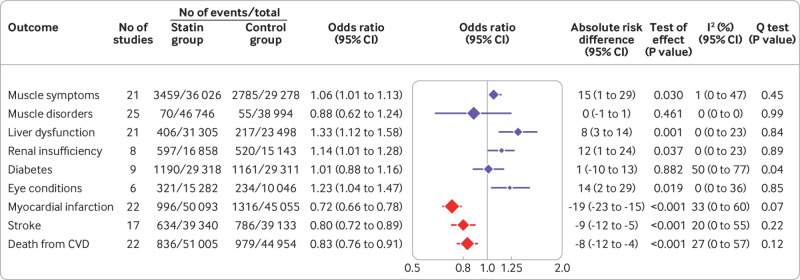
Associations of statins with safety and efficacy outcomes from pairwise meta-analyses. Symbols and horizontal bars represent pooled odds ratios with 95% confidence intervals calculated by pairwise meta-analyses, comparing statins and non-statin controls. Symbol sizes are proportional to the total numbers of participants included in the analyses of each outcome. Vertical line represents the odds ratio value that indicates no association (odds ratio=1). Blue symbols denote effects on safety outcomes (adverse events) and red symbols denote effects on efficacy outcomes (major cardiovascular events). Absolute risk difference is the number of events per 10 000 people in a year. CVD=cardiovascular disease

Thirty eight studies that compared statins with non-statin controls were included in the pairwise meta-analyses. We found no significant heterogeneity between individual studies and used a fixed effects model for all outcomes, with the exception of diabetes where a random effects model was used because significant heterogeneity (P=0.04; I^2^=50%; 95% confidence interval 0% to 77%) was detected (fig 3 and supplementary fig 2).

Statins were associated with a slightly increased risk of self-reported muscle symptoms (21 studies, odds ratio 1.06 (95% confidence interval 1.01 to 1.13); I^2^=1% (95% confidence interval 0% to 47%); fig 3), which mainly included myalgia (16 studies). But we found no association between statins and clinically confirmed muscle disorders. The influence analyses (supplementary fig 3) showed that the association between statins and muscle symptoms was largely determined by the double blind, placebo controlled HOPE-3 (Heart Outcomes Prevention Evaluation-3) trial,^31^ whereas the usual care controlled trial had little influence on the pooled result.^103^ Similarly, the no treatment controlled MEGA study (primary prevention of cardiovascular disease with pravastatin in Japan) had no influence on the association with muscle disorders.^82^

Statins increased the risk of liver dysfunction (21 studies, odds ratio 1.33 (95% confidence interval 1.12 to 1.58); I^2^=0% (95% confidence interval 0% to 23%)), which was defined as raised serum concentration of liver enzymes in all studies. Statins were also associated with renal insufficiency (eight studies, odds ratio 1.14 (1.01 to 1.28); I^2^=0% (0% to 23%)), which included the presence of proteinuria (four studies) and non-specified renal disorders (four studies), and with eye conditions (six studies, odds ratio 1.23 (1.04 to 1.47); I^2^=0% (0% to 36%)), which included cataracts (one study) and non-specified eye disorders (five studies) (fig 3). Influence analyses showed that the association with renal insufficiency was primarily determined by the JUPITER (Justification for the Use of Statin in Prevention: An Intervention Trial Evaluating Rosuvastatin) trial, which examined non-specified renal disorders,^30^ and the association with eye conditions was determined by the HOPE-3 trial, which examined cataracts.^31^

We did not detect significant publication bias for any safety outcome (supplementary fig 4). In sensitivity analyses (supplementary table 5), the pooled results from an alternative meta-analysis model were similar, and we found no substantial changes after excluding studies that involved some patients with cardiovascular disease.

### Comparison between beneficial and harmful effects of statins

For secondary outcomes of efficacy (fig 3 and supplementary fig 2), statins reduced the risks of myocardial infarction (22 studies, odds ratio 0.72 (95% confidence interval 0.66 to 0.78), I^2^=33% (95% confidence interval 0% to 60%)), stroke (17 studies, odds ratio 0.80 (0.72 to 0.89); I^2^=20% (0% to 55%)), and death from cardiovascular disease (22 studies, odds ratio 0.83 (0.76 to 0.91); I^2^=27% (0% to 57%)). Influence analyses suggested a larger reduction in risk for myocardial infarction and death from cardiovascular disease when the usual care controlled ALLHAT-LLT study (Antihypertensive and Lipid Lowering Treatment to Prevent Heart Attack Trial-Lipid Lowering Trial component) was excluded (supplementary fig 3).^61^ Publication bias was detected for myocardial infarction (P<0.05 for the test of asymmetry of the funnel plot) but the sensitivity analysis by excluding small studies showed a similar pooled effect (supplementary fig 4 and supplementary table 5).

Statins were estimated to induce 15 (95% confidence interval 1 to 29) more events of muscle symptoms, eight (3 to 14) more of liver dysfunction, 12 (1 to 24) more of renal insufficiency, and 14 (2 to 29) more of eye conditions per 10 000 patients treated for a year (fig 3). In contrast, statins were estimated to prevent 19 (15 to 23) myocardial infarctions, nine (5 to 12) strokes, and eight (4 to 12) deaths from cardiovascular disease per 10 000 patients treated for a year (table 2 shows the event rates throughout the duration of the trials).

### Differences in adverse effects between statin types

**Fig 4:**
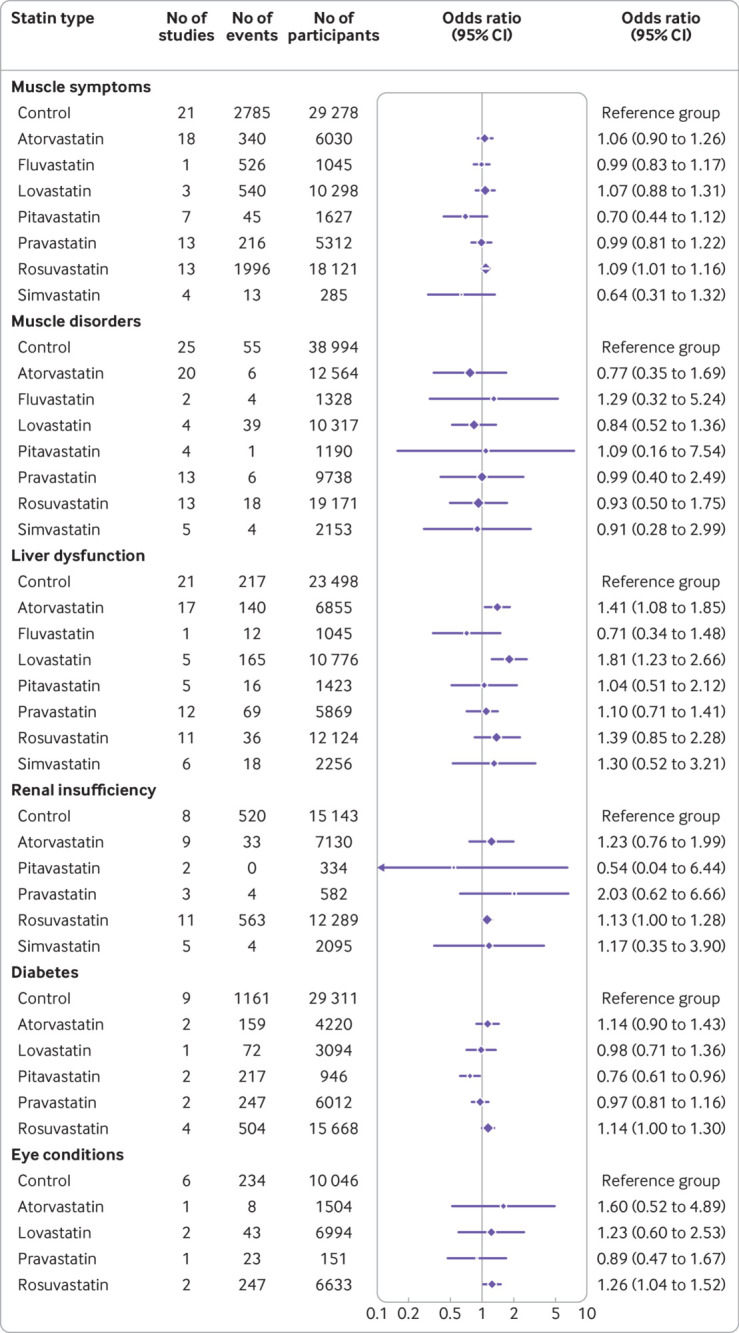
Associations of individual statins with adverse events from network meta-analyses. Symbols and horizontal bars represent pooled odds ratios with 95% confidence intervals derived from network meta-analyses, comparing individual statins with non-statin controls. Symbol sizes are proportional to the total numbers of participants included in the analyses of each statin type for each outcome. Vertical line represents the odds ratio value that indicates no association (odds ratio=1)

We included 58 studies to construct the networks of treatment comparisons for each safety outcome (supplementary fig 5). Rosuvastatin was associated with an increased risk of self-reported muscle symptoms (13 studies, odds ratio 1.09; 95% confidence interval 1.01 to 1.16)), renal insufficiency (11 studies, 1.13; 1.00 to 1.28), diabetes (four studies, 1.14; 1.00 to 1.30), and eye conditions (two studies, 1.26; 1.04 to 1.52) (fig 4). Atorvastatin (17 studies, 1.41; 1.08 to 1.85) and lovastatin (five studies, 1.81; 1.23 to 2.66) increased the risk of liver dysfunction. For comparisons between the different types of statins, lovastatin showed a higher risk of liver dysfunction than fluvastatin and pravastatin, and atorvastatin and rosuvastatin had a higher risk of diabetes than pitavastatin (supplementary table 6). We found no other significant differences between the types of statins.

We did not detect significant between study heterogeneity or between design inconsistency (P>0.05 for the generalised Q test) in the networks for any outcome (supplementary table 7), justifying the use of a fixed effects consistency model. Results from a random effects consistency model were similar (supplementary table 8). We found no significant inconsistencies between direct and indirect treatment comparisons in node splitting analyses (supplementary table 9).

### Dose-response relationships in adverse effects of statins

All 62 studies were included in the dose-response meta-analyses. A significant E_max_ dose-response relationship was only detected for the effect of atorvastatin on liver dysfunction, with a maximum effect that doubled the risk of liver dysfunction with non-statin controls (maximum odds ratio (OR_max_) 2.03; 95% credible interval 1.03 to 12.64) (table 3). No significant dose-response relationships were detected for other statins or adverse effects. E_max_ models were constructed based on the estimates of dose specific adverse effects of individual statins, which had low precision with wide 95% credible intervals and were available for only one or two dosages for some statins (supplementary fig 6 shows the E_max_ dose-response curves plotted on the dose specific adverse effects).

**Table 3:** Estimated maximum adverse effects of individual statins from E_max_ dose-response models*

Statin	Muscle symptoms	Muscle disorders	Liver dysfunction	Renal insufficiency	Diabetes	Eye conditions
Atorvastatin	1.35 (0.92 to 4.46)	0.89 (0.47 to 1.67)	2.03 (1.03 to 12.64)	1.35 (0.61 to 12.83)	1.18 (0.37 to 4.39)	1.32 (0.36 to 6.10)
Fluvastatin	1.10 (0.61 to 5.12)	1.12 (0.49 to 2.79)	1.02 (0.34 to 33.99)	No data	No data	No data
Lovastatin	1.25 (0.79 to 5.00)	0.85 (0.46 to 1.46)	3.29 (0.95 to 40.24)	No data	0.98 (0.14 to 5.63)	1.09 (0.38 to 2.83)
Pitavastatin	0.85 (0.43 to 2.01)	0.94 (0.27 to 2.62)	1.50 (0.41 to 100.85)	0.94 (0.27 to 4.70)	0.74 (0.19 to 3.34)	No data
Pravastatin	1.18 (0.79 to 4.84)	1.02 (0.58 to 1.76)	1.13 (0.59 to 14.72)	1.33 (0.60 to 4.57)	0.95 (0.25 to 3.77)	0.91 (0.26 to 3.33)
Rosuvastatin	1.26 (0.97 to 2.82)	0.92 (0.45 to 1.63)	1.61 (0.75 to 16.82)	1.39 (0.84 to 11.03)	1.16 (0.22 to 4.33)	1.36 (0.56 to 4.22)
Simvastatin	0.83 (0.44 to 2.58)	0.94 (0.44 to 2.06)	1.59 (0.60 to 21.95)	1.17 (0.48 to 5.20)	No data	No data

E_max_=asymptotic maximum drug effect.

* The maximum odds ratio (OR_max_) with 95% credible interval (CrI) in each cell is the maximum effect of each statin on the adverse event compared with non-statin controls (that is, the dose of the statin is 0), which is the natural exponential form of the estimated parameter, E_max_, in each model.

## Discussion

### Principal findings

In this systematic review of randomised controlled trials, we examined the associations between statins and adverse events in adults without a history of cardiovascular disease. We found a slightly increased risk of self-reported muscle symptoms after treatment with statins but no increased risk of clinically confirmed muscle disorders. Statins were associated with liver dysfunction, renal insufficiency, and eye conditions, but were not associated with diabetes. The absolute increases in the risks of these adverse events were small, and not comparable (numerically or clinically) with the reduction in the risk of major cardiovascular events achieved by treatment with statins.

Analyses by type of statin showed that atorvastatin, lovastatin, and rosuvastatin were associated with some adverse events, but few significant differences were seen between the statins. A possible modest dose-response relationship was identified for the effect of atorvastatin on liver dysfunction, but the pharmacological parameters were imprecise and the shape of the dose-response curve was unclear. The dose-response relationships for the other types of statins and adverse effects were inconclusive.

### Comparison with other studies

Most previous systematic reviews of trials examining statins for primary prevention did not find an association between statins and myalgia, myopathy, or rhabdomyolysis, based on small numbers of included studies and inconsistent definitions of outcomes.^22^ ^23^ A previous review found no association between statins and myalgia or other mild muscle symptoms (eg, muscle weakness and stiffness) but excluded some landmark trials, owing to a constrained study population.^25^ In contrast, a recent review showed an association between statins and overall muscle problems, but the review included a wide range of conditions with varying severities and incidences.^24^ In our review, we searched for more comprehensive data from studies, including those previously omitted,^94^ and classified muscle problems as self-reported symptoms or clinically confirmed disorders, to resolve the inconsistency and variety of definitions of outcomes in trials. This approach allowed us to clarify that statins are associated with a small increased risk of muscle symptoms, but the evidence for muscle disorders in patients with no history of cardiovascular disease was insufficient.

Attributing muscle symptoms to statins was originally identified in observational studies, but this association has been controversial, with some arguing that the higher risk of muscle symptoms in users of statins in routine practice is biased, because patients know they are receiving treatment and they might be aware of the potential adverse effects.^110^ ^111^ Our analysis of blinded, placebo controlled trials showed a smaller absolute increased risk of muscle symptoms than that reported in observational studies, supporting the view that most muscle symptoms reported by users of statins are nocebo effects and not actually caused by statins.^112^ An earlier review combining trials in primary and secondary prevention showed a similarly small absolute increased risk of myalgia, which was borderline significant.^113^

For clinically confirmed muscle disorders, previous reviews that included secondary prevention trials detected associations between statins and myopathy and rhabdomyolysis based on larger numbers of participants.^114^ ^115^ The analyses were still underpowered, however, given the low incidences of muscle disorders in the statin and control groups, similar to the data in our review.

Our results support the association between statins and liver dysfunction found in previous reviews, and this adverse effect was similar in primary and secondary prevention.^24^ ^116^ We also confirmed the associations of statins with renal insufficiency and eye conditions previously reported in primary prevention.^23^ ^24^ The diagnoses and measurements of these two outcomes in the included trials varied, and our influence analyses suggested that these associations might be limited to non-specific renal disorders and cataracts. In contrast, reviews that included studies in secondary prevention showed inverse or non-significant associations with a reduction in renal function and cataracts.^117^ ^118^ Nevertheless, trial data on specific renal and eye disorders are currently limited in both primary and secondary prevention.

We did not detect an association between statins and diabetes, similar to previous findings in primary prevention populations.^23^ ^24^ In a collaborative meta-analysis of secondary prevention trials, however, statins increased the risk of diabetes.^119^ This finding might be because the risk of diabetes is higher in a secondary prevention population or because the review included trials with larger sample sizes, older participants, and statin regimens with higher doses of statins.

Compared with rates seen in routine clinical practice, the baseline incidence of adverse events for patients included in trials might be lower because of differences in patient characteristics.^4^ ^120^ These differences could explain the inconsistency seen in the absolute risks of adverse events of treatment with statins between trials and observational studies. Even when estimates from trials and observational studies are taken into account, however, modelling studies suggest that the benefits of statins for primary prevention outweigh their potential harms in most patients eligible for treatment.^121^ In terms of clinical importance, most of the associated adverse events are self-reported, mild, or localised conditions, unlikely to lead to morbidity or mortality, compared with the major cardiovascular events that statins prevent.^1^ ^33^ ^112^ ^122^ ^123^

Only a few significant effects of individual types of statins were found for specific adverse events in this review (fig 4 and odds ratio values in the results section). The associations of atorvastatin and lovastatin with liver dysfunction could be because these statins are primarily metabolised in the liver.^124^ Rosuvastatin was associated with self-reported muscle symptoms, renal insufficiency, eye conditions, and diabetes, which might be because these risks are limited to rosuvastatin or because of larger sample sizes and higher doses in the trials of rosuvastatin. Comparisons between different types of statins showed considerable uncertainty, owing to insufficient data for several statins, especially fluvastatin, pitavastatin, and simvastatin. The few significant differences between statins could also be because of a false discovery rate potentially caused by the multiple tests conducted in these comparisons.^125^ This uncertainty of differences between statin was also found in previous reviews for primary or secondary prevention.^20^ ^24^

To our knowledge, a systematic review of the pharmacological dose-response relationships for adverse effects of statins has not been conducted. The only significant dose-response model established in our review suggested that the risk of liver dysfunction increased with increasing doses of atorvastatin. This trend supported the findings in a previous review that compared high dosages of statins with low dosages.^116^ But the estimated parameters in this model were imprecise and could not describe a clear pharmacological dose-response relationship. For other statins and adverse effects, estimates of E_max_ from the models made it difficult to draw any conclusions about the dose-response relationships. We also obtained ED_50_ values from these models, but the estimates showed considerable uncertainty and were not clinically relevant (specific results are available from the authors on request). This imprecision and uncertainty might be because of insufficient data on dosages for each statin or because of the low incidence of adverse events across the range of dosages. In the absence of dose-response relationships, a causal relation between statins and muscle symptoms is not well supported.^126^

### Strengths and limitations of the study

This large systematic review examined the association between adverse events and treatment with statins for primary prevention of cardiovascular disease. These analyses looked at the inconsistent reporting of muscle related adverse events in clinical trials and reported the risks of self-reported muscle symptoms and clinically confirmed muscle disorders in primary prevention patients. We used a newly developed method to examine the dose-response relationships of adverse effects of treatment with statins. Compared with other models, the E_max_ model reflects the fundamental pharmacodynamics of common inhibitors, such as statins, with clinically interpretable parameters.^127^

Despite these strengths, our review had limitations. Many of the analyses were underpowered to detect differences between groups, owing to the generally low incidence of adverse events and limited sample sizes. Some trials excluded vulnerable individuals more likely to have adverse events (eg, the ALLHAT-LLT trial excluded patients who were known to be intolerant of statins, and the CARDS and METEOR trials excluded patients who had high serum concentrations of creatinine),^61^ ^68^ ^87^ and many had short periods of follow-up (27 of the 62 included trials had a study duration of no more than six months). Therefore, the incidence of adverse events could have been underestimated, and more severe long term adverse events, such as substantial liver injury or renal failure, might have been missed. 

Because all of the included studies were primarily designed for evaluation of efficacy, data on adverse events might not have been systematically collected, although not collecting these data is unlikely to have biased the associations found. We transformed the event rates of each outcome throughout the study duration into annual incidences, which might be inaccurate in the absence of time-to-event data. A limitation of the use of study level aggregated data is that potential interactions between individual patient characteristics and effects of treatment cannot be accurately examined, and we had little information on the time of onset or duration of the adverse events, which might be useful for clinical practice. Most of the included trials enrolled patients with hyperlipidaemia or dyslipidaemia, which might not represent the general primary prevention population. Some trials enrolled a small number of patients with cardiovascular disease, although sensitivity analyses excluding these trials had little effect on the overall findings.

### Policy implications

The low risk of adverse events caused by statins reported in this review should reassure patients and physicians that the potential harms of statins are small and should not deter their use for primary prevention of cardiovascular disease. In particular, given the observed benefits of treatment in preventing major cardiovascular events, the slightly increased risk of self-reported muscle symptoms, which have no confirmed effect on physiological function, should not delay starting treatment with statins. For patients who do have muscle symptoms after treatment with statins, these data highlight that, in most cases, the symptoms are unlikely to be caused by treatment with statins alone. Physicians should therefore look at patients’ misconceptions of intolerance to statins and perhaps consider providing behavioural interventions, such as n-of-1 trials, to minimise unnecessary withdrawal of treatment.^128^ ^129^

The increased risk of liver dysfunction with statins suggests that routine monitoring of liver function during treatment is probably warranted in primary prevention, as recommended by the manufacturers in the statin product information. The current trial data do not support tailoring the type of statin or dosage to reduce adverse events in patients taking statins for primary prevention of cardiovascular disease.

To help improve adherence to statin treatment, studies are needed to identify patient characteristics that are crucial to these small risks of adverse events, which could be based on individual level data in clinical practice. These studies would also help with more targeted treatment and achieve more efficient monitoring. Future studies might also determine the associations of statins with more severe, long term adverse events, probably with observational and pharmacovigilance data from large populations, which might facilitate the detection of rare adverse events.

### Conclusions

Statins were associated with a small increased risk of self-reported muscle symptoms, liver dysfunction, renal insufficiency, and eye conditions in patients without a history of cardiovascular disease. These adverse effects were mild compared with the potential benefits of treatment with statins in preventing major cardiovascular events, suggesting that the benefit-to-harm balance of statins for primary prevention of cardiovascular disease is generally favourable. Evidence that these adverse effects varied by type or dosage of statins was limited, and therefore tailoring statin regimens before starting treatment to deal with concerns about safety is not currently supported.

**What is already known on this topic**

Although the efficacy of statins in preventing cardiovascular disease has been well established in previous systematic reviews, their potential adverse effects are inconclusive, particularly for muscle related adverse events, which have been inconsistently defined
The benefit-to-harm balance of statins has been shown to be highly favourable for secondary prevention of cardiovascular disease, but the use of statins in primary prevention is still controversial, owing to the lower risk of cardiovascular disease in this population
Current recommendations on the type and dosage of statins are based on their lipid lowering effects, without considering the varying adverse effects of different regimens

**What this study adds**

Based on data from placebo controlled blinded trials, for primary prevention of cardiovascular disease, a small proportion of self-reported muscle symptoms were attributable to statins, but no evidence of an association between statins and clinically confirmed muscle disorders was found
Adverse events associated with statins were mild and rare, and the absolute increase in the risk of these adverse events did not outweigh the reduction in the risk of major cardiovascular disease events, suggesting that the benefit-to-harm balance of statins for primary prevention of cardiovascular disease is favourable
Dose-response relationships between different types of statins and adverse effects were inconclusive, suggesting that tailoring statin regimens to deal with safety concerns when starting treatment is not currently needed

## Data Availability

Data sharing: Requests for data sharing should be sent to the corresponding author (james.sheppard@phc.ox.ac.uk).

## Web extra

Extra material supplied by authors

Web appendix: Supplementary material

## Notes

Contributors: TC and JPS conceived the study. TC designed the study and wrote the protocol with JPS, SL-F, JKA, RJS, and FDRH. TC developed the search strategies and ran the searches. TC, LA, and GM screened the literature for inclusion, extracted data from included studies, and assessed the quality of individual studies, under the supervision of JPS, SL-F, FDRH, and RJM. TC conducted the pairwise and network meta-analyses, under the supervision of JPS, SL-F, CK, and FDRH. TC conducted the dose-response meta-analyses, with methodology support from OL, under the supervision of JKA and RJS. TC and JPS drafted the manuscript. All authors contributed to critical revision for important intellectual content and approved the final version. JPS is the guarantor for this work and accepts full responsibility for the conduct of the study, had access to the data, and controlled the decision to publish. The corresponding author attests that all listed authors meet authorship criteria and that no others meeting the criteria have been omitted.

JPS affirms that the manuscript is an honest, accurate, and transparent account of the study being reported; that no important aspects of the study have been omitted; and that any discrepancies from the study as originally planned (and, if relevant, registered) have been explained.

Dissemination to participants and related patient and public communities: No participants were included in this work. The findings of this work, including a lay summary of the results, will be make available on the study website: www.phc.ox.ac.uk/research/stratified-treatments/studies/stratifying-treatments-in-the-multi-morbid-frail-elderly-stratify-statins.

Provenance and peer review: Not commissioned; externally peer reviewed.
