# Supplementary material for: Associations between statins and adverse events in primary prevention of cardiovascular disease: systematic review with pairwise, network, and dose-response meta-analyses
Source: BMJ. 2021 Jul 15;374:n1537. doi: 10.1136/bmj.n1537 (PMC8279037; doi:10.1136/bmj.n1537)

Supplementary Material

Associations between statins and adverse events in primary prevention of cardiovascular disease: systematic review with pair-wise, network, and dose-response meta-analyses

Ting Cai, Lucy Abel, Oliver Langford, Genevieve Monaghan, Jeffrey K Aronson, Richard Stevens, Sarah Lay-Flurrie, Constantinos Koshiaris, Richard J McManus, F D Richard Hobbs, James P Sheppard

Contents

Supplementary table 1. Search strategies in electronic bibliography databases

Supplementary table 2. Eligibility criteria of included studies

Supplementary table 3. List of studies excluded with reasons in full-text assessment

Supplementary figure 1. Risk of bias in individual studies

Supplementary table 4. GRADE profile for significant results from network meta-analyses

Supplementary figure 2. Forest plots of pair-wise meta-analyses

Supplementary figure 3. Leave-one-out influence analyses for pair-wise meta-analyses

Supplementary figure 4. Funnel plots of publication bias in pair-wise meta-analyses

Supplementary table 5. Sensitivity analyses for pair-wise meta-analyses

Supplementary figure 5. Networks of treatment comparisons in network meta-analyses

Supplementary table 6. Comparative adverse effects between different statin types from fixed-effect network meta-analyses (main analyses)

Supplementary table 7. Q tests of global heterogeneity and inconsistency in network meta-analyses

Supplementary table 8. Individual and comparative adverse effects of different statin types from random-effect network meta-analyses (sensitivity analyses)

Supplementary table 9. Node-splitting analyses of inconsistency between direct and indirect evidences in network meta-analyses

Supplementary figure 6. E_{max} dose-response curves with dose-specific adverse effects of individual statins

Supplementary table 1. Search strategies in electronic bibliography databases

**MEDLINE/PubMed**	
1	"hydroxymethylglutaryl-coa reductase inhibitors"[MeSH Terms]
2	statin[Title/Abstract] OR statins[Title/Abstract] OR atorvastatin[Title/Abstract] OR fluvastatin[Title/Abstract] OR lovastatin[Title/Abstract] OR pitavastatin[Title/Abstract] OR pravastatin[Title/Abstract] OR rosuvastatin[Title/Abstract] OR simvastatin[Title/Abstract]
3	lipitor[Title/Abstract] OR lescol[Title/Abstract] OR mevacor[Title/Abstract] OR livalo[Title/Abstract] OR pravachol[Title/Abstract] OR crestor[Title/Abstract] OR zocor[Title/Abstract]
4	1 OR 2 OR 3
5	"randomized controlled trial"[Publication Type] OR "controlled clinical trial"[Publication Type]
6	"randomized controlled trials as topic"[MeSH Terms] OR "controlled clinical trials as topic"[MeSH Terms]
7	trial[Title/Abstract] AND (random*[Title/Abstract] OR control*[Title/Abstract] OR placebo[Title/Abstract])
8	5 OR 6 OR 7
9	animals[MeSH Terms] NOT humans[MeSH Terms]
10	8 NOT 9
11	4 AND 10
12	niacin[Title/Abstract] OR niaspan[Title/Abstract] OR bile acid[Title/Abstract] OR cholestyramine[Title/Abstract] OR colesevelam[Title/Abstract] OR colestipol[Title/Abstract] OR ezetimibe[Title/Abstract] OR fibrate[Title/Abstract] OR fibrates[Title/Abstract]OR fenofibrate[Title/Abstract] OR gemfibrozil[Title/Abstract] OR PCSK9[Title/Abstract] OR alirocumab[Title/Abstract] OR evolocumab[Title/Abstract]
13	11 NOT 12
14	comment[Publication Type] OR congress[Publication Type] OR duplicate publication[Publication Type] OR editorial[Publication Type] OR letter[Publication Type] OR meta analysis[Publication Type] OR news[Publication Type] OR published erratum[Publication Type] OR review[Publication Type] OR systematic review[Publication Type]
15	protocol[Title]
16	14 OR 15
17	13 NOT 16
18	"2013/01/01"[Date - Publication] : "present"[Date - Publication]
19	17 AND 18
**Embase (Ovid)**	
1	hydroxymethylglutaryl coenzyme a reductase inhibitor.sh.
2	(statin? or atorvastatin or fluvastatin or lovastatin or pitavastatin or pravastatin or rosuvastatin or simvastatin).ti,ab.
3	(lipitor or lescol or mevacor or livalo or pravachol or crestor or zocor).ti,ab.

4	1 or 2 or 3
5	limit 4 to (randomized controlled trial or controlled clinical trial)
6	exp animals/ not exp humans/
7	5 not 6
8	(niacin or niaspan or bile acid or cholestyramine or colesevelam or colestipol or ezetimibe or fibrate? or fenofibrate or gemfibrozil or PCSK9 or alirocumab or evolocumab).ti,ab.
9	7 not 8
10	(abstract or conference abstract or "conference review" or editorial or erratum or letter or note or "review").pt.
11	(comment* or protocol).ti.
12	10 or 11
13	9 not 12
14	limit 13 to yr="2013 - Current"
**CENTRAL (Cochrane Library)**	
1	(statin OR statins OR atorvastatin OR fluvastatin OR lovastatin OR pitavastatin OR pravastatin OR rosuvastatin OR simvastatin) in Record Title
2	(niacin or niaspan or bile acid or cholestyramine or colesevelam or colestipol or ezetimibe or fibrate or fibrates or fenofibrate or gemfibrozil or PCSK9 or alirocumab or evolocumab) in Title Abstract Keyword
3	1 NOT 2
With search limits:	
o	Content type - Trials
o	Trials original publication year - from 2013 to 2020
o	Search word variations - Yes

*** All searches were updated by 1 August 2020.**

Supplementary table 2. Eligibility criteria of included studies

	Inclusion Criteria	Exclusion Criteria
**Study design/ Settings**	Randomised controlled clinical trial	• Study duration shorter than four weeks<br>• Cross-over or self-controlled study designs<br>• Re-analyses/subgroup analyses of the same trial data, or post-trial analyses of observational data from extended follow-up which did not adhere to the initial randomisation<br>• Examination of the pleiotropy of statins for non-CVD conditions (e.g. cancer, chronic obstructive pulmonary disease, contrast-induced nephropathy)<br>• Uncompleted or withdrawn studies
**Participants**	• Adults (over 18 years old) without existing diagnoses of CVD, previous cardiovascular events, or history of cardiac surgery<br>• If a small proportion of CVD patients was involved, the proportion should be no more than 30%	Number of participants less than 100
**Interventions**	• Any of the seven types of statins in clinical use, including Atorvastatin, Fluvastatin, Lovastatin, Pitavastatin, Pravastatin, Rosuvastatin, and Simvastatin<br>• Statins could be used in any doses<br>• Statins could be applied as monotherapy or add-on treatment to other interventions	Statins were used in combination with other lipid-lowering medications
**Comparators**	• Non-statin controls: placebo, usual care, non-pharmaceutical treatment (e.g. dietary management, physical exercise), or no treatment<br>• Statin comparators: different types or doses of statins	Comparison of different formulations (e.g. oral vs. topical) of one statin type at the same dose
**Outcomes**	Primary outcomes:<br>• Self-reported muscle symptoms<br>• Clinically-confirmed muscle disorders<br>• Live dysfunction<br>• Renal insufficiency<br>• Diabetes<br>• Eye conditions<br>Secondary outcomes:<br>• Myocardial infarction<br>• Stroke<br>• Death from CVD	No desired outcome data available, either effect estimate with standard error for compared groups or number/rate of events in each group

Supplementary table 3. List of studies excluded with reasons in full-text assessment

ID	Citation	Reason for exclusion
**Studies identified from previous reviews**		
1	Downs JR, Clearfield M, Tyroler HA, et al. Air Force/Texas Coronary Atherosclerosis Prevention Study (AFCAPS/TexCAPS): Additional perspectives on tolerability of long-term treatment with lovastatin. American Journal of Cardiology. 2001;87(9):1074-1079.	Duplicate publication of same data
2	Salonen R, Nyyssonen K, Porkkala-Sarataho E, Salonen JT. The kuopio atherosclerosis prevention study (KAPS): Effect of pravastatin treatment on lipids, oxidation resistance of lipoproteins, and atherosclerotic progression. American Journal of Cardiology. 1995;76(9):34C-39C.	
3	Alcala A, Jansen S, Tellez T, et al. Statins improve visual field alterations related to hypercholesterolemia. Atherosclerosis. 2010;209(2):510-514.	No report on the outcomes of interest
4	Hedblad B, Wikstrand J, Janzon L, Wedel H, Berglund G. Low-dose metoprolol CR/XL and fluvastatin slow progression of carotid intima-media thickness: Main results from the beta-blocker cholesterol-lowering asymptomatic plaque study (BCAPS). Circulation. 2001;103(13):1721-1726.	
5	Lindholm LH, Ekbom T, Dash C, Isacsson A, Schersten B. Changes in cardiovascular risk factors by combined pharmacological and nonpharmacological strategies: The main results of the CELL study. Journal of Internal Medicine. 1996;240(1):13-22.	
6	Weir MR, Berger ML, Weeks ML, Liss CL, Santanello NC. Comparison of the effects on quality of life and of the efficacy and tolerability of lovastatin versus pravastatin. American Journal of Cardiology. 1996;77(7):475-479.	
7	Asselbergs FW, van der Harst P, van Roon AM, et al. Long-term effects of pravastatin and fosinopril on peripheral endothelial function in albuminuric subjects. Atherosclerosis. 2008;196(1):349-355.	Re-analysis of existing trials (post-trial data/subgroup analysis/additional non-interested outcomes)
8	Ford I, Murray H, McCowan C, Packard CJ. Long-term safety and efficacy of lowering low-density lipoprotein cholesterol with statin therapy 20-year follow-up of west of Scotland coronary prevention study. Circulation. 2016;133(11):1073-1080.	
9	Han BH, Sutin D, Williamson JD, et al. Effect of statin treatment vs usual care on primary cardiovascular prevention among older adults: The ALLHAT-LLT randomized clinical trial. JAMA Internal Medicine. 2017;177(7):955-965.	
10	Sever PS, Chang CL, Gupta AK, Whitehouse A, Poulter NR. The Anglo-Scandinavian Cardiac Outcomes Trial: 11-year mortality follow-up of the lipid-lowering arm in the UK. European Heart Journal. 2011;32(20):2525-2532.	
11	Sever PS, Poulter NR, Dahlof B, et al. Reduction in cardiovascular events with atorvastatin in 2,532 patients with type 2 diabetes: Anglo-Scandinavian Cardiac Outcomes Trial-Lipid-Lowering Arm (ASCOT-LLA). Diabetes Care. 2005;28(5):1151-1157.	
12	Derosa G, Mugellini A, Ciccarelli L, Fogari R. Randomized, double-blind, placebo-controlled comparison of the action of orlistat, fluvastatin, or both on anthropometric measurements, blood pressure, and lipid profile in obese patients with hypercholesterolemia prescribed a standardized diet. Clinical Therapeutics. 2003;25(4):1107-1122.	Sample size <100
13	Heljic B, Velija-Asimi Z, Kulic M. The statins in prevention of coronary heart diseases in type 2 diabetics. Bosnian journal of basic medical sciences / Udruzenje basicnih mediciniskih znanosti = Association of Basic Medical Sciences. 2009;9(1):71-76.	
14	Behounek BD, McGovern ME, Kassler-Taub KB, et al. Effects of pravastatin in patients with serum total cholesterol levels from 5.2 to 7.8 mmol/liter (200 to 300 mg/dl) plus two additional atherosclerotic risk factors. American Journal of Cardiology. 1993;72(14):1031-1037.	Secondary prevention population

15	Collins R, Armitage J, Parish S, Sleight P, Peto R. MRC/BHF Heart Protection Study of cholesterol lowering with simvastatin in 20 536 high-risk individuals: A randomised placebo-controlled trial. Lancet. 2002;360(9326):7-22.	
16	Cowell SJ, Newby DE, Prescott RJ, et al. A randomized trial of intensive lipid-lowering therapy in calcific aortic stenosis. New England Journal of Medicine. 2005;352(23):2389-2397.	
17	Fellstrom BC, Jardine AG, Schmieder RE, et al. Rosuvastatin and cardiovascular events in patients undergoing hemodialysis. New England Journal of Medicine. 2009;360(14):1395-1407.	
18	Kurabayashi M, Yamazaki T. Superior benefit of aggressive lipid-lowering therapy for high-risk patients using statins: The SUBARU study - More hypercholesterolemic patients achieve Japan atherosclerosis society LDL-C goals with rosuvastatin therapy than with atorvastatin therapy. Journal of Atherosclerosis and Thrombosis. 2008;15(6):314-323.	
19	Sawayama Y, Shimizu C, Maeda N, et al. Effects of probucol and pravastatin on common carotid atherosclerosis in patients with asymptomatic hypercholesterolemia: Fukuoka atherosclerosis trial (FAST). Journal of the American College of Cardiology. 2002;39(4):610-616.	
20	Shepherd J, Blauw GJ, Murphy MB, et al. Pravastatin in elderly individuals at risk of vascular disease (PROSPER): A randomised controlled trial. Lancet. 2002;360(9346):1623-1630.	
21	Stegmayr BG, Brannstrom M, Bucht S, et al. Low-dose atorvastatin in severe chronic kidney disease patients: A randomized, controlled endpoint study. Scandinavian Journal of Urology and Nephrology. 2005;39(6):489-497.	
22	Stein EA, Davidson MH, Dobs AS, et al. Efficacy and safety of Simvastatin 80 mg/day in hypercholesterolemic patients. American Journal of Cardiology. 1998;82(3):311-316.	
23	Wanner C, Krane V, Marz W, et al. Atorvastatin in patients with type 2 diabetes mellitus undergoing hemodialysis. New England Journal of Medicine. 2005;353(3):238-248.	
24	Beishuizen ED, Van De Ree MA, Jukema JW, et al. Two-year statin therapy does not alter the progression of intima-media thickness in patients with type 2 diabetes without manifest cardiovascular disease. Diabetes Care. 2004;27(12):2887-2892.	Study of cerivastatin
**Studies identified from database searches**		
1	Kitas GD, Nightingale P, Armitage J, Sattar N, Belch J, Symmons D. Trial of atorvastatin for the primary prevention of cardiovascular events in patients with rheumatoid arthritis (TRACE RA). Annals of the rheumatic diseases. 2015;74:688.	Duplicate publication of same data
2	Odawara M, Yamazaki T, Kishimoto J, et al. Effect of pitavastatin on the incidence of diabetes in Japanese individuals with impaired glucose tolerance. Diabetologia. 2013;56:S59-.	
3	Sponseller CA, Morgan R, Campbell S, et al. Pitavastatin 4 mg Provides Superior LDL-C Reduction vs. Pravastatin 40 mg Over 12 weeks in HIV-Infected Adults with Dyslipidemia, the INTREPID Trial. Journal of clinical lipidology. 2013;7(3):260.	
4	Trial of Atorvastatin for the Primary Prevention of Cardiovascular Events in Patients with RA (TRACE RA): A Randomized Trial in 2986 RA Patients. Rheumatology. 2015;54:i87-.	
5	Abe M, Maruyama N, Maruyama T, Okada K, Soma M. A Trial of Pitavastatin Versus Rosuvastatin for Dyslipidemia in Chronic Kidney Disease. J Atheroscler Thromb. 2015;22(12):1235-1247.	No report on the outcomes of interest
6	Biedermann JS, Kruip M, van der Meer FJ, et al. Rosuvastatin use improves measures of coagulation in patients with venous thrombosis. Eur Heart J. 2018;39(19):1740-1747.	
7	Chang CT, Lee JK, Lin JD, et al. The lipid-lowering effect of atorvastatin in Taiwanese diabetic patients with hyperlipidemia. Tzu Chi Medical Journal. 2013;25(3):168-174.	
8	Chen F, Wang SP. Effects of atorvastatin combined with insulin glargine on platelet activation in diabetic patients with hyperlipidemia. [Chinese]. Chinese Journal of Pharmaceutical Biotechnology. 2019;26(5):409-412.	

9	Euctr CZ. The efficacy and safety of a medicine containing the active substance rosuvastatin. http://wwwwhoint/trialsearch/Trial2aspx?TrialID=EUCTR2012-004799-21-CZ. 2013.	
10	Gong J, Sun H, Yang Z, Liu M, Ma L, Song M. Effect of simvastatin and relationship between bilirubin and blood lipid level in patients with glucocorticoid-resistant nephrotic syndrome. West Indian medical journal. 2015:epub ahead.	
11	He H, Jin J, Xue S, Chen J, Gu Y, Zhang S. The effect of long-term application of small doses atorvastatin in elderly patients with hyperlipidemia on serum uric acid. Journal of the american college of cardiology. 2015;66(16 SUPPL. 1):C231.	
12	Ji T, Zhao Y, Wang J, et al. Effect of Low-Dose Statins and Apolipoprotein E Genotype on Cerebral Small Vessel Disease in Older Hypertensive Patients: A Subgroup Analysis of a Randomized Clinical Trial. J Am Med Dir Assoc. 2018;19(11):995-1002.e1004.	
13	Jprn U. Investigation oflipid-improving and pleiotrophic effects of pitavastatin. http://wwwwhoint/trialsearch/Trial2aspx?TrialID=JPRN-UMIN000019020. 2015.	
14	Koh K. Rosuvastatin dose-dependently improves flow-mediated dilation, but reduces adiponectin levels and insulin sensitivity in hypercholesterolemic patients. European heart journal. 2018;39:492-493.	
15	Longenecker CT, Hileman CO, Funderburg NT, McComsey GA. Rosuvastatin preserves renal function and lowers cystatin C in HIV-infected subjects on antiretroviral therapy: the SATURN-HIV trial. Clin Infect Dis. 2014;59(8):1148-1156.	
16	Longenecker CT, Jiang Y, Debanne SM, et al. Rosuvastatin arrests progression of carotid intima-media thickness in treated HIV. Topics in antiviral medicine. 2015;23:55-56.	
17	Longenecker CT, Sattar A, Gilkeson R, McComsey GA. Rosuvastatin slows progression of subclinical atherosclerosis in patients with treated HIV infection. Aids. 2016;30(14):2195-2203.	
18	Miyoshi T, Kohno K, Osawa K, et al. Effect of statin on the progression of coronary artery calcification detected by computed tomography (peach trial). Journal of cardiovascular computed tomography. 2015;9(4 SUPPL. 1):S12-S13.	
19	Moriarty PM, Sponseller C, Backes J, et al. Pitavastatin lowers plasma levels of CoQ10 less than equipotent doses of rosuvastatin or atorvastatin. European heart journal. 2016;37:110-.	
20	Nebieridze D. The additional antihypertensive effect of statins in hypertensive patients with moderate risk. Journal of hypertension. 2015;33:e320.	
21	Nishikura T, Akabayashi K, Noue T, et al. Biomarkers associated with dense calcium increase in coronary plaque under statin therapy. Circulation. 2017;136.	
22	Oh GC, Han JK, Han KH, et al. Efficacy and Safety of Fixed-dose Combination Therapy With Telmisartan and Rosuvastatin in Korean Patients With Hypertension and Dyslipidemia: TELSTA-YU (TELmisartan-rosuvaSTAtin from YUhan), a Multicenter, Randomized, 4-arm, Double-blind, Placebo-controlled, Phase III Study. Clin Ther. 2018;40(5):676-691.e671.	
23	Qiaotao X, Yang X, Liu J. Effect of the rosuvastatin on pulse pressure and arterial stiffness in aged patients with isolated systolic hypertension without hyperlipidemia. Journal of hypertension. 2018;36:e130-e131.	
24	Rafeeq MM, Habib HS, Murad H, Gari MA, Gazzaz ZJ. Effect of rosuvastatin on dyslipidemia and other parameters associated with metabolic syndrome in Saudi patients. Niger J Clin Pract. 2017;20(4):445-453.	
25	Rizos CV, Liberopoulos EN, Mpilianou E, et al. The effects of atorvastatin 30 mg compared with atorvastatin 40 mg in patients with primary hyperlipidemia: A multicenter study. Hellenic Journal of Atherosclerosis. 2018;9(2):8-17.	
26	Shou FY, Zhao ZH, Yang FF, Wang HM, Hu LY. Evaluation of the efficacy of rosuvastatin calcium in elderly patients with hyperlipidemia and hypertension (HTN). Journal of the american geriatrics society. 2013;61:S340.	

27	Shuiping Z. Comparison of the efficacy of rosuvastatin vs atorvastatin on LDL cholesterol in Chinese patients with hypercholesterolemia: a multicenter, randomized, double blind trial. Journal of the american college of cardiology. 2016;68(16):C77-C78.	
28	Soran H, Liu Y, Adam S, et al. A comparison of the effects of low- and high-dose atorvastatin on lipoprotein metabolism and inflammatory cytokines in type 2 diabetes: Results from the Protection Against Nephropathy in Diabetes with Atorvastatin (PANDA) randomized trial. J Clin Lipidol. 2018;12(1):44-55.	
29	Strazhesko ID, Tkacheva ON, Dudinskaya EN, et al. Atorvastatin modulates telomerase activity in patients free of cardiovascular diseases. European heart journal. 2016;37:934-935.	
30	Talavera JO, Martinez G, Cervantes JL, et al. A double-blind, double-dummy, randomized, placebo-controlled trial to evaluate the effect of statin therapy on triglyceride levels in Mexican hypertriglyceridemic patients. Curr Med Res Opin. 2013;29(4):379-386.	
31	Tani S, Takahashi A, Nagao K, Hirayama A. Contribution of apolipoprotein A-I to the reduction in high-sensitivity C-reactive protein levels by different statins: comparative study of pitavastatin and atorvastatin. Heart Vessels. 2015;30(6):762-770.	
32	Tsikas D, Pham VV, Suchy MT, et al. No effects of atorvastatin (10 mg/d or 80 mg/d) on nitric oxide, prostacyclin, thromboxane and oxidative stress in type 2 diabetes mellitus patients of the DALI study. Pharmacol Res. 2015;94:1-8.	
33	Wang X, Wang KQ, Dong Y, Shen XZ. Effects of rosuvastatin on the carotid vulnerable plaque in the elderly patients. Journal of the american geriatrics society. 2015;63:S342.	
34	Yazbek DC, de Carvalho AB, Barros CS, Medina Pestana JO, Canziani ME. Effect of Statins on the Progression of Coronary Calcification in Kidney Transplant Recipients. PLoS One. 2016;11(4):e0151797.	
35	Zhao Z, Niu X, Dong Z, et al. Upstream therapeutic strategies of valsartan and fluvastatin on hypertensive patients with non-permanent atrial fibrillation. Cardiovasc Ther. 2018;36(6):e12478.	
36	Fan P, Lu P, Zhang Y, et al. Major outcomes of simvastatin in high-risk hypertensive patients with a high-normal level of total cholesterol: from the chinese hypertension intervention efficacy study (CHIEF). Journal of hypertension. 2018;36:e314-.	No specific outcome data
37	Kim TS. Efficacy/Safety of Telmisartan/Amlodipine/Rosuvastatin in Hypertensive Patients With Hyperlipidemia. https://clinicaltrialsgov/show/NCT03566316. 2018.	
38	Lu PP, Ma LH, Ma LY, et al. Cardiovascular benefits of diuretic-based therapies combined with simvastatin in high-risk hypertensive patients with a high-normal level of total cholesterol. Cardiology (switzerland). 2018;140:40-.	
39	Thongtang N, Sitthananun C, Sriussadaporn S, Nitiyanant W. Efficacy of low- and moderate-intensity statins for achieving low- density lipoprotein cholesterol targets in Thai type 2 diabetic patients. Journal of Diabetes and Metabolic Disorders. 2017;16(1).	Not RCT
40	Dagenais GR, Jung H, Lonn E, et al. Effects of Lipid-Lowering and Antihypertensive Treatments in Addition to Healthy Lifestyles in Primary Prevention: An Analysis of the HOPE-3 Trial. J Am Heart Assoc. 2018;7(15).	Re-analysis of existing trials (post-trial data/subgroup analysis/additional non-interested outcomes)
41	Gupta A, Mackay J, Whitehouse A, et al. Long-term mortality after blood pressure-lowering and lipid-lowering treatment in patients with hypertension in the Anglo-Scandinavian Cardiac Outcomes Trial (ASCOT) Legacy study: 16-year follow-up results of a randomised factorial trial. Lancet. 2018;392(10153):1127-1137.	
42	Pais P, Jung H, Dans A, et al. Impact of blood pressure lowering, cholesterol lowering and their combination in Asians and non-Asians in those without cardiovascular disease: an analysis of the HOPE 3 study. Eur J Prev Cardiol. 2019;26(7):681-697.	
43	Tada H, Kawashiri MA, Nomura A, et al. Serum triglycerides predict first cardiovascular events in diabetic patients with hypercholesterolemia and retinopathy. Eur J Prev Cardiol. 2018;25(17):1852-1860.	

44	Verbree-Willemsen L, Zhang YN, Gijsberts CM, et al. LDL extracellular vesicle coagulation protein levels change after initiation of statin therapy. Findings from the METEOR trial. Int J Cardiol. 2018;271:247-253.	
45	Yan R, Gu HQ, Wang W, Ma L, Li W. Health-related quality of life in blood pressure control and blood lipid-lowering therapies: results from the CHIEF randomized controlled trial. Hypertens Res. 2019;42(10):1561-1571.	
46	Irct2017010631252N. Effect of Atorvastatin on liver function in patients with chronic hepatitis B. http://wwwwhoint/trialsearch/Trial2aspx?TrialID=IRCT2017010631252N3. 2017.	Sample size <100
47	Kim W, Chang K, Cho EJ, et al. A randomized, double-blind clinical trial to evaluate the efficacy and safety of a fixed-dose combination of amlodipine/rosuvastatin in patients with dyslipidemia and hypertension. J Clin Hypertens (Greenwich). 2020.	
48	Koonarat A. Cardioprotective Effect of Atorvastatin in Lymphoma Patients Receiving CHOP/R-CHOP regimen, A Randomized Control Trial. http://wwwwhoint/trialsearch/Trial2aspx?TrialID=TCTR20180202004. 2018.	
49	Lata S, Gupta BM, Bhat NK, Bhat S, Kumar D. A randomized, open label, comparative, prospective study evaluating efficacy, safety and compliance of rosuvastatin versus atorvastatin in overweight and obese dyslipidemic patients. JK Science. 2017;19(1):17-21.	
50	Nct. A Comparative Study of Rosuvastatin and Atorvastatin in Patients With Hyperlipidemia. https://clinicaltrialsgov/show/NCT02979704. 2016.	
51	Nct. Effect of Rosuvastatin on Coronary Flow Reserve in Hypertensive Patients With Cardiovascular Risk. https://clinicaltrialsgov/show/NCT02482207. 2015.	
52	Nct. Effects of Pitavastatin on Lipid Profiles in HIV-infected Patients With Dyslipidemia and Receiving Atazanavir/Ritonavir. https://clinicaltrialsgov/show/NCT02442700. 2015.	
53	Agrawal D, Manchanda SC, Sawhney JPS, et al. To study the effect of high dose Atorvastatin 40 mg versus 80 mg in patients with dyslipidemia. Indian Heart Journal. 2018;70(Supplement 3):S8-S12.	Secondary prevention population
54	Hong SJ, Jeong HS, Cho JM, et al. Efficacy and Safety of Triple Therapy With Telmisartan, Amlodipine, and Rosuvastatin in Patients With Dyslipidemia and Hypertension: The Jeil Telmisartan, Amlodipine, and Rosuvastatin Randomized Clinical Trial. Clin Ther. 2019;41(2):233-248.e239.	
55	Ikeda K, Takahashi T, Yamada H, et al. Effect of intensive statin therapy on regression of carotid intima-media thickness in patients with subclinical carotid atherosclerosis (a prospective, randomized trial: PEACE (Pitavastatin Evaluation of Atherosclerosis Regression by Intensive Cholesterol-lowering Therapy) study). Eur J Prev Cardiol. 2013;20(6):1069-1079.	
56	Kim TS, Rha SW, Kim SY, et al. Efficacy and Tolerability of Telmisartan/Amlodipine and Rosuvastatin Coadministration in Hypertensive Patients with Hyperlipidemia: A Phase III, Multicenter, Randomized, Double-blind Study. Clinical Therapeutics. 2019;41(4):728-741.	
57	Liu PY, Lin LY, Lin HJ, et al. Pitavastatin and Atorvastatin double-blind randomized comPArative study among hiGh-risk patients, including thOse with Type 2 diabetes mellitus, in Taiwan (PAPAGO-T Study). PLoS One. 2013;8(10):e76298.	
58	Nct. A High-Resolution Magnetic Resonance Imaging Study to Evaluate the Effect of Rosuvastatin on Carotid Atherosclerotic Plaques. https://clinicaltrialsgov/show/NCT02305862. 2014.	
59	Nct. to Evaluate the Safety and Efficacy of Pitavastatin in Patients With IFG and Hyperlipidemia. https://clinicaltrialsgov/show/NCT02056847. 2014.	
60	Nishikido T, Oyama J, Keida T, Ohira H, Node K. High-dose statin therapy with rosuvastatin reduces small dense LDL and MDA-LDL: The Standard versus high-dose therApy with Rosuvastatin for lipiD lowering (SARD) trial. J Cardiol. 2016;67(4):340-346.	
61	Park SJ, Kang SJ, Ahn JM, et al. Effect of Statin Treatment on Modifying Plaque Composition: a Double-Blind, Randomized Study. Journal of the American College of Cardiology. 2016;67(15):1772-1783.	

62	Sasaki J, Otonari T, Uchida Y, Ikeda Y, Biro S, Kono S. Effects of pravastatin and atorvastatin on HDL cholesterol and glucose metabolism in patients with dyslipidemia and glucose intolerance: the PRAT study. J Atheroscler Thromb. 2013;20(4):368-379.	
63	Tian CF, Su BY, Tong YH, et al. Protective effects of statins on renal function in patients with carotid atherosclerotic plaques. Acta Medica Mediterranea. 2018;34(4):1003-1007.	
64	Zhao S, Peng D. Efficacy and safety of rosuvastatin versus atorvastatin in high-risk Chinese patients with hypercholesterolemia: a randomized, double-blind, active-controlled study. Curr Med Res Opin. 2018;34(2):227-235.	
65	Zhao S, Peng D. Efficacy and safety of rosuvastatin versus atorvastatin in high-risk Chinese patients with hypercholesterolemia: a randomized, double-blind, active-controlled study. Curr Med Res Opin. 2018;34(2):227-235.	
66	Ahmad J. To study the effects of cholesterol lowering drugs (Atorvastatin and Rosuvastatin) on blood glucose levels. http://wwwwhoint/trialsearch/Trial2aspx?TrialID=CTRI/2018/05/013991. 2018.	Uncompleted trial/No published results
67	Jprn U. Evaluation for Efficacy of Rosuvastatin on Carotid Intima Media Thickness and Characteristics of Atherosclerosis in Hyperlipidemic Patients with Type 2 Diabetes. http://wwwwhoint/trialsearch/Trial2aspx?TrialID=JPRN-UMIN000017198. 2015.	
68	Jprn U. Evaluation of the effect of rosuvastatin on renoprotective effect. http://wwwwhoint/trialsearch/Trial2aspx?TrialID=JPRN-UMIN000021583. 2016.	
69	Malek M. Comparison of efficacy of low vs high dose of Atorvastatin and Rosuvastatin in reducing 50% LDL in diabetic patient. http://wwwwhoint/trialsearch/Trial2aspx?TrialID=IRCT20180929041169N1. 2019.	
70	Nct. A Clinical Trial of STAtin Therapy for Reducing Events in the Elderly (STAREE). https://clinicaltrialsgov/show/NCT02099123. 2014.	
71	Nct. Effect of Rosuvastatin Therapy on HDL2 Level. https://clinicaltrialsgov/show/NCT02593487. 2015.	
72	Nct. Evaluating the Use of Pitavastatin to Reduce the Risk of Cardiovascular Disease in HIV-Infected Adults. https://clinicaltrialsgov/show/NCT02344290. 2015.	
73	Nct. Statins for the Primary Prevention of Heart Failure in Patients Receiving Anthracycline Pilot Study. https://clinicaltrialsgov/show/NCT03186404. 2017.	
74	Tctr. Comparative Study of Efficacy and Adverse Effects of Triphala versus Simvastatin in Blood Lipids Reduction in Dyslipidemia Patients. http://wwwwhoint/trialsearch/Trial2aspx?TrialID=TCTR20140523002. 2014.	
75	Wang JN. Efficacy and Safety of HS-25 or in Combination With Atorvastatin in Chinese Adults With Primary Hypercholesterolemia. https://clinicaltrialsgov/show/NCT03464682. 2018.	

Supplementary figure 1. Risk of bias in individual studies

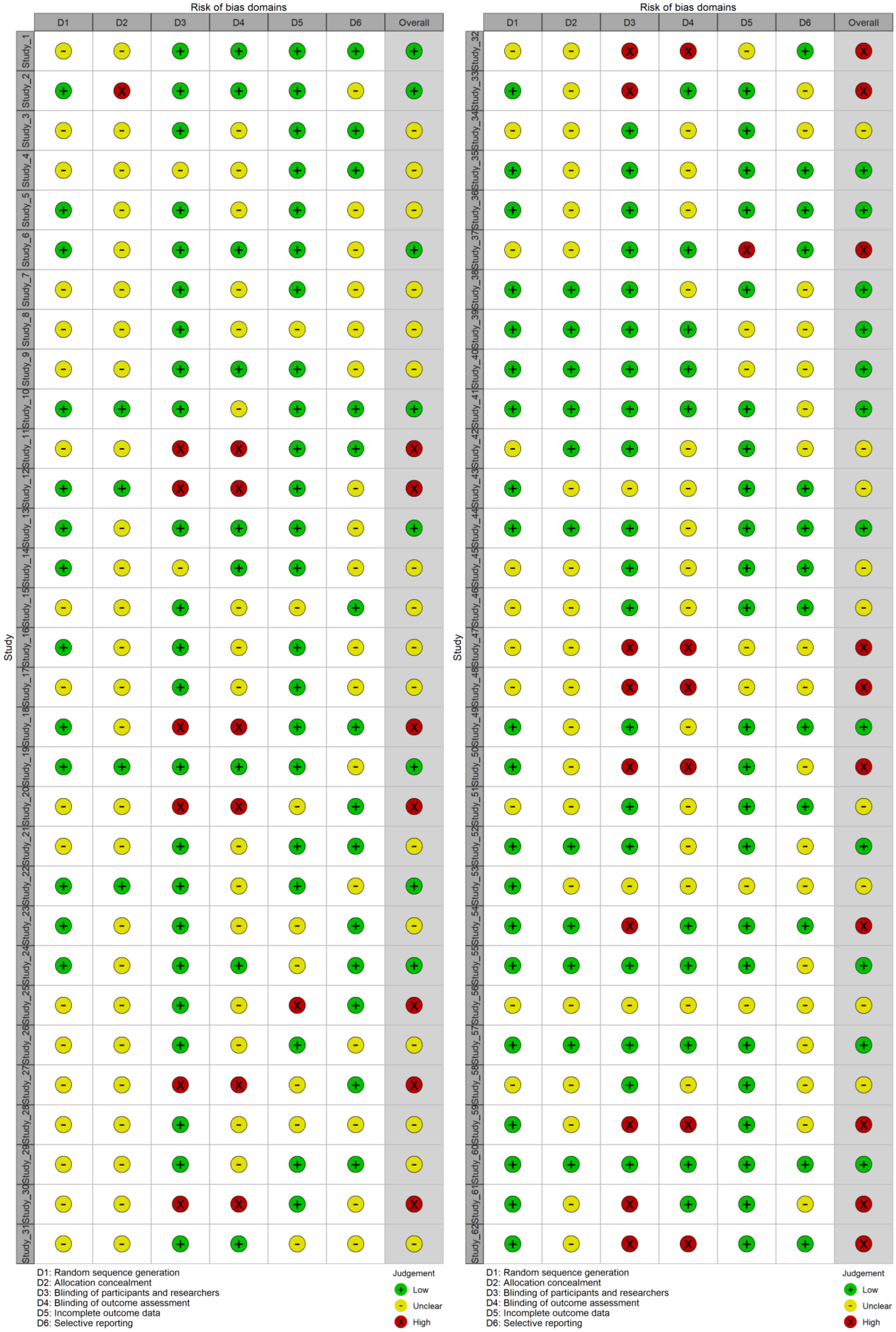

Supplementary table 4. GRADE profile for significant results from network meta-analyses

Outcome	Treatment	Comparator	Direct Evidence		Indirect Evidence		Combined Evidence	
			OR (95% CI)	Quality	OR (95% CI)	Quality	OR (95% CI)	Quality
Muscle Symptoms	Rosuvastatin	Control	1.08 (1.00, 1.16)	Moderate [b]	1.53 (0.99, 2.35)	Very Low [a b e]	1.09 (1.01, 1.16)	Moderate *
Liver Dysfunction	Atorvastatin	Control	1.30 (0.98, 1.72)	Moderate [b]	2.98 (1.30, 6.83)	Very Low [a b e]	1.41 (1.08, 1.85)	Moderate *
Liver Dysfunction	Lovastatin	Control	1.81 (1.24, 2.67)	High	\	\	1.81 (1.23, 2.66)	High
Liver Dysfunction	Lovastatin	Fluvastatin	\	\	2.57 (1.11, 5.93)	Low [b e]	2.57 (1.11, 5.93)	Low
Liver Dysfunction	Lovastatin	Pravastatin	1.01 (0.02, 51.64)	Very Low [a d e]	1.83 (1.09, 3.08)	Low [b e]	1.81 (1.08, 3.03)	Low *
Renal Insufficiency	Rosuvastatin	Control	1.13 (1.00, 1.28)	Moderate [c]	\	\	1.13 (1.00, 1.28)	Moderate
Diabetes	Rosuvastatin	Control	1.14 (1.00, 1.30)	High	\	\	1.14 (1.00, 1.30)	High
Diabetes	Atorvastatin	Pitavastatin	1.26 (0.34, 4.75)	Low [a e]	1.50 (1.08, 2.09)	Low [a b]	1.49 (1.08, 2.05)	Low
Diabetes	Rosuvastatin	Pitavastatin	\	\	1.50 (1.16, 1.94)	Low [a b]	1.50 (1.16, 1.94)	Low
Eye Conditions	Rosuvastatin	Control	1.26 (1.04, 1.52)	High	\	\	1.26 (1.04, 1.52)	High

OR: odds ratio, CI: confidence interval

a. Overall risk of bias due to more than 3 studies or more than half of the included studies presenting high risk of bias.

b. Inconsistency due to different patient groups of specific conditions included in individual studies.

c. Inconsistency due to different specific conditions reported in individual studies for the outcome.

d. Indirectness due to included participants not representative of the targeted study population.

e. Imprecision due to small sample size for direct comparison or for certain treatment comparisons that were involved in the indirect evidence.

* Combined evidence quality is determined by the higher level when the quality of direct and indirect evidence is not equal.

Supplementary figure 2. Forest plots of pair-wise meta-analyses

A Self-reported Muscle Symptoms

Study	Statins Group Events	Statins Group Total	Control Group Events	Control Group Total	OR [95% CI]	Weight
EXCEL, 1991	512	6582	125	1663	1.04 [0.85; 1.27]	7.9%
PMSG-D, 1994	2	167	3	158	0.63 [0.10; 3.80]	0.1%
Jacobsen et al, 1995	15	182	4	63	1.32 [0.42; 4.15]	0.2%
KAPS, 1995	51	224	45	223	1.17 [0.74; 1.83]	1.5%
WOSCOPS, 1995	117	3302	121	3293	0.96 [0.74; 1.25]	5.0%
AFCAPS/TexCAPS, 1998	11	3304	9	3301	1.22 [0.51; 2.95]	0.4%
Bak et al, 1998	2	106	1	109	2.08 [0.19; 23.25]	0.0%
ALERT, 2003	526	1045	531	1049	0.99 [0.83; 1.17]	11.3%
ESG-L, 2003	17	412	2	136	2.88 [0.66; 12.64]	0.1%
ESG-P, 2003	0	409	1	129	0.10 [0.00; 2.58]	0.1%
CARDS, 2004	61	1428	72	1410	0.83 [0.58; 1.18]	3.0%
COMETS, 2005	9	317	2	79	1.12 [0.24; 5.31]	0.1%
Bone et al, 2007	53	485	8	119	1.70 [0.79; 3.68]	0.5%
Lewis et al, 2007	2	163	0	163	5.06 [0.24; 106.26]	0.0%
METEOR, 2007	128	700	55	281	0.92 [0.65; 1.31]	2.8%
JUPITER, 2008	1421	8901	1375	8901	1.04 [0.96; 1.13]	49.6%
STOMP, 2013	19	203	10	217	2.14 [0.97; 4.71]	0.4%
HOPE-3, 2016	367	6361	296	6344	1.25 [1.07; 1.46]	12.0%
Liu et al, 2018	2	135	1	45	0.66 [0.06; 7.47]	0.1%
BALANCE, 2019	12	96	7	97	1.84 [0.69; 4.89]	0.3%
TRACE RA, 2019	132	1504	117	1498	1.14 [0.88; 1.47]	4.6%
**Fixed effect model**	**3459**	**36026**	**2785**	**29278**	**1.06 [1.01; 1.13]**	**100.0%**

Heterogeneity: $I^2 = 1\%$ [0%; 47%], $\tau^2 = 0.0001$, $p = 0.45$
Test for overall effect: $z = 2.18$ ($p = 0.03$)

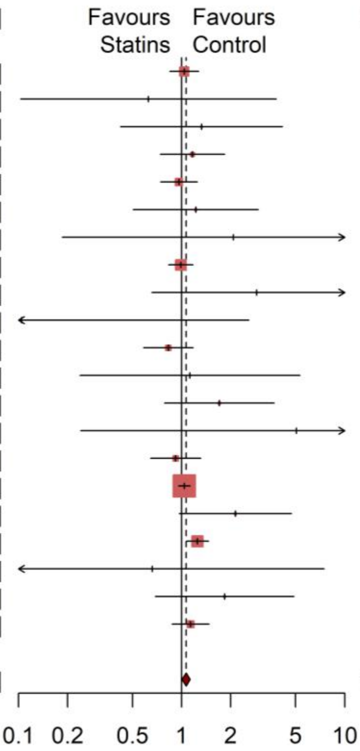

B Clinically-confirmed Muscle Disorders

Study	Statins Group Events	Statins Group Total	Control Group Events	Control Group Total	OR [95% CI]	Weight
EXCEL, 1991	17	6582	7	1663	0.61 [0.25; 1.48]	16.0%
PMSG-D, 1994	0	167	0	158	0.95 [0.02; 47.98]	0.7%
Jacobsen et al, 1995	1	182	0	63	1.05 [0.04; 26.09]	1.1%
KAPS, 1995	0	224	0	223	1.00 [0.02; 50.39]	0.7%
WOSCOPS, 1995	3	3302	1	3293	2.99 [0.31; 28.79]	1.4%
AFCAPS/TexCAPS, 1998	22	3243	23	3248	0.96 [0.53; 1.72]	32.8%
Gentile et al, 2000	0	323	0	86	0.27 [0.01; 13.57]	1.1%
ALERT, 2003	4	1045	2	1049	2.01 [0.37; 11.01]	2.9%
ASCOT-LLA, 2003	1	5168	0	5137	2.98 [0.12; 73.23]	0.7%
ESG-L, 2003	0	412	0	136	0.33 [0.01; 16.76]	1.1%
ESG-P, 2003	2	409	0	129	1.59 [0.08; 33.31]	1.1%
CARDS, 2004	1	1428	5	1410	0.20 [0.02; 1.69]	7.2%
ESG-S, 2004	2	1231	1	297	0.48 [0.04; 5.33]	2.3%
COMETS, 2005	2	317	0	79	1.26 [0.06; 26.51]	1.1%
HYRIM, 2005	0	283	1	285	0.33 [0.01; 8.25]	2.1%
MEGA, 2006	0	3866	0	3966	1.03 [0.02; 51.71]	0.7%
Bone et al, 2007	0	485	0	119	0.25 [0.00; 12.47]	1.2%
Lewis et al, 2007	0	163	1	163	0.33 [0.01; 8.19]	2.2%
METEOR, 2007	1	700	2	281	0.20 [0.02; 2.21]	4.1%
JUPITER, 2008	11	8901	9	8901	1.22 [0.51; 2.95]	12.9%
RCASS, 2009	0	113	0	114	1.01 [0.02; 51.28]	0.7%
ASTRONOMER, 2010	1	134	2	135	0.50 [0.04; 5.58]	2.8%
STOMP, 2013	0	203	0	217	1.07 [0.02; 54.12]	0.7%
HOPE-3, 2016	2	6361	1	6344	1.99 [0.18; 22.01]	1.4%
TRACE RA, 2019	0	1504	0	1498	1.00 [0.02; 50.23]	0.7%
**Fixed effect model**	**70**	**46746**	**55**	**38994**	**0.88 [0.62; 1.24]**	**100.0%**

Heterogeneity: $I^2 = 0\%$ [0%; 0%], $\tau^2 = 0$, $p = 0.99$
Test for overall effect: $z = -0.74$ ($p = 0.46$)

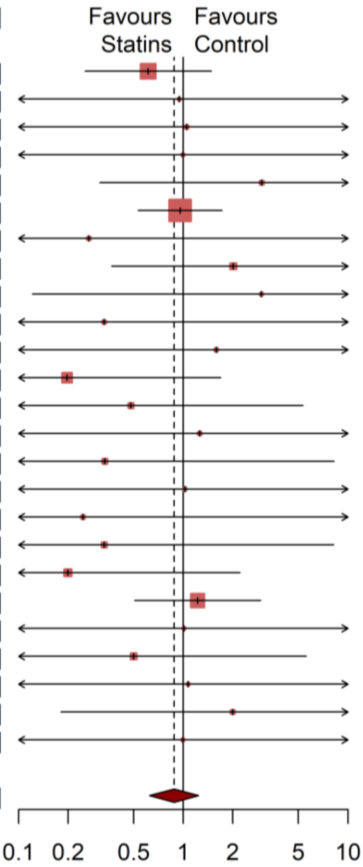

C Liver Dysfunction

Study	Statins Group Events	Statins Group Total	Control Group Events	Control Group Total	OR [95% CI]	Weight
EXCEL, 1991	140	6582	17	1663	2.10 [1.27; 3.49]	11.7%
ACAPS, 1994	6	460	6	459	1.00 [0.32; 3.12]	2.6%
PMSG-D, 1994	1	167	0	158	2.86 [0.12; 70.62]	0.2%
Jacobsen et al, 1995	0	182	0	63	0.35 [0.01; 17.72]	0.3%
KAPS, 1995	4	224	3	223	1.33 [0.29; 6.03]	1.3%
WOSCOPS, 1995	42	3302	32	3293	1.31 [0.83; 2.08]	13.9%
AFCAPS/TexCAPS, 1998	18	3242	11	3248	1.64 [0.77; 3.48]	4.8%
Gentile et al, 2000	0	323	0	86	0.27 [0.01; 13.57]	0.3%
ALERT, 2003	12	1045	17	1049	0.71 [0.34; 1.48]	7.4%
ESG-L, 2003	1	412	0	136	1.00 [0.04; 24.57]	0.3%
ESG-P, 2003	3	409	0	129	2.23 [0.11; 43.46]	0.3%
CARDS, 2004	23	1428	18	1410	1.27 [0.68; 2.36]	7.9%
ESG-S, 2004	16	1215	2	294	1.95 [0.45; 8.52]	1.4%
COMETS, 2005	1	317	0	79	0.75 [0.03; 18.67]	0.4%
Bone et al, 2007	2	485	0	119	1.24 [0.06; 25.91]	0.4%
Lewis et al, 2007	12	160	20	160	0.57 [0.27; 1.20]	8.1%
METEOR, 2007	4	700	1	281	1.61 [0.18; 14.46]	0.6%
JUPITER, 2008	23	8901	17	8901	1.35 [0.72; 2.54]	7.5%
RCASS, 2009	0	113	0	114	1.01 [0.02; 51.28]	0.2%
ASTRONOMER, 2010	8	134	4	135	2.08 [0.61; 7.08]	1.7%
TRACE RA, 2019	90	1504	69	1498	1.32 [0.96; 1.82]	28.6%
**Fixed effect model**	**406**	**31305**	**217**	**23498**	**1.33 [1.12; 1.58]**	**100.0%**

Heterogeneity: I^2 = 0% [0%; 23%], τ^2 = 0, p = 0.84
Test for overall effect: z = 3.20 (p < 0.01)

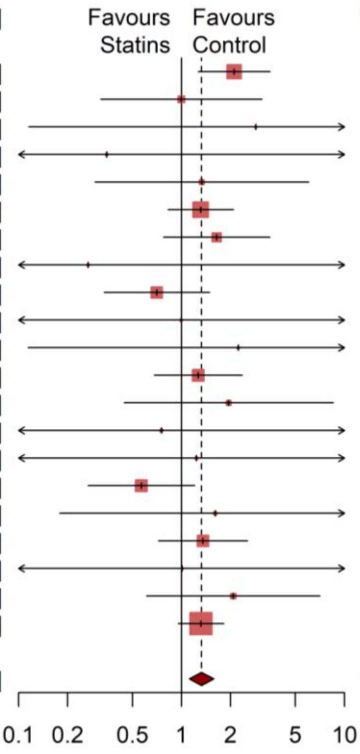

D Renal Insufficiency

Study	Statins Group Events	Statins Group Total	Control Group Events	Control Group Total	OR [95% CI]	Weight
ASCOT-LLA, 2003	31	5168	24	5137	1.29 [0.75; 2.19]	4.8%
ESG-S, 2004	2	1231	0	297	1.21 [0.06; 25.27]	0.2%
COMETS, 2005	0	317	0	79	0.25 [0.00; 12.72]	0.2%
METEOR, 2007	6	700	3	281	0.80 [0.20; 3.23]	0.9%
JUPITER, 2008	535	8901	480	8901	1.12 [0.99; 1.27]	91.3%
ASTRONOMER, 2010	3	134	4	135	0.75 [0.16; 3.42]	0.8%
Liu et al, 2018	4	135	0	45	3.11 [0.16; 58.97]	0.1%
METEOR-China, 2019	16	272	9	268	1.80 [0.78; 4.14]	1.7%
**Fixed effect model**	**597**	**16858**	**520**	**15143**	**1.14 [1.01; 1.28]**	**100.0%**

Heterogeneity: I^2 = 0% [0%; 23%], τ^2 = 0, p = 0.89
Test for overall effect: z = 2.09 (p = 0.04)

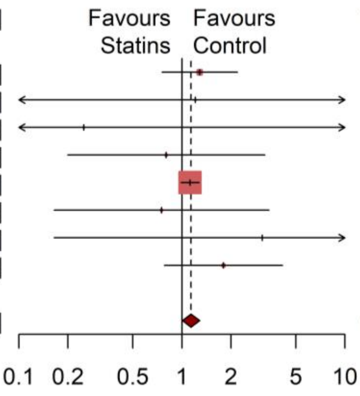

E Diabetes

Study	Statins Group Events	Statins Group Total	Control Group Events	Control Group Total	OR [95% CI]	Weight
WOSCOPS, 1995	75	2999	93	2975	0.79 [0.58; 1.08]	8.7%
AFCAPS/TexCAPS, 1998	72	3094	74	3117	0.98 [0.71; 1.36]	6.9%
ASCOT-LLA, 2003	154	3910	134	3863	1.14 [0.90; 1.44]	12.4%
MEGA, 2006	172	3013	164	3073	1.07 [0.86; 1.34]	14.7%
JUPITER, 2008	270	8901	216	8901	1.26 [1.05; 1.51]	20.1%
ASTRONOMER, 2010	1	134	0	135	3.04 [0.12; 75.41]	0.0%
J-PREDICT, 2014	213	634	254	635	0.76 [0.60; 0.95]	16.2%
HOPE-3, 2016	232	6361	226	6344	1.02 [0.85; 1.23]	20.9%
METEOR-China, 2019	1	272	0	268	2.97 [0.12; 73.15]	0.0%
**Random effects model**	**1190**	**29318**	**1161**	**29311**	**1.01 [0.88; 1.16]**	**--**

Heterogeneity: I^2 = 50% [0%; 77%], τ^2 = 0.0183, p = 0.04
Test for overall effect: z = 0.15 (p = 0.88)

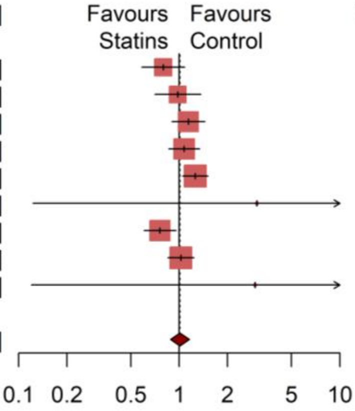

F Eye Conditions

Study	Statins Group Events	Statins Group Total	Control Group Events	Control Group Total	OR [95% CI]	Weight
EXCEL, 1991	41	6582	8	1663	1.30 [0.61; 2.77]	5.5%
PMSG-D, 1994	23	151	23	137	0.89 [0.47; 1.67]	8.9%
ESG-L, 2003	2	412	1	136	0.66 [0.06; 7.32]	0.7%
HOPE-3, 2016	241	6361	194	6344	1.25 [1.03; 1.51]	81.4%
METEOR-China, 2019	6	272	3	268	1.99 [0.49; 8.05]	1.3%
TRACE RA, 2019	8	1504	5	1498	1.60 [0.52; 4.89]	2.2%
**Fixed effect model**	**321**	**15282**	**234**	**10046**	**1.23 [1.04; 1.47]**	**100.0%**

Heterogeneity: I^2 = 0% [0%; 36%], τ^2 = 0, p = 0.85
Test for overall effect: z = 2.35 (p = 0.02)

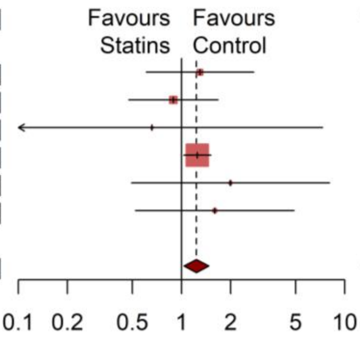

G Myocardial Infarction

Study	Statins Group Events	Statins Group Total	Control Group Events	Control Group Total	OR [95% CI]	Weight
EXCEL, 1991	47	6582	18	1663	0.66 [0.38; 1.13]	2.2%
ACAPS, 1994	5	460	5	459	1.00 [0.29; 3.47]	0.4%
PMSG-D, 1994	0	167	1	158	0.31 [0.01; 7.75]	0.1%
KAPS, 1995	3	224	8	223	0.36 [0.10; 1.39]	0.6%
WOSCOPS, 1995	143	3302	204	3293	0.69 [0.55; 0.85]	15.3%
CAIUS, 1996	2	151	2	154	1.02 [0.14; 7.34]	0.2%
AFCAPS/TexCAPS, 1998	57	3304	95	3301	0.59 [0.43; 0.83]	7.3%
ALLHAT-LLT, 2002	380	5170	421	5185	0.90 [0.78; 1.04]	30.6%
ALERT, 2003	70	1050	104	1052	0.65 [0.47; 0.89]	7.6%
ASCOT-LLA, 2003	100	5168	154	5137	0.64 [0.49; 0.82]	11.9%
Mohler et al, 2003	6	240	3	114	0.95 [0.23; 3.86]	0.3%
CARDS, 2004	33	1428	61	1410	0.52 [0.34; 0.80]	4.7%
PHYLLIS, 2004	1	254	3	254	0.33 [0.03; 3.20]	0.2%
PREVEND-IT, 2004	8	433	15	431	0.52 [0.22; 1.24]	1.2%
ASPEN-Primary, 2006	28	959	34	946	0.81 [0.49; 1.34]	2.6%
MEGA, 2006	17	3866	33	3966	0.53 [0.29; 0.95]	2.5%
Lewis et al, 2007	0	163	2	163	0.20 [0.01; 4.15]	0.2%
JUPITER, 2008	31	8901	68	8901	0.45 [0.30; 0.69]	5.3%
ASTRONOMER, 2010	0	134	3	135	0.14 [0.01; 2.75]	0.3%
HOPE-3, 2016	45	6361	69	6344	0.65 [0.44; 0.94]	5.4%
METEOR-China, 2019	0	272	2	268	0.20 [0.01; 4.09]	0.2%
TRACE RA, 2019	20	1504	11	1498	1.82 [0.87; 3.82]	0.9%
**Fixed effect model**	**996**	**50093**	**1316**	**45055**	**0.72 [0.66; 0.78]**	**100.0%**

Heterogeneity: I^2 = 33% [0%; 60%], τ^2 = 0.0240, p = 0.07
Test for overall effect: z = -7.74 (p < 0.01)

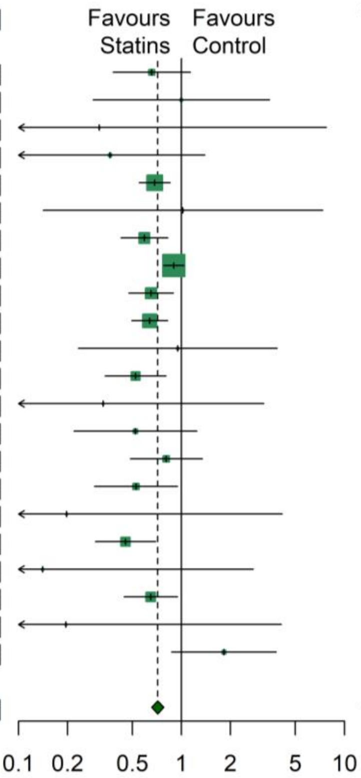

H Stroke

Study	Statins Group Events	Statins Group Total	Control Group Events	Control Group Total	OR [95% CI]	Weight
ACAPS, 1994	0	460	5	459	0.09 [0.00; 1.63]	0.7%
KAPS, 1995	2	224	4	223	0.49 [0.09; 2.72]	0.5%
WOSCOPS, 1995	46	3302	51	3293	0.90 [0.60; 1.34]	6.6%
ALLHAT-LLT, 2002	209	5170	231	5185	0.90 [0.75; 1.09]	28.8%
ALERT, 2003	74	1050	63	1052	1.19 [0.84; 1.68]	7.6%
ASCOT-LLA, 2003	89	5168	121	5137	0.73 [0.55; 0.96]	15.5%
Mohler et al, 2003	2	240	0	114	2.40 [0.11; 50.41]	0.1%
CARDS, 2004	21	1428	39	1410	0.52 [0.31; 0.90]	5.0%
Muldoon et al, 2004	1	206	0	102	1.50 [0.06; 37.05]	0.1%
PHYLLIS, 2004	1	254	0	254	3.01 [0.12; 74.28]	0.1%
ASPEN-Primary, 2006	27	959	29	946	0.92 [0.54; 1.56]	3.7%
MEGA, 2006	50	3866	62	3966	0.83 [0.57; 1.20]	7.9%
JUPITER, 2008	33	8901	64	8901	0.51 [0.34; 0.78]	8.3%
RCASS, 2009	3	113	5	114	0.59 [0.14; 2.55]	0.6%
ASTRONOMER, 2010	0	134	1	135	0.33 [0.01; 8.26]	0.2%
HOPE-3, 2016	70	6361	99	6344	0.70 [0.52; 0.96]	12.8%
TRACE RA, 2019	6	1504	12	1498	0.50 [0.19; 1.33]	1.6%
**Fixed effect model**	**634**	**39340**	**786**	**39133**	**0.80 [0.72; 0.89]**	**100.0%**

Heterogeneity: $I^2 = 20\%$ [0%; 55%], $\tau^2 = 0.0146$, $p = 0.22$
Test for overall effect: $z = -4.11$ ($p < 0.01$)

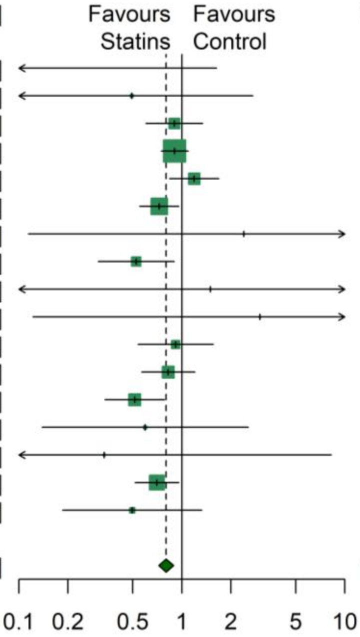

I Death from CVD

Study	Statins Group Events	Statins Group Total	Control Group Events	Control Group Total	OR [95% CI]	Weight
EXCEL, 1991	21	6582	2	1663	2.66 [0.62; 11.35]	0.3%
ACAPS, 1994	0	460	6	459	0.08 [0.00; 1.35]	0.7%
KAPS, 1995	2	224	3	223	0.66 [0.11; 3.99]	0.3%
WOSCOPS, 1995	50	3302	73	3293	0.68 [0.47; 0.98]	7.6%
AFCAPS/TexCAPS, 1998	17	3304	25	3301	0.68 [0.37; 1.26]	2.6%
ALLHAT-LLT, 2002	295	5170	300	5185	0.99 [0.84; 1.16]	29.6%
ALERT, 2003	66	1050	73	1052	0.90 [0.64; 1.27]	7.2%
ASCOT-LLA, 2003	74	5168	82	5137	0.90 [0.65; 1.23]	8.5%
Mohler et al, 2003	3	240	1	114	1.43 [0.15; 13.90]	0.1%
CARDS, 2004	25	1428	37	1410	0.66 [0.40; 1.10]	3.8%
PHYLLIS, 2004	1	254	0	254	3.01 [0.12; 74.28]	0.1%
PREVEND-IT, 2004	4	433	4	431	1.00 [0.25; 4.01]	0.4%
COMETS, 2005	2	317	0	79	1.26 [0.06; 26.51]	0.1%
ASPEN-Primary, 2006	24	959	19	946	1.25 [0.68; 2.30]	2.0%
MEGA, 2006	11	3866	18	3966	0.63 [0.30; 1.33]	1.9%
Bone et al, 2007	0	485	0	119	0.25 [0.00; 12.47]	0.1%
Lewis et al, 2007	0	163	1	163	0.33 [0.01; 8.19]	0.2%
METEOR, 2007	0	700	0	281	0.40 [0.01; 20.30]	0.1%
JUPITER, 2008	83	8901	157	8901	0.52 [0.40; 0.69]	16.3%
ASTRONOMER, 2010	2	134	5	135	0.39 [0.08; 2.07]	0.5%
HOPE-3, 2016	154	6361	171	6344	0.90 [0.72; 1.12]	17.5%
TRACE RA, 2019	2	1504	2	1498	1.00 [0.14; 7.08]	0.2%
**Fixed effect model**	**836**	**51005**	**979**	**44954**	**0.83 [0.76; 0.91]**	**100.0%**

Heterogeneity: $I^2 = 27\%$ [0%; 57%], $\tau^2 = 0.0224$, $p = 0.12$
Test for overall effect: $z = -3.86$ ($p < 0.01$)

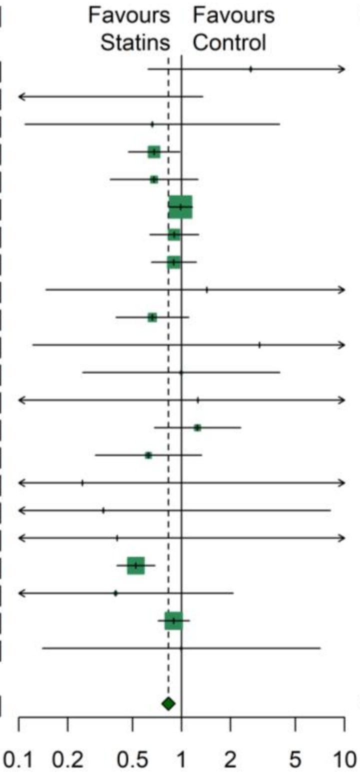

Supplementary figure 3. Leave-one-out influence analyses for pair-wise meta-analyses

A Self-reported Muscle Symptoms

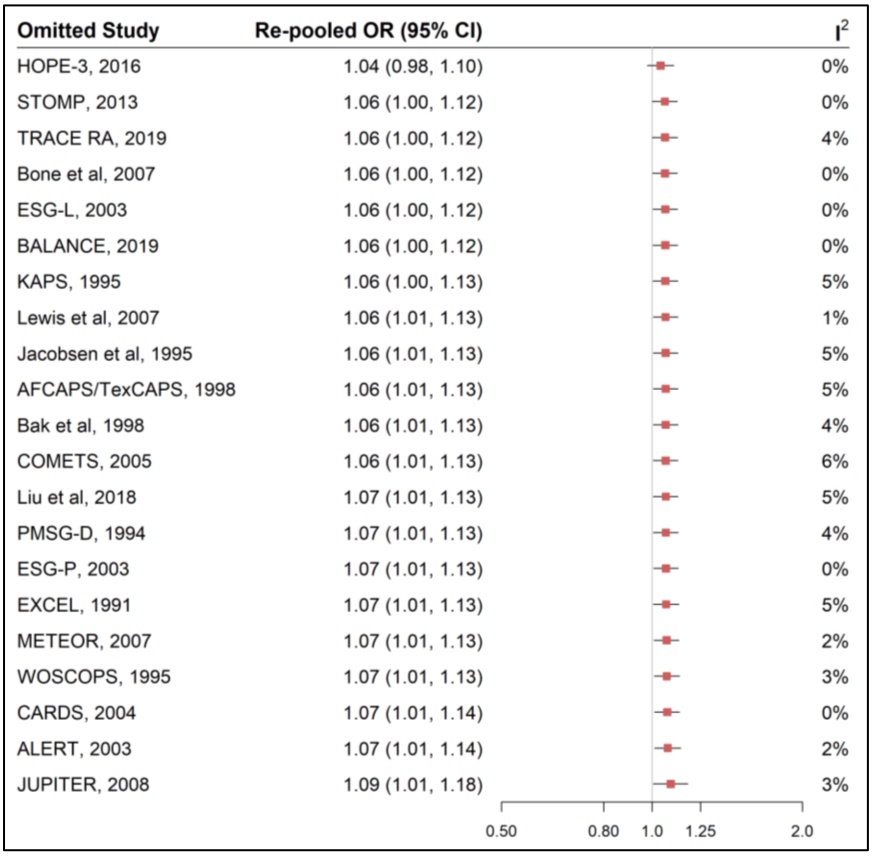

B Clinically-confirmed Muscle Disorders

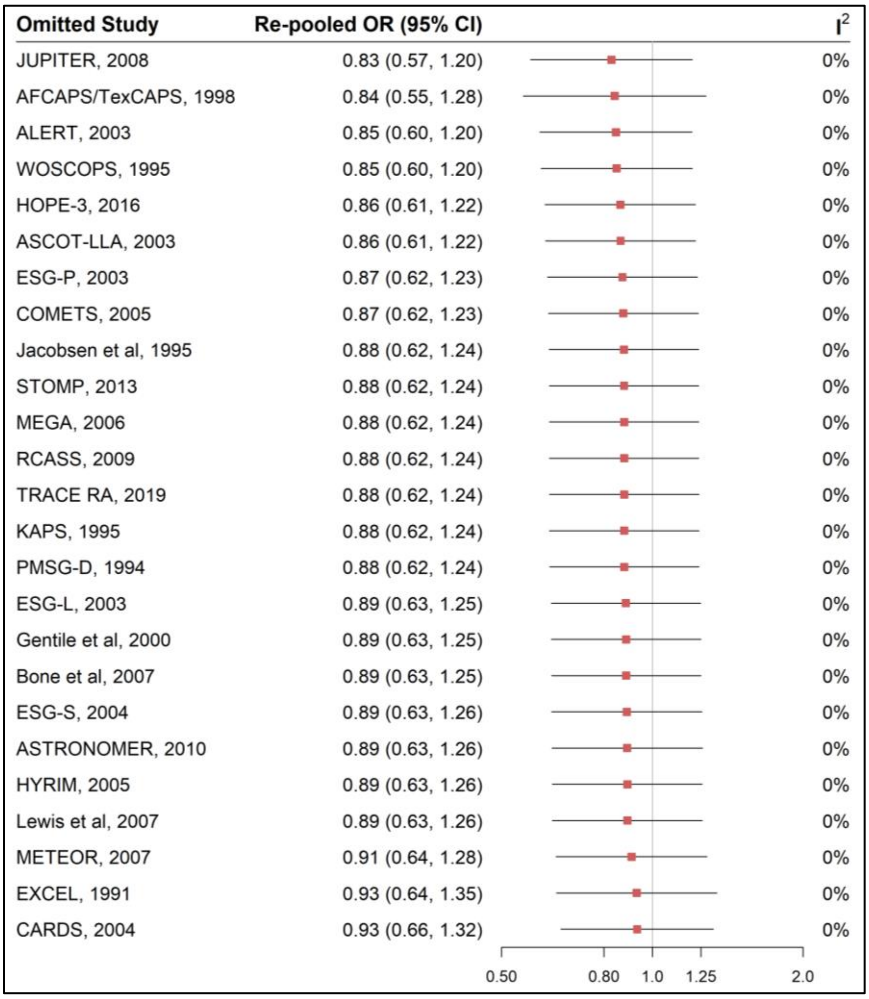

C Liver Dysfunction

Omitted Study	Re-pooled OR (95% CI)	I^2
EXCEL, 1991	1.22 (1.02, 1.48)	0%
AFCAPS/TexCAPS, 1998	1.31 (1.10, 1.57)	0%
ASTRONOMER, 2010	1.32 (1.10, 1.57)	0%
ESG-S, 2004	1.32 (1.11, 1.57)	0%
PMSG-D, 1994	1.32 (1.11, 1.58)	0%
ESG-P, 2003	1.32 (1.11, 1.58)	0%
JUPITER, 2008	1.33 (1.11, 1.59)	0%
METEOR, 2007	1.33 (1.11, 1.58)	0%
KAPS, 1995	1.33 (1.11, 1.58)	0%
Bone et al, 2007	1.33 (1.12, 1.58)	0%
RCASS, 2009	1.33 (1.12, 1.58)	0%
ESG-L, 2003	1.33 (1.12, 1.58)	0%
COMETS, 2005	1.33 (1.12, 1.58)	0%
WOSCOPS, 1995	1.33 (1.10, 1.60)	0%
Jacobsen et al, 1995	1.33 (1.12, 1.58)	0%
Gentile et al, 2000	1.33 (1.12, 1.58)	0%
TRACE RA, 2019	1.33 (1.08, 1.64)	0%
CARDS, 2004	1.33 (1.11, 1.60)	0%
ACAPS, 1994	1.34 (1.12, 1.59)	0%
ALERT, 2003	1.38 (1.15, 1.65)	0%
Lewis et al, 2007	1.40 (1.17, 1.67)	0%

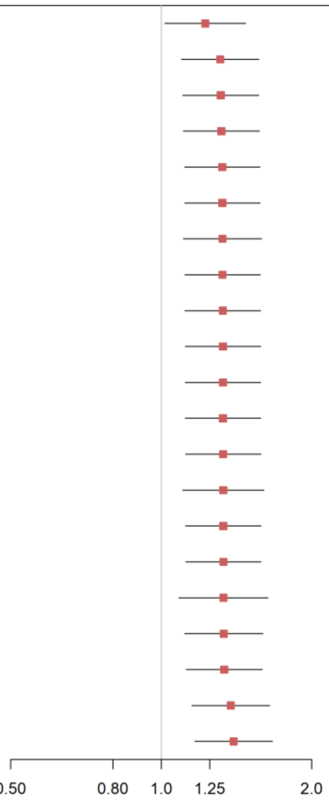

D Renal Insufficiency

Omitted Study	Re-pooled OR (95% CI)	I^2
METEOR-China, 2019	1.13 (1.00, 1.27)	0%
ASCOT-LLA, 2003	1.13 (1.00, 1.28)	0%
Liu et al, 2018	1.13 (1.01, 1.28)	0%
ESG-S, 2004	1.14 (1.01, 1.28)	0%
COMETS, 2005	1.14 (1.01, 1.29)	0%
METEOR, 2007	1.14 (1.01, 1.29)	0%
ASTRONOMER, 2010	1.14 (1.01, 1.29)	0%
JUPITER, 2008	1.30 (0.87, 1.94)	0%

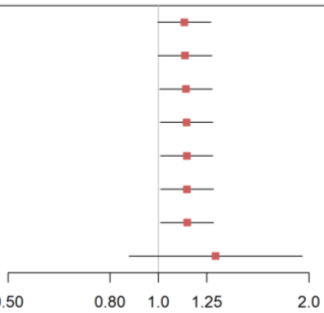

E Diabetes

Omitted Study	Re-pooled OR (95% CI)	I^2
JUPITER, 2008	0.97 (0.85, 1.09)	30%
ASCOT-LLA, 2003	0.99 (0.85, 1.15)	54%
MEGA, 2006	1.00 (0.85, 1.17)	56%
HOPE-3, 2016	1.00 (0.85, 1.19)	56%
ASTRONOMER, 2010	1.01 (0.88, 1.16)	55%
METEOR-China, 2019	1.01 (0.88, 1.16)	55%
AFCAPS/TexCAPS, 1998	1.01 (0.87, 1.18)	56%
WOSCOPS, 1995	1.04 (0.91, 1.19)	47%
J-PREDICT, 2014	1.07 (0.97, 1.19)	14%

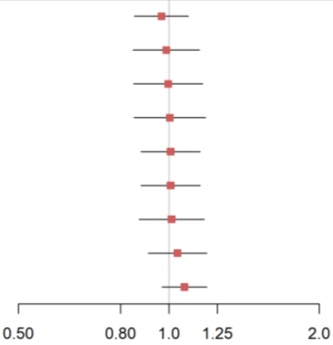

F Eye Conditions

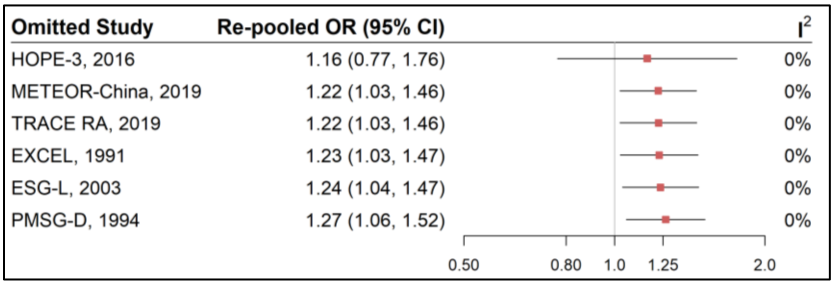

G Myocardial Infarction

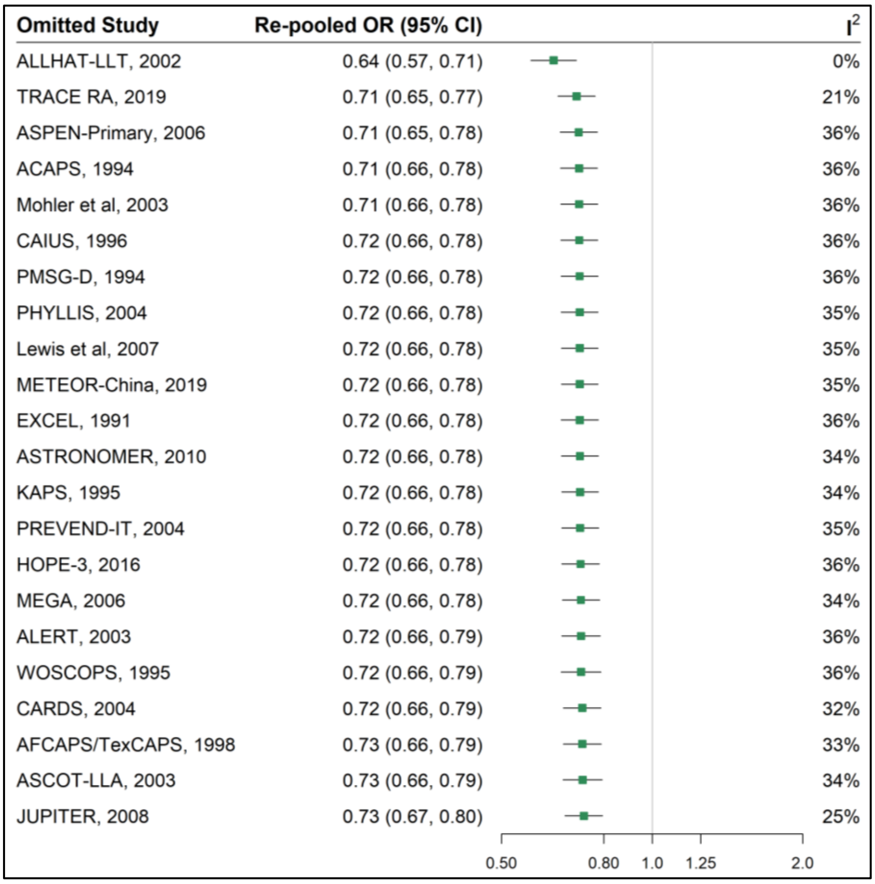

H Stroke

Omitted Study	Re-pooled OR (95% CI)	I^2
ALLHAT-LLT, 2002	0.76 (0.67, 0.86)	17%
ALERT, 2003	0.77 (0.69, 0.86)	0%
WOSCOPS, 1995	0.79 (0.71, 0.89)	24%
ASPEN-Primary, 2006	0.80 (0.71, 0.89)	25%
MEGA, 2006	0.80 (0.71, 0.89)	25%
PHYLLIS, 2004	0.80 (0.72, 0.89)	23%
Mohler et al, 2003	0.80 (0.72, 0.89)	24%
Muldoon et al, 2004	0.80 (0.72, 0.89)	25%
ASTRONOMER, 2010	0.80 (0.72, 0.89)	24%
RCASS, 2009	0.80 (0.72, 0.89)	25%
KAPS, 1995	0.80 (0.72, 0.89)	24%
TRACE RA, 2019	0.81 (0.72, 0.90)	22%
ACAPS, 1994	0.81 (0.72, 0.90)	16%
ASCOT-LLA, 2003	0.81 (0.73, 0.91)	23%
HOPE-3, 2016	0.81 (0.73, 0.91)	22%
CARDS, 2004	0.82 (0.73, 0.91)	15%
JUPITER, 2008	0.83 (0.74, 0.92)	3%

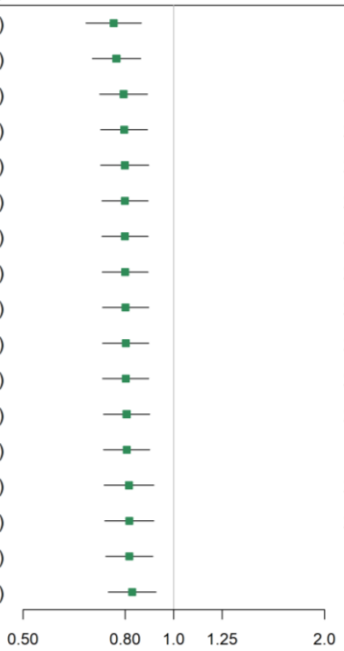

I Death from CVD

Omitted Study	Re-pooled OR (95% CI)	I^2
ALLHAT-LLT, 2002	0.77 (0.68, 0.86)	13%
HOPE-3, 2016	0.82 (0.74, 0.91)	29%
ASPEN-Primary, 2006	0.82 (0.75, 0.90)	26%
EXCEL, 1991	0.82 (0.75, 0.91)	24%
ASCOT-LLA, 2003	0.82 (0.75, 0.91)	30%
ALERT, 2003	0.83 (0.75, 0.91)	30%
PHYLLIS, 2004	0.83 (0.75, 0.91)	29%
Mohler et al, 2003	0.83 (0.76, 0.91)	30%
PREVEND-IT, 2004	0.83 (0.76, 0.91)	30%
COMETS, 2005	0.83 (0.76, 0.91)	30%
TRACE RA, 2019	0.83 (0.76, 0.91)	30%
METEOR, 2007	0.83 (0.76, 0.91)	30%
Bone et al, 2007	0.83 (0.76, 0.91)	30%
KAPS, 1995	0.83 (0.76, 0.91)	30%
Lewis et al, 2007	0.83 (0.76, 0.91)	30%
ASTRONOMER, 2010	0.83 (0.76, 0.92)	29%
MEGA, 2006	0.83 (0.76, 0.92)	29%
AFCAPS/TexCAPS, 1998	0.83 (0.76, 0.92)	29%
ACAPS, 1994	0.84 (0.76, 0.92)	23%
CARDS, 2004	0.84 (0.76, 0.92)	28%
WOSCOPS, 1995	0.84 (0.76, 0.93)	27%
JUPITER, 2008	0.89 (0.80, 0.99)	0%

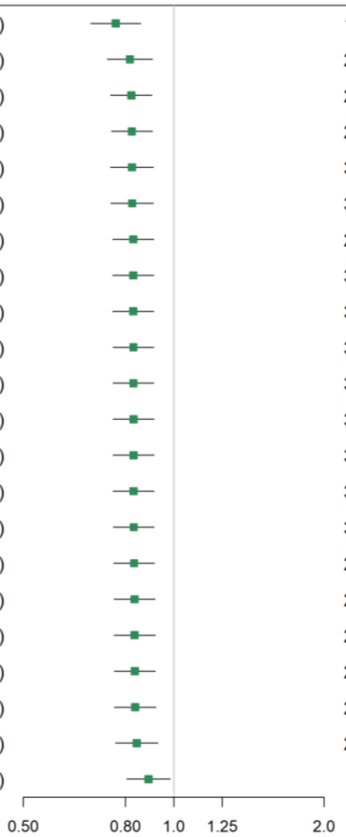

Supplementary figure 4. Funnel plots of publication bias in pair-wise meta-analyses

A Self-reported Muscle Symptoms

Test of funnel plot asymmetry: P = 0.3440

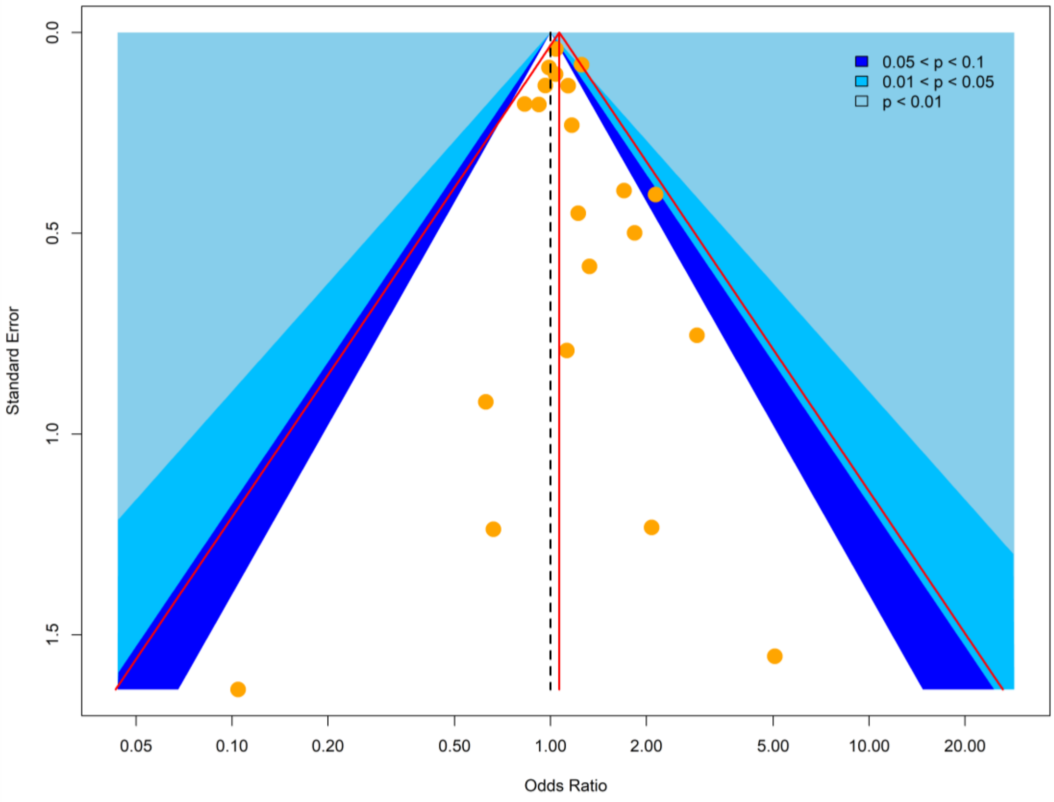

B Clinically-confirmed Muscle Disorders

Test of funnel plot asymmetry: P = 0.9921

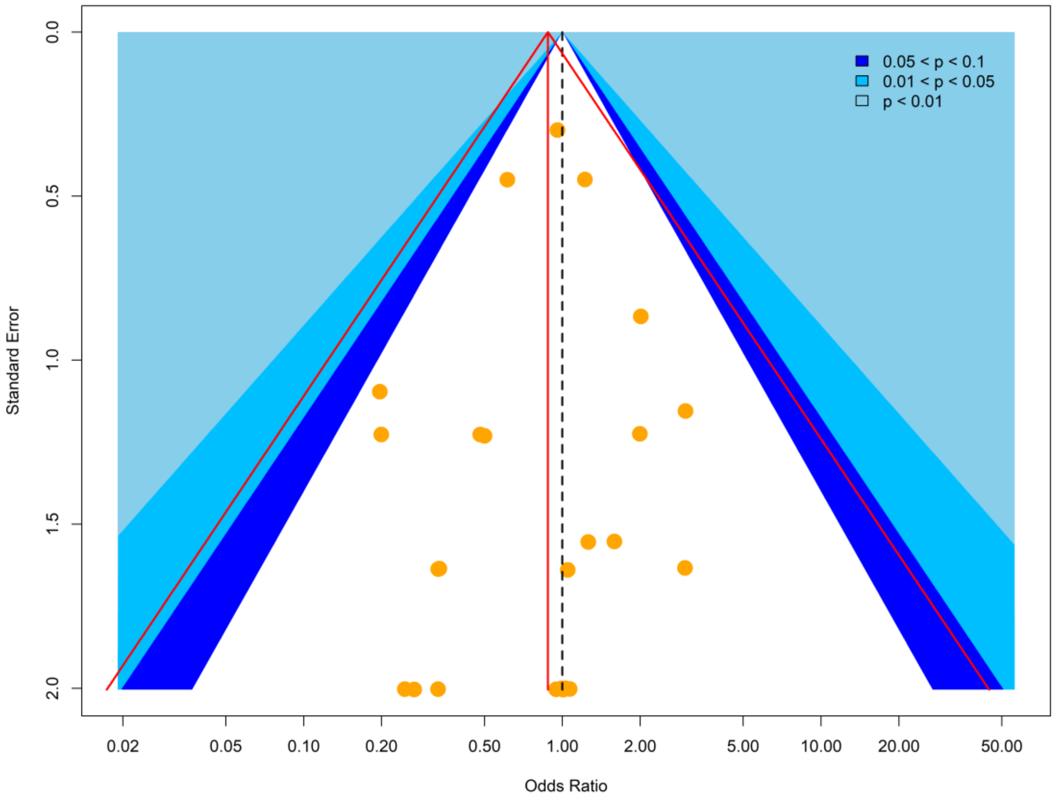

C Liver Dysfunction

Test of funnel plot asymmetry: P = 0.5029

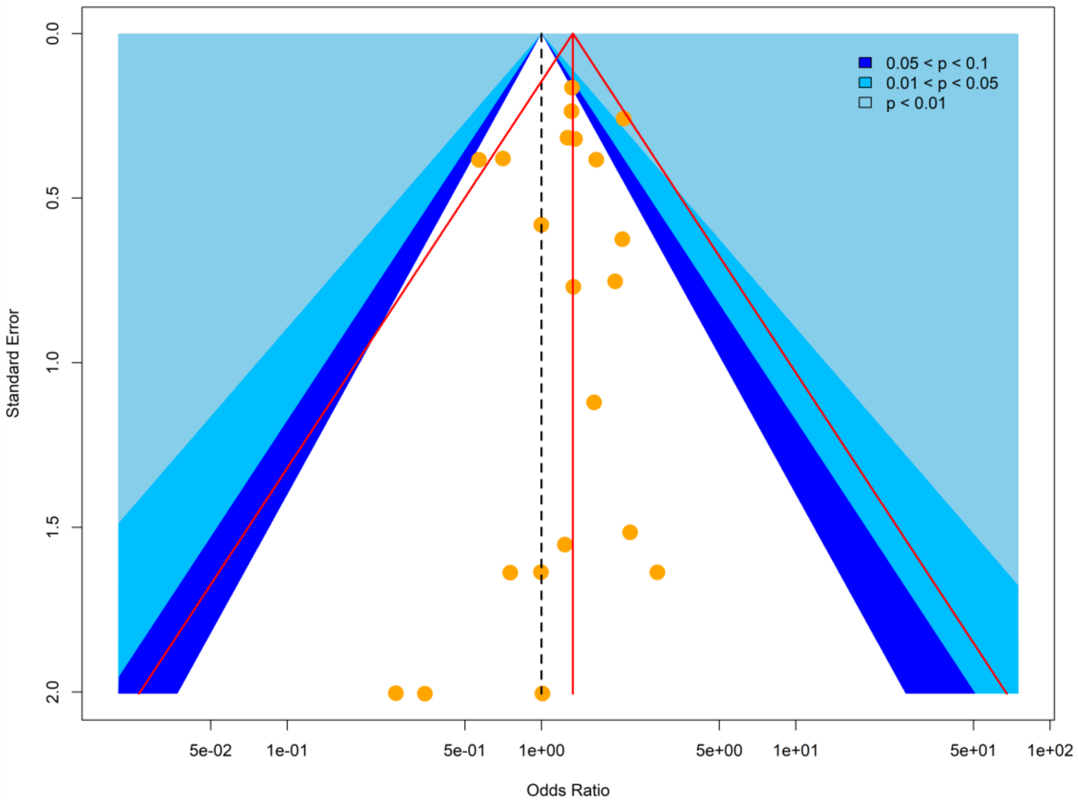

D Renal Insufficiency

Test of funnel plot asymmetry: the number of studies <10, no test was performed.

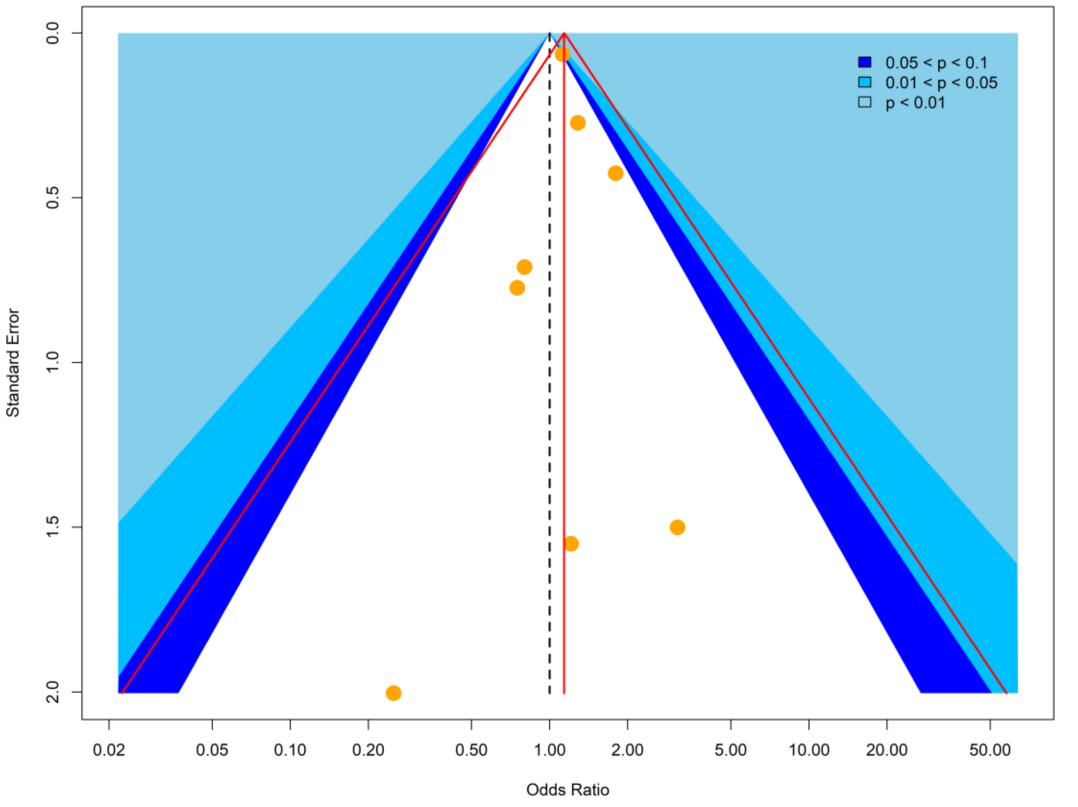

E Diabetes

Test of funnel plot asymmetry: the number of studies <10, no test was performed.

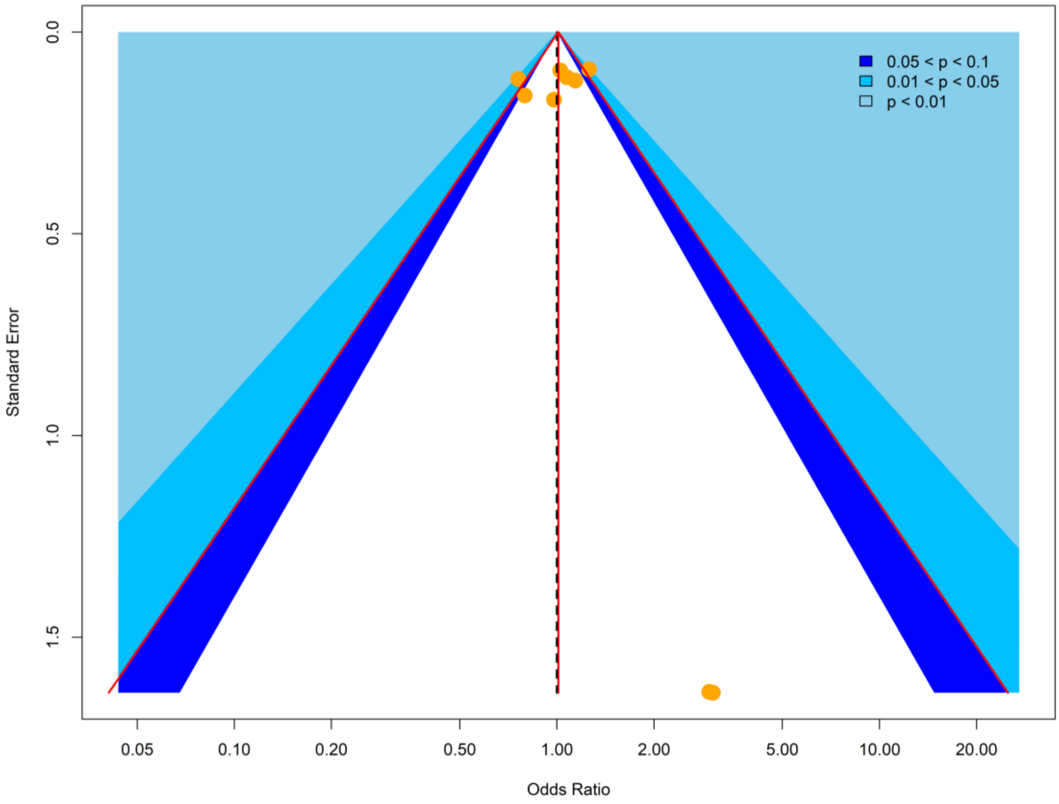

F Eye Conditions

Test of funnel plot asymmetry: the number of studies <10, no test was performed.

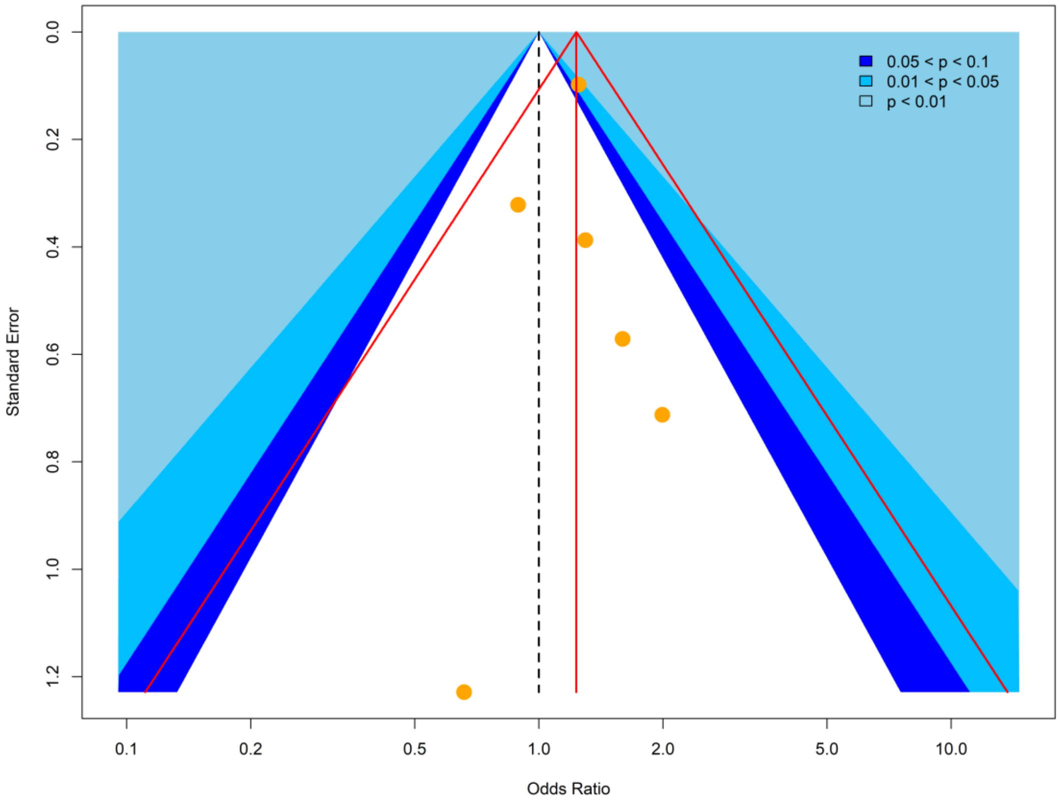

G Myocardial Infarction

Test of funnel plot asymmetry: P = 0.0360

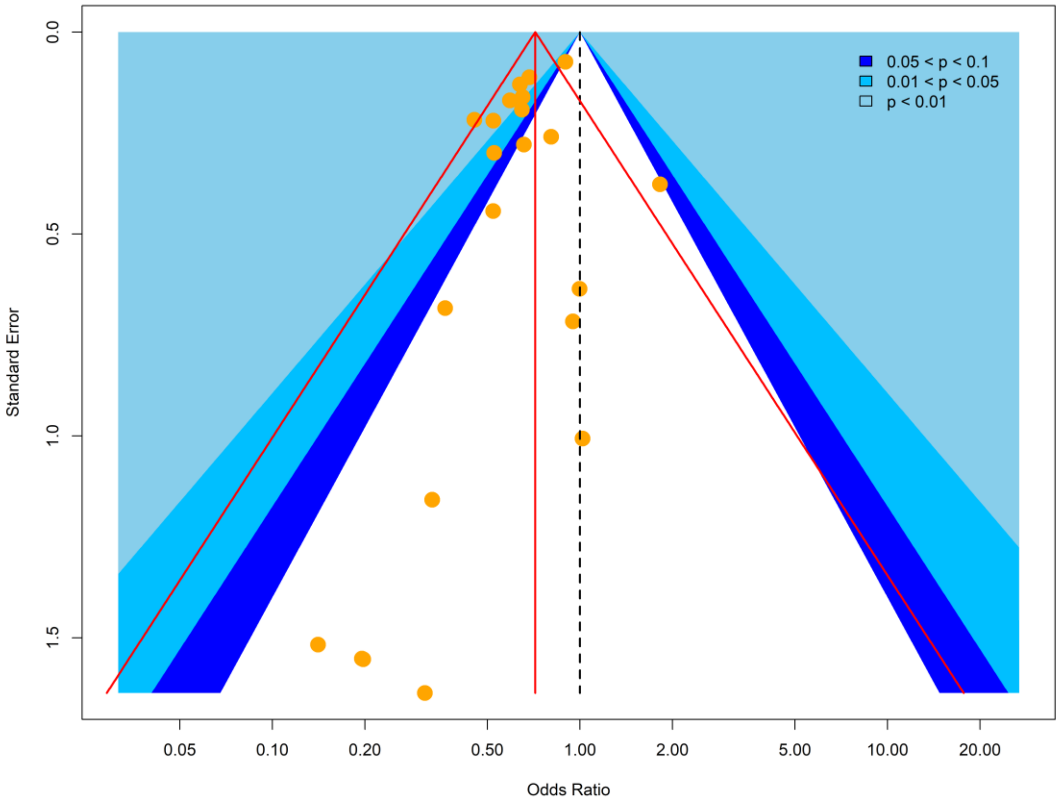

H Stroke

Test of funnel plot asymmetry: P = 0.4983

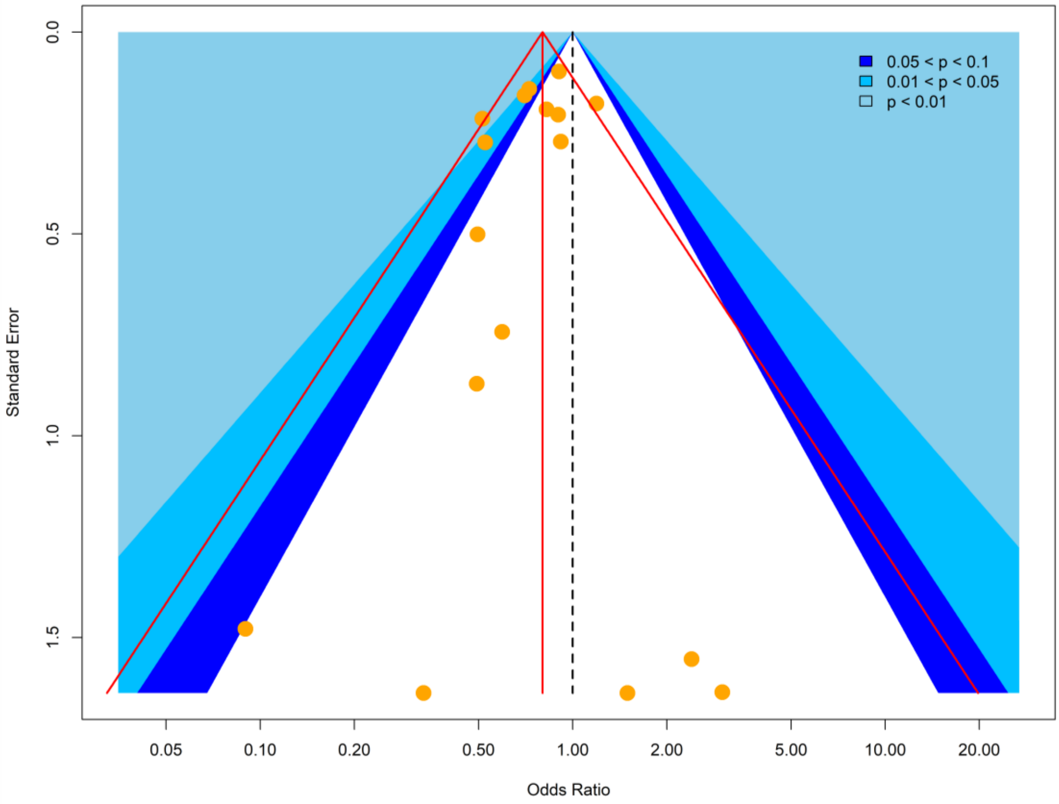

I Death from CVD

Test of funnel plot asymmetry: P = 0.6858

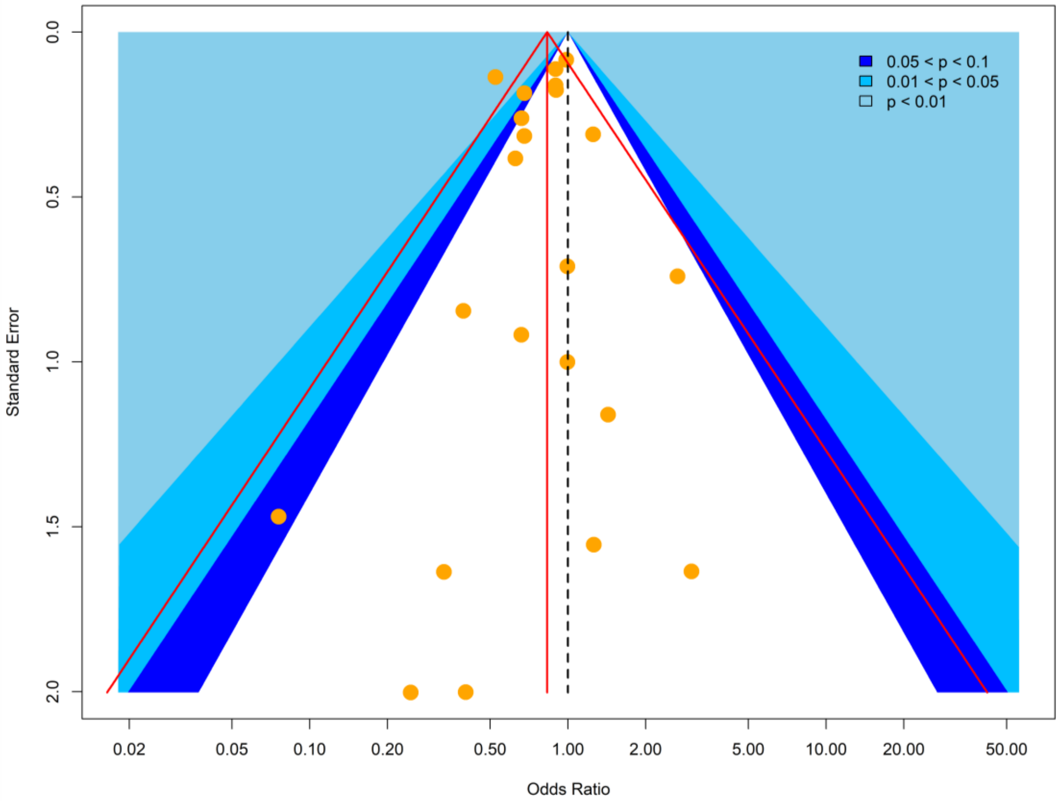

Supplementary table 5. Sensitivity analyses for pair-wise meta-analyses

Outcome	Alternative Model of Meta-analysis *	Excluding Studies with CVD Patients	Excluding Small Studies #
Muscle Symptoms	1.06 (1.00, 1.13)	1.08 (1.01, 1.16)	\
Muscle Disorders	0.88 (0.62, 1.24)	0.83 (0.55, 1.24)	\
Liver Dysfunction	1.31 (1.09, 1.56)	1.26 (1.02, 1.57)	\
Renal Insufficiency	1.14 (1.01, 1.28)	1.13 (1.00, 1.28)	\
Diabetes	1.03 (0.94, 1.12)	1.02 (0.87, 1.20)	\
Eye Conditions	1.23 (1.03, 1.47)	1.23 (1.03, 1.48)	\
Myocardial Infarction	0.67 (0.59, 0.76)	0.60 (0.50, 0.71)	0.72 (0.66, 0.78)
Stroke	0.79 (0.69, 0.90)	0.65 (0.54, 0.78)	\
Death from CVD	0.81 (0.70, 0.93)	0.70 (0.60, 0.81)	\

* Alternative model is a fixed-effect model for diabetes, for which a random-effect model has been used in the main analysis, and a random-effect model for other outcomes, for which a fixed-effect model has been used in the main analysis.

# Sensitivity analysis by excluding small studies was conducted only for myocardial infarction, of which publication bias was detected in the main analysis; 5 studies with less than 200 participants in any arm were excluded.

Supplementary figure 5. Networks of treatment comparisons in network meta-analyses

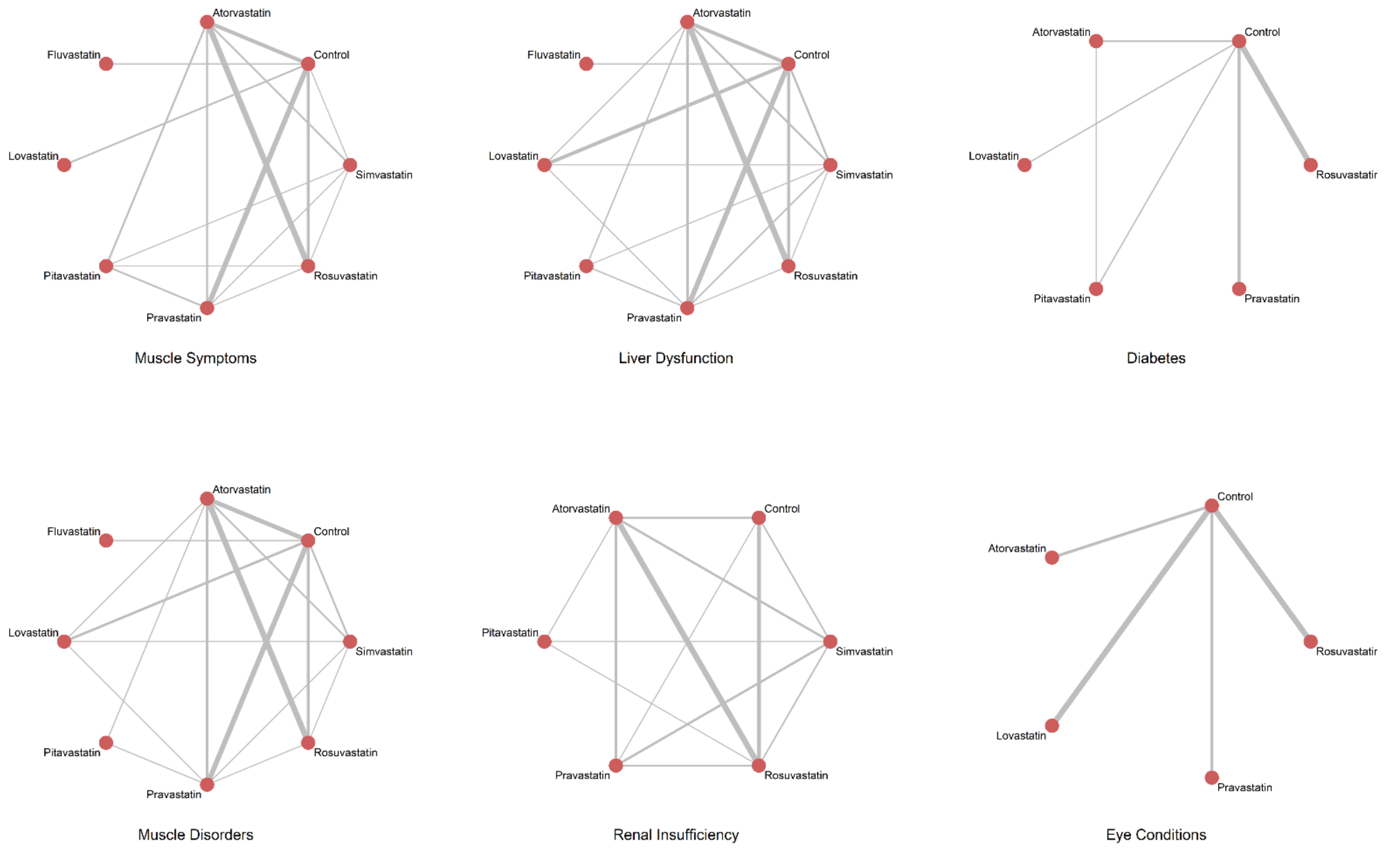

Supplementary table 6. Comparative adverse effects between different statin types from fixed-effect network meta-analyses (main analyses) *

**Muscle Symptoms**						
**Atorvastatin**	1.07 (0.84, 1.37)	0.99 (0.76, 1.28)	1.52 (0.94, 2.44)	1.07 (0.83, 1.39)	0.98 (0.82, 1.17)	1.66 (0.81, 3.39)
0.93 (0.73, 1.19)	**Fluvastatin**	0.92 (0.71, 1.20)	1.41 (0.86, 2.32)	1.00 (0.76, 1.31)	0.91 (0.76, 1.10)	1.55 (0.74, 3.24)
1.01 (0.78, 1.31)	1.09 (0.84, 1.41)	**Lovastatin**	1.53 (0.92, 2.54)	1.08 (0.82, 1.44)	0.99 (0.80, 1.22)	1.68 (0.79, 3.54)
0.66 (0.41, 1.06)	0.71 (0.43, 1.16)	0.65 (0.39, 1.08)	**Pitavastatin**	0.71 (0.45, 1.11)	0.64 (0.40, 1.03)	1.09 (0.51, 2.33)
0.93 (0.72, 1.21)	1.00 (0.77, 1.31)	0.92 (0.69, 1.23)	1.41 (0.90, 2.22)	**Pravastatin**	0.91 (0.73, 1.13)	1.55 (0.74, 3.22)
1.02 (0.86, 1.22)	1.10 (0.91, 1.32)	1.01 (0.82, 1.25)	1.55 (0.97, 2.48)	1.10 (0.88, 1.36)	**Rosuvastatin**	1.70 (0.82, 3.50)
0.60 (0.30, 1.23)	0.65 (0.31, 1.36)	0.60 (0.28, 1.26)	0.91 (0.43, 1.95)	0.65 (0.31, 1.35)	0.59 (0.29, 1.21)	**Simvastatin**
**Muscle Disorders**						
**Atorvastatin**	0.59 (0.12, 2.96)	0.92 (0.37, 2.30)	0.71 (0.11, 4.60)	0.77 (0.27, 2.24)	0.83 (0.36, 1.89)	0.85 (0.24, 2.99)
1.68 (0.34, 8.38)	**Fluvastatin**	1.55 (0.35, 6.80)	1.19 (0.11, 12.93)	1.30 (0.24, 6.95)	1.39 (0.30, 6.44)	1.42 (0.23, 8.94)
1.09 (0.43, 2.72)	0.65 (0.15, 2.84)	**Lovastatin**	0.77 (0.10, 5.63)	0.84 (0.30, 2.36)	0.90 (0.41, 1.98)	0.92 (0.26, 3.30)
1.42 (0.22, 9.23)	0.84 (0.08, 9.17)	1.30 (0.18, 9.54)	**Pitavastatin**	1.10 (0.16, 7.46)	1.17 (0.16, 8.42)	1.20 (0.13, 10.66)
1.29 (0.45, 3.74)	0.77 (0.14, 4.09)	1.19 (0.42, 3.33)	0.91 (0.13, 6.20)	**Pravastatin**	1.07 (0.37, 3.06)	1.09 (0.27, 4.41)
1.21 (0.53, 2.77)	0.72 (0.16, 3.34)	1.11 (0.51, 2.46)	0.86 (0.12, 6.16)	0.94 (0.33, 2.70)	**Rosuvastatin**	1.02 (0.30, 3.51)
1.18 (0.33, 4.18)	0.70 (0.11, 4.42)	1.09 (0.30, 3.90)	0.83 (0.09, 7.42)	0.92 (0.23, 3.70)	0.98 (0.28, 3.34)	**Simvastatin**
**Liver Dysfunction**						
**Atorvastatin**	2.00 (0.91, 4.42)	0.78 (0.49, 1.24)	1.36 (0.67, 2.75)	1.41 (0.93, 2.15)	1.02 (0.59, 1.75)	1.09 (0.44, 2.72)
0.50 (0.23, 1.10)	**Fluvastatin**	0.39 (0.17, 0.90)	0.68 (0.24, 1.90)	0.71 (0.31, 1.60)	0.51 (0.21, 1.24)	0.54 (0.17, 1.76)
1.28 (0.80, 2.04)	2.57 (1.11, 5.93)	**Lovastatin**	1.74 (0.78, 3.90)	1.81 (1.08, 3.03)	1.30 (0.70, 2.43)	1.40 (0.52, 3.73)
0.74 (0.36, 1.49)	1.48 (0.53, 4.13)	0.57 (0.26, 1.29)	**Pitavastatin**	1.04 (0.51, 2.12)	0.75 (0.32, 1.76)	0.80 (0.26, 2.45)
0.71 (0.47, 1.08)	1.42 (0.62, 3.22)	0.55 (0.33, 0.92)	0.96 (0.47, 1.96)	**Pravastatin**	0.72 (0.40, 1.31)	0.77 (0.30, 2.01)
0.98 (0.57, 1.69)	1.97 (0.81, 4.81)	0.77 (0.41, 1.43)	1.34 (0.57, 3.15)	1.39 (0.76, 2.53)	**Rosuvastatin**	1.07 (0.39, 2.98)
0.92 (0.37, 2.29)	1.84 (0.57, 5.94)	0.72 (0.27, 1.91)	1.24 (0.41, 3.79)	1.30 (0.50, 3.38)	0.93 (0.34, 2.58)	**Simvastatin**

Renal Insufficiency				
**Atorvastatin**	2.29 (0.19, 28.04)	0.61 (0.18, 2.02)	1.09 (0.66, 1.78)	1.05 (0.31, 3.58)
0.44 (0.04, 5.34)	**Pitavastatin**	0.26 (0.02, 3.80)	0.47 (0.04, 5.69)	0.46 (0.04, 5.83)
1.65 (0.50, 5.50)	3.79 (0.26, 54.47)	**Pravastatin**	1.80 (0.55, 5.88)	1.73 (0.47, 6.42)
0.92 (0.56, 1.51)	2.11 (0.18, 25.28)	0.56 (0.17, 1.82)	**Rosuvastatin**	0.97 (0.29, 3.21)
0.95 (0.28, 3.25)	2.18 (0.17, 27.82)	0.58 (0.16, 2.14)	1.04 (0.31, 3.44)	**Simvastatin**

Diabetes				
**Atorvastatin**	1.16 (0.77, 1.73)	1.49 (1.08, 2.05)	1.17 (0.87, 1.57)	0.99 (0.76, 1.30)
0.86 (0.58, 1.29)	**Lovastatin**	1.28 (0.86, 1.91)	1.01 (0.70, 1.47)	0.86 (0.60, 1.22)
0.67 (0.49, 0.92)	0.78 (0.52, 1.16)	**Pitavastatin**	0.79 (0.59, 1.05)	0.67 (0.51, 0.87)
0.85 (0.64, 1.15)	0.99 (0.68, 1.44)	1.27 (0.95, 1.70)	**Pravastatin**	0.85 (0.68, 1.06)
1.01 (0.77, 1.31)	1.17 (0.82, 1.66)	1.50 (1.16, 1.94)	1.18 (0.95, 1.47)	**Rosuvastatin**

Eye Conditions			
**Atorvastatin**	1.30 (0.34, 4.92)	1.79 (0.50, 6.48)	1.27 (0.41, 3.95)
0.77 (0.20, 2.92)	**Lovastatin**	1.38 (0.53, 3.60)	0.98 (0.46, 2.06)
0.56 (0.15, 2.02)	0.72 (0.28, 1.89)	**Pravastatin**	0.71 (0.37, 1.37)
0.79 (0.25, 2.46)	1.02 (0.49, 2.16)	1.41 (0.73, 2.73)	**Rosuvastatin**

* Each cell is the odds ratio (95% confidence interval) of the treatment in the row compared to the treatment in the column for the risk of the outcome. Red cells are significantly higher risks in the comparisons.

Supplementary table 7. Q tests of global heterogeneity and inconsistency in network meta-analyses

Outcome	Total heterogeneity /inconsistency			Within-design heterogeneity			Between-design inconsistency (fixed-effect assumption)			Between-design inconsistency (random-effect assumption)			
	Q	Df	P	Q	Df	P	Q	Df	P	Q	Df	P	Tau^2
Muscle Symptoms	35.34	36	0.500	28.10	22	0.173	7.24	14	0.925	6.99	14	0.935	0.014
Muscle Disorders	13.27	39	0.999	9.11	26	0.999	4.16	13	0.989	4.16	13	0.989	0
Liver Dysfunction	22.22	35	0.954	10.34	21	0.974	11.88	14	0.616	11.88	14	0.616	0
Renal Insufficiency	7.47	18	0.986	4.68	8	0.791	2.79	10	0.986	2.79	10	0.986	0
Diabetes	5.52	5	0.355	5.51	4	0.239	0.02	1	0.898	0.02	1	0.901	0.009
Eye Conditions*	0.70	2	0.705	0.70	2	0.705	/	/	/	/	/	/	/

* For eye conditions, there was no indirect comparison and only one type of study design (direct comparison) was involved in each treatment comparison.

Supplementary table 8. Individual and comparative adverse effects of different statin types from random-effect network meta-analyses (sensitivity analyses)

**Muscle Symptoms**							
**Control**	0.94 (0.79, 1.12)	1.01 (0.85, 1.20)	0.94 (0.77, 1.14)	1.43 (0.89, 2.28)	1.01 (0.82, 1.25)	0.92 (0.86, 0.99)	1.56 (0.76, 3.22)
1.06 (0.89, 1.26)	**Atorvastatin**	1.07 (0.84, 1.37)	1.00 (0.77, 1.29)	1.51 (0.94, 2.45)	1.07 (0.82, 1.40)	0.98 (0.82, 1.17)	1.66 (0.81, 3.39)
0.99 (0.83, 1.17)	0.93 (0.73, 1.19)	**Fluvastatin**	0.93 (0.72, 1.20)	1.41 (0.85, 2.33)	1.00 (0.76, 1.31)	0.91 (0.76, 1.10)	1.55 (0.74, 3.25)
1.07 (0.88, 1.30)	1.00 (0.77, 1.30)	1.08 (0.83, 1.40)	**Lovastatin**	1.52 (0.91, 2.53)	1.08 (0.81, 1.44)	0.98 (0.80, 1.21)	1.67 (0.79, 3.52)
0.70 (0.44, 1.12)	0.66 (0.41, 1.07)	0.71 (0.43, 1.17)	0.66 (0.40, 1.10)	**Pitavastatin**	0.71 (0.45, 1.12)	0.65 (0.40, 1.04)	1.10 (0.51, 2.34)
0.99 (0.80, 1.22)	0.93 (0.72, 1.21)	1.00 (0.76, 1.31)	0.93 (0.70, 1.23)	1.41 (0.89, 2.23)	**Pravastatin**	0.91 (0.73, 1.13)	1.54 (0.74, 3.22)
1.08 (1.01, 1.16)	1.02 (0.85, 1.23)	1.10 (0.91, 1.32)	1.02 (0.83, 1.25)	1.55 (0.96, 2.49)	1.10 (0.88, 1.37)	**Rosuvastatin**	1.70 (0.82, 3.50)
0.64 (0.31, 1.32)	0.60 (0.30, 1.23)	0.65 (0.31, 1.36)	0.60 (0.28, 1.27)	0.91 (0.43, 1.95)	0.65 (0.31, 1.35)	0.59 (0.29, 1.22)	**Simvastatin**
**Muscle Disorders**							
**Control**	1.26 (0.55, 2.90)	0.74 (0.16, 3.31)	1.21 (0.75, 1.95)	0.93 (0.13, 6.76)	1.00 (0.38, 2.63)	1.06 (0.56, 2.03)	1.10 (0.33, 3.63)
0.79 (0.34, 1.83)	**Atorvastatin**	0.58 (0.11, 3.26)	0.96 (0.37, 2.49)	0.74 (0.11, 5.03)	0.79 (0.26, 2.42)	0.84 (0.36, 1.98)	0.87 (0.25, 3.12)
1.36 (0.30, 6.09)	1.71 (0.31, 9.52)	**Fluvastatin**	1.64 (0.34, 7.94)	1.27 (0.11, 15.19)	1.36 (0.23, 8.09)	1.44 (0.28, 7.40)	1.50 (0.22, 10.17)
0.83 (0.51, 1.33)	1.04 (0.40, 2.70)	0.61 (0.13, 2.94)	**Lovastatin**	0.77 (0.10, 5.89)	0.83 (0.28, 2.41)	0.88 (0.39, 1.96)	0.91 (0.25, 3.26)
1.07 (0.15, 7.76)	1.35 (0.20, 9.18)	0.79 (0.07, 9.48)	1.30 (0.17, 9.91)	**Pitavastatin**	1.07 (0.15, 7.54)	1.14 (0.15, 8.54)	1.18 (0.13, 10.82)
1.00 (0.38, 2.63)	1.26 (0.41, 3.85)	0.74 (0.12, 4.40)	1.21 (0.41, 3.54)	0.93 (0.13, 6.58)	**Pravastatin**	1.06 (0.35, 3.19)	1.10 (0.27, 4.54)
0.94 (0.49, 1.80)	1.19 (0.51, 2.78)	0.69 (0.14, 3.56)	1.14 (0.51, 2.54)	0.88 (0.12, 6.58)	0.94 (0.31, 2.82)	**Rosuvastatin**	1.04 (0.30, 3.55)
0.91 (0.28, 2.99)	1.14 (0.32, 4.08)	0.67 (0.10, 4.55)	1.10 (0.31, 3.93)	0.85 (0.09, 7.75)	0.91 (0.22, 3.73)	0.96 (0.28, 3.30)	**Simvastatin**
**Liver Dysfunction**							
**Control**	0.72 (0.55, 0.94)	1.42 (0.67, 2.98)	0.56 (0.38, 0.83)	0.94 (0.46, 1.95)	0.98 (0.69, 1.39)	0.72 (0.44, 1.18)	0.78 (0.31, 1.95)
1.39 (1.06, 1.82)	**Atorvastatin**	1.97 (0.89, 4.35)	0.79 (0.49, 1.26)	1.31 (0.64, 2.69)	1.36 (0.89, 2.09)	1 (0.58, 1.73)	1.09 (0.43, 2.73)
0.71 (0.34, 1.48)	0.51 (0.23, 1.12)	**Fluvastatin**	0.40 (0.17, 0.92)	0.67 (0.24, 1.88)	0.69 (0.30, 1.57)	0.51 (0.21, 1.24)	0.55 (0.17, 1.79)
1.77 (1.20, 2.61)	1.27 (0.80, 2.04)	2.51 (1.09, 5.81)	**Lovastatin**	1.67 (0.73, 3.80)	1.73 (1.03, 2.93)	1.27 (0.68, 2.39)	1.39 (0.52, 3.72)
1.06 (0.51, 2.19)	0.76 (0.37, 1.57)	1.50 (0.53, 4.25)	0.60 (0.26, 1.36)	**Pitavastatin**	1.04 (0.50, 2.15)	0.76 (0.32, 1.82)	0.83 (0.27, 2.55)
1.02 (0.72, 1.45)	0.73 (0.48, 1.12)	1.45 (0.64, 3.30)	0.58 (0.34, 0.97)	0.96 (0.47, 1.99)	**Pravastatin**	0.73 (0.40, 1.35)	0.80 (0.30, 2.10)
1.39 (0.85, 2.28)	1.00 (0.58, 1.72)	1.97 (0.81, 4.82)	0.78 (0.42, 1.47)	1.31 (0.55, 3.13)	1.36 (0.74, 2.49)	**Rosuvastatin**	1.09 (0.39, 3.03)
1.28 (0.51, 3.18)	0.92 (0.37, 2.30)	1.81 (0.56, 5.88)	0.72 (0.27, 1.94)	1.21 (0.39, 3.71)	1.25 (0.48, 3.28)	0.92 (0.33, 2.56)	**Simvastatin**

**Renal Insufficiency**					
**Control**	0.83 (0.51, 1.34)	1.86 (0.15, 22.49)	0.48 (0.13, 1.72)	0.88 (0.78, 1.00)	0.85 (0.24, 3.00)
1.21 (0.75, 1.97)	**Atorvastatin**	2.26 (0.18, 27.72)	0.58 (0.16, 2.09)	1.07 (0.65, 1.76)	1.03 (0.29, 3.69)
0.54 (0.04, 6.47)	0.44 (0.04, 5.43)	**Pitavastatin**	0.26 (0.02, 3.77)	0.47 (0.04, 5.71)	0.46 (0.04, 5.87)
2.09 (0.58, 7.53)	1.73 (0.48, 6.23)	3.90 (0.27, 57.31)	**Pravastatin**	1.85 (0.51, 6.66)	1.78 (0.48, 6.63)
1.13 (1.00, 1.28)	0.93 (0.57, 1.54)	2.11 (0.18, 25.44)	0.54 (0.15, 1.95)	**Rosuvastatin**	0.96 (0.27, 3.40)
1.18 (0.33, 4.15)	0.97 (0.27, 3.48)	2.19 (0.17, 28.23)	0.56 (0.15, 2.10)	1.04 (0.29, 3.67)	**Simvastatin**
**Diabetes**					
**Control**	0.88 (0.68, 1.14)	1.02 (0.72, 1.44)	1.31 (1.02, 1.68)	1.04 (0.85, 1.26)	0.88 (0.75, 1.02)
1.13 (0.88, 1.47)	**Atorvastatin**	1.16 (0.75, 1.78)	1.49 (1.04, 2.11)	1.18 (0.85, 1.63)	0.99 (0.74, 1.34)
0.98 (0.69, 1.39)	0.86 (0.56, 1.33)	**Lovastatin**	1.28 (0.84, 1.97)	1.02 (0.68, 1.52)	0.86 (0.59, 1.25)
0.76 (0.59, 0.98)	0.67 (0.47, 0.96)	0.78 (0.51, 1.20)	**Pitavastatin**	0.79 (0.58, 1.09)	0.67 (0.50, 0.90)
0.96 (0.79, 1.17)	0.85 (0.61, 1.17)	0.98 (0.66, 1.47)	1.26 (0.92, 1.74)	**Pravastatin**	0.84 (0.66, 1.08)
1.14 (0.98, 1.33)	1.01 (0.75, 1.36)	1.17 (0.80, 1.70)	1.50 (1.12, 2.01)	1.19 (0.92, 1.52)	**Rosuvastatin**
**Eye Conditions**					
**Control**	0.63 (0.20, 1.92)	0.82 (0.40, 1.69)	1.12 (0.60, 2.11)	0.79 (0.66, 0.96)	
1.60 (0.52, 4.89)	**Atorvastatin**	1.31 (0.35, 4.97)	1.79 (0.50, 6.48)	1.27 (0.41, 3.95)	
1.22 (0.59, 2.52)	0.76 (0.20, 2.90)	**Lovastatin**	1.37 (0.52, 3.58)	0.97 (0.46, 2.05)	
0.89 (0.47, 1.67)	0.56 (0.15, 2.02)	0.73 (0.28, 1.91)	**Pravastatin**	0.71 (0.37, 1.37)	
1.26 (1.04, 1.52)	0.79 (0.25, 2.46)	1.03 (0.49, 2.18)	1.41 (0.73, 2.73)	**Rosuvastatin**	

* Each cell is the odds ratio (95% confidence interval) of the treatment in the row compared to the treatment in the column for the risk of the outcome. Red cells are significantly higher risks in the comparisons.

Supplementary table 9. Node-splitting analyses of inconsistency between direct and indirect evidence in network meta-analyses

A Self-reported Muscle Symptoms

Treatment	Comparator	Direct	Indirect	NMA	Direct/Indirect	Z	P
Atorvastatin	Control	1.11 (0.91, 1.34)	0.89 (0.61, 1.30)	1.06 (0.90, 1.26)	1.243	1.003	0.316
Fluvastatin	Control	0.99 (0.83, 1.17)	\	0.99 (0.83, 1.17)	\	\	\
Lovastatin	Control	1.07 (0.88, 1.31)	\	1.07 (0.88, 1.31)	\	\	\
Pitavastatin	Control	\	0.70 (0.44, 1.12)	0.70 (0.44, 1.12)	\	\	\
Pravastatin	Control	1.02 (0.82, 1.26)	0.67 (0.31, 1.47)	0.99 (0.81, 1.22)	1.513	-1.005	0.315
Rosuvastatin	Control	1.08 (1.00, 1.16)	1.53 (0.99, 2.35)	1.09 (1.01, 1.16)	0.705	1.565	0.118
Simvastatin	Control	0.33 (0.01, 8.22)	0.67 (0.32, 1.40)	0.64 (0.31, 1.32)	0.489	0.423	0.672
Fluvastatin	Atorvastatin	\	0.93 (0.73, 1.19)	0.93 (0.73, 1.19)	\	\	\
Lovastatin	Atorvastatin	\	1.01 (0.78, 1.31)	1.01 (0.78, 1.31)	\	\	\
Pitavastatin	Atorvastatin	0.54 (0.23, 1.25)	0.74 (0.41, 1.31)	0.66 (0.41, 1.06)	0.730	0.604	0.546
Pravastatin	Atorvastatin	1.11 (0.30, 4.17)	0.93 (0.71, 1.21)	0.93 (0.72, 1.21)	1.199	-0.263	0.792
Rosuvastatin	Atorvastatin	1.37 (0.93, 2.02)	0.95 (0.78, 1.16)	1.02 (0.86, 1.22)	1.438	-1.627	0.104
Simvastatin	Atorvastatin	0.51 (0.21, 1.21)	1.00 (0.28, 3.58)	0.60 (0.30, 1.23)	0.506	0.866	0.386
Lovastatin	Fluvastatin	\	1.09 (0.84, 1.41)	1.09 (0.84, 1.41)	\	\	\
Pitavastatin	Fluvastatin	\	0.71 (0.43, 1.16)	0.71 (0.43, 1.16)	\	\	\
Pravastatin	Fluvastatin	\	1.00 (0.77, 1.31)	1.00 (0.77, 1.31)	\	\	\
Rosuvastatin	Fluvastatin	\	1.10 (0.91, 1.32)	1.10 (0.91, 1.32)	\	\	\
Simvastatin	Fluvastatin	\	0.65 (0.31, 1.36)	0.65 (0.31, 1.36)	\	\	\
Pitavastatin	Lovastatin	\	0.65 (0.39, 1.08)	0.65 (0.39, 1.08)	\	\	\
Pravastatin	Lovastatin	\	0.92 (0.69, 1.23)	0.92 (0.69, 1.23)	\	\	\
Rosuvastatin	Lovastatin	\	1.01 (0.82, 1.25)	1.01 (0.82, 1.25)	\	\	\
Simvastatin	Lovastatin	\	0.60 (0.28, 1.26)	0.60 (0.28, 1.26)	\	\	\
Pravastatin	Pitavastatin	1.15 (0.66, 2.02)	2.08 (0.97, 4.47)	1.41 (0.90, 2.22)	0.554	1.219	0.223
Rosuvastatin	Pitavastatin	1.01 (0.17, 5.96)	1.64 (1.01, 2.67)	1.55 (0.97, 2.48)	0.616	0.516	0.606
Simvastatin	Pitavastatin	1.42 (0.44, 4.56)	0.67 (0.25, 1.80)	0.91 (0.43, 1.95)	2.123	-0.962	0.336
Rosuvastatin	Pravastatin	1.43 (0.27, 7.67)	1.10 (0.88, 1.36)	1.10 (0.88, 1.36)	1.307	-0.311	0.756
Simvastatin	Pravastatin	0.24 (0.03, 2.20)	0.74 (0.34, 1.62)	0.65 (0.31, 1.35)	0.327	0.935	0.350
Simvastatin	Rosuvastatin	0.13 (0.01, 2.66)	0.64 (0.30, 1.35)	0.59 (0.29, 1.21)	0.208	0.998	0.318

B Clinically-confirmed Muscle Disorders

Treatment	Comparator	Direct	Indirect	NMA	Direct/Indirect	Z	P
Atorvastatin	Control	0.62 (0.21, 1.82)	0.99 (0.30, 3.20)	0.77 (0.35, 1.69)	0.625	-0.577	0.564
Fluvastatin	Control	1.29 (0.32, 5.24)	\	1.29 (0.32, 5.24)	\	\	\
Lovastatin	Control	0.84 (0.51, 1.36)	\	0.84 (0.52, 1.36)	\	\	\
Pitavastatin	Control	\	1.09 (0.16, 7.54)	1.09 (0.16, 7.54)	\	\	\
Pravastatin	Control	1.25 (0.42, 3.69)	0.55 (0.09, 3.31)	0.99 (0.40, 2.49)	2.275	-0.768	0.443
Rosuvastatin	Control	1.00 (0.50, 2.03)	0.74 (0.18, 3.07)	0.93 (0.50, 1.75)	1.362	-0.38	0.704
Simvastatin	Control	0.70 (0.11, 4.44)	1.14 (0.23, 5.79)	0.91 (0.28, 2.99)	0.609	0.394	0.693
Fluvastatin	Atorvastatin	\	1.68 (0.34, 8.38)	1.68 (0.34, 8.38)	\	\	\
Lovastatin	Atorvastatin	1.05 (0.02, 53.53)	1.10 (0.43, 2.82)	1.09 (0.43, 2.72)	0.958	0.021	0.984
Pitavastatin	Atorvastatin	2.00 (0.18, 22.15)	0.83 (0.04, 16.57)	1.42 (0.22, 9.23)	2.398	-0.447	0.655
Pravastatin	Atorvastatin	0.86 (0.15, 5.03)	1.80 (0.47, 6.87)	1.29 (0.45, 3.74)	0.476	0.656	0.512
Rosuvastatin	Atorvastatin	1.22 (0.40, 3.70)	1.36 (0.40, 4.67)	1.21 (0.53, 2.77)	0.892	0.135	0.892
Simvastatin	Atorvastatin	0.99 (0.22, 4.37)	0.81 (0.09, 7.71)	1.18 (0.33, 4.18)	1.217	-0.143	0.886
Lovastatin	Fluvastatin	\	0.65 (0.15, 2.84)	0.65 (0.15, 2.84)	\	\	\
Pitavastatin	Fluvastatin	\	0.84 (0.08, 9.17)	0.84 (0.08, 9.17)	\	\	\
Pravastatin	Fluvastatin	\	0.77 (0.14, 4.09)	0.77 (0.14, 4.09)	\	\	\
Rosuvastatin	Fluvastatin	\	0.72 (0.16, 3.34)	0.72 (0.16, 3.34)	\	\	\
Simvastatin	Fluvastatin	\	0.70 (0.11, 4.42)	0.70 (0.11, 4.42)	\	\	\
Pitavastatin	Lovastatin	\	1.30 (0.18, 9.54)	1.30 (0.18, 9.54)	\	\	\
Pravastatin	Lovastatin	0.99 (0.02, 50.38)	1.20 (0.41, 3.51)	1.19 (0.42, 3.33)	0.824	0.093	0.926
Rosuvastatin	Lovastatin	\	1.11 (0.51, 2.46)	1.11 (0.51, 2.46)	\	\	\
Simvastatin	Lovastatin	1.03 (0.02, 52.32)	1.08 (0.27, 4.25)	1.09 (0.30, 3.90)	0.950	0.024	0.981
Pravastatin	Pitavastatin	1.44 (0.09, 23.13)	0.60 (0.04, 8.50)	0.91 (0.13, 6.20)	2.398	-0.447	0.655
Rosuvastatin	Pitavastatin	\	0.86 (0.12, 6.16)	0.86 (0.12, 6.16)	\	\	\
Simvastatin	Pitavastatin	\	0.83 (0.09, 7.42)	0.83 (0.09, 7.42)	\	\	\
Rosuvastatin	Pravastatin	2.30 (0.09, 56.57)	0.86 (0.28, 2.64)	0.94 (0.33, 2.70)	2.666	-0.567	0.571
Simvastatin	Pravastatin	2.51 (0.25, 24.95)	0.34 (0.05, 2.47)	0.92 (0.23, 3.70)	7.357	-1.289	0.197
Simvastatin	Rosuvastatin	1.64 (0.22, 12.44)	0.51 (0.10, 2.60)	0.98 (0.28, 3.34)	3.217	-0.880	0.379

C Liver Dysfunction

Treatment	Comparator	Direct	Indirect	NMA	Direct/Indirect	Z	P
Atorvastatin	Control	1.30 (0.98, 1.72)	2.98 (1.30, 6.83)	1.41 (1.08, 1.85)	0.435	-1.860	0.063
Fluvastatin	Control	0.71 (0.34, 1.48)	\	0.71 (0.34, 1.48)	\	\	\
Lovastatin	Control	1.81 (1.24, 2.67)	\	1.81 (1.23, 2.66)	\	\	\
Pitavastatin	Control	\	1.04 (0.51, 2.12)	1.04 (0.51, 2.12)	\	\	\
Pravastatin	Control	1.08 (0.75, 1.55)	0.51 (0.17, 1.49)	1.00 (0.71, 1.41)	2.133	-1.302	0.193
Rosuvastatin	Control	1.49 (0.88, 2.54)	0.76 (0.20, 2.96)	1.39 (0.85, 2.28)	1.956	-0.902	0.367
Simvastatin	Control	1.74 (0.48, 6.28)	0.94 (0.25, 3.50)	1.30 (0.52, 3.21)	1.851	-0.656	0.512
Fluvastatin	Atorvastatin	\	0.50 (0.23, 1.10)	0.50 (0.23, 1.10)	\	\	\
Lovastatin	Atorvastatin	1.05 (0.02, 53.53)	1.29 (0.80, 2.06)	1.28 (0.80, 2.04)	0.815	0.101	0.919
Pitavastatin	Atorvastatin	0.70 (0.27, 1.82)	0.78 (0.27, 2.23)	0.74 (0.36, 1.49)	0.907	0.136	0.892
Pravastatin	Atorvastatin	0.20 (0.05, 0.88)	0.78 (0.51, 1.21)	0.71 (0.47, 1.08)	0.259	1.730	0.084
Rosuvastatin	Atorvastatin	0.62 (0.20, 1.89)	1.08 (0.59, 1.98)	0.98 (0.57, 1.69)	0.570	0.864	0.388
Simvastatin	Atorvastatin	0.40 (0.12, 1.40)	1.35 (0.36, 5.05)	0.92 (0.37, 2.29)	0.298	1.308	0.191
Lovastatin	Fluvastatin	\	2.57 (1.11, 5.93)	2.57 (1.11, 5.93)	\	\	\
Pitavastatin	Fluvastatin	\	1.48 (0.53, 4.13)	1.48 (0.53, 4.13)	\	\	\
Pravastatin	Fluvastatin	\	1.42 (0.62, 3.22)	1.42 (0.62, 3.22)	\	\	\
Rosuvastatin	Fluvastatin	\	1.97 (0.81, 4.81)	1.97 (0.81, 4.81)	\	\	\
Simvastatin	Fluvastatin	\	1.84 (0.57, 5.94)	1.84 (0.57, 5.94)	\	\	\
Pitavastatin	Lovastatin	\	0.57 (0.26, 1.29)	0.57 (0.26, 1.29)	\	\	\
Pravastatin	Lovastatin	0.99 (0.02, 50.38)	0.55 (0.33, 0.92)	0.55 (0.33, 0.92)	1.807	-0.292	0.770
Rosuvastatin	Lovastatin	\	0.77 (0.41, 1.43)	0.77 (0.41, 1.43)	\	\	\
Simvastatin	Lovastatin	1.03 (0.02, 52.32)	0.71 (0.25, 1.97)	0.72 (0.27, 1.91)	1.447	-0.178	0.859
Pravastatin	Pitavastatin	0.89 (0.33, 2.40)	1.04 (0.38, 2.89)	0.96 (0.47, 1.96)	0.854	0.218	0.827
Rosuvastatin	Pitavastatin	\	1.34 (0.57, 3.15)	1.34 (0.57, 3.15)	\	\	\
Simvastatin	Pitavastatin	1.95 (0.04, 99.09)	1.20 (0.37, 3.82)	1.24 (0.41, 3.79)	1.633	-0.235	0.814
Rosuvastatin	Pravastatin	0.77 (0.02, 38.64)	1.44 (0.79, 2.64)	1.39 (0.76, 2.53)	0.531	0.313	0.755
Simvastatin	Pravastatin	2.51 (0.25, 24.95)	1.20 (0.35, 4.12)	1.30 (0.50, 3.38)	2.082	-0.551	0.581
Simvastatin	Rosuvastatin	4.92 (0.24, 102.75)	0.84 (0.25, 2.84)	0.93 (0.34, 2.58)	5.839	-1.057	0.290

D Renal Insufficiency

Treatment	Comparator	Direct	Indirect	NMA	Direct/Indirect	Z	P
Atorvastatin	Control	1.30 (0.77, 2.19)	0.93 (0.24, 3.58)	1.23 (0.76, 1.99)	1.403	0.458	0.647
Pitavastatin	Control	\	0.54 (0.04, 6.44)	0.54 (0.04, 6.44)	\	\	\
Pravastatin	Control	5.23 (0.24, 112.06)	1.75 (0.39, 7.92)	2.03 (0.62, 6.66)	2.982	-0.627	0.531
Rosuvastatin	Control	1.13 (1.00, 1.28)	1.67 (0.46, 6.08)	1.13 (1.00, 1.28)	0.674	0.597	0.551
Simvastatin	Control	1.91 (0.21, 17.74)	1.00 (0.20, 5.07)	1.17 (0.35, 3.90)	1.909	-0.459	0.646
Pitavastatin	Atorvastatin	1.00 (0.02, 50.89)	0.48 (0.01, 29.90)	0.44 (0.04, 5.34)	2.067	-0.250	0.803
Pravastatin	Atorvastatin	1.65 (0.50, 5.50)	\	1.65 (0.50, 5.50)	\	\	\
Rosuvastatin	Atorvastatin	1.26 (0.39, 4.09)	0.86 (0.50, 1.48)	0.92 (0.56, 1.51)	1.460	-0.573	0.567
Simvastatin	Atorvastatin	1.41 (0.27, 7.25)	0.89 (0.06, 12.44)	0.95 (0.28, 3.25)	1.583	-0.290	0.772
Pravastatin	Pitavastatin	\	3.79 (0.26, 54.47)	3.79 (0.26, 54.47)	\	\	\
Rosuvastatin	Pitavastatin	3.06 (0.12, 76.02)	1.85 (0.03, 113.22)	2.11 (0.18, 25.28)	1.658	-0.190	0.850
Simvastatin	Pitavastatin	1.95 (0.04, 99.09)	2.37 (0.08, 66.88)	2.18 (0.17, 27.82)	0.826	0.073	0.942
Rosuvastatin	Pravastatin	0.87 (0.17, 4.50)	0.43 (0.07, 2.70)	0.56 (0.17, 1.82)	2.012	-0.556	0.578
Simvastatin	Pravastatin	0.58 (0.16, 2.14)	\	0.58 (0.16, 2.14)	\	\	\
Simvastatin	Rosuvastatin	0.66 (0.11, 3.96)	1.25 (0.23, 6.69)	1.04 (0.31, 3.44)	0.526	0.513	0.608

E Diabetes

Treatment	Comparator	Direct	Indirect	NMA	Direct/Indirect	Z	P
Atorvastatin	Control	1.14 (0.90, 1.44)	0.96 (0.25, 3.67)	1.14 (0.9, 1.43)	1.191	0.251	0.802
Lovastatin	Control	0.98 (0.71, 1.36)	\	0.98 (0.71, 1.36)	\	\	\
Pitavastatin	Control	0.76 (0.60, 0.95)	0.90 (0.24, 3.47)	0.76 (0.61, 0.96)	0.840	0.251	0.802
Pravastatin	Control	0.97 (0.81, 1.16)	\	0.97 (0.81, 1.16)	\	\	\
Rosuvastatin	Control	1.14 (1.00, 1.30)	\	1.14 (1.00, 1.30)	\	\	\
Lovastatin	Atorvastatin	\	0.86 (0.58, 1.29)	0.86 (0.58, 1.29)	\	\	\
Pitavastatin	Atorvastatin	0.79 (0.21, 2.98)	0.67 (0.48, 0.92)	0.67 (0.49, 0.92)	1.191	-0.251	0.802
Pravastatin	Atorvastatin	\	0.85 (0.64, 1.15)	0.85 (0.64, 1.15)	\	\	\
Rosuvastatin	Atorvastatin	\	1.01 (0.77, 1.31)	1.01 (0.77, 1.31)	\	\	\
Pitavastatin	Lovastatin	\	0.78 (0.52, 1.16)	0.78 (0.52, 1.16)	\	\	\
Pravastatin	Lovastatin	\	0.99 (0.68, 1.44)	0.99 (0.68, 1.44)	\	\	\
Rosuvastatin	Lovastatin	\	1.17 (0.82, 1.66)	1.17 (0.82, 1.66)	\	\	\
Pravastatin	Pitavastatin	\	1.27 (0.95, 1.70)	1.27 (0.95, 1.70)	\	\	\
Rosuvastatin	Pitavastatin	\	1.50 (1.16, 1.94)	1.50 (1.16, 1.94)	\	\	\
Rosuvastatin	Pravastatin	\	1.18 (0.95, 1.47)	1.18 (0.95, 1.47)	\	\	\

F Eye Conditions

Treatment	Comparator	Direct	Indirect	NMA	Direct/Indirect	Z	P
Atorvastatin	Control	1.60 (0.52, 4.89)	\	1.60 (0.52, 4.89)	\	\	\
Lovastatin	Control	1.23 (0.60, 2.53)	\	1.23 (0.60, 2.53)	\	\	\
Pravastatin	Control	0.89 (0.47, 1.67)	\	0.89 (0.47, 1.67)	\	\	\
Rosuvastatin	Control	1.26 (1.04, 1.52)	\	1.26 (1.04, 1.52)	\	\	\
Lovastatin	Atorvastatin	\	0.77 (0.20, 2.92)	0.77 (0.20, 2.92)	\	\	\
Pravastatin	Atorvastatin	\	0.56 (0.15, 2.02)	0.56 (0.15, 2.02)	\	\	\
Rosuvastatin	Atorvastatin	\	0.79 (0.25, 2.46)	0.79 (0.25, 2.46)	\	\	\
Pravastatin	Lovastatin	\	0.72 (0.28, 1.89)	0.72 (0.28, 1.89)	\	\	\
Rosuvastatin	Lovastatin	\	1.02 (0.49, 2.16)	1.02 (0.49, 2.16)	\	\	\
Rosuvastatin	Pravastatin	\	1.41 (0.73, 2.73)	1.41 (0.73, 2.73)	\	\	\

Supplementary figure 6. E_{max} dose-response curves with dose-specific adverse effects of individual statins

A Self-reported Muscle Symptoms

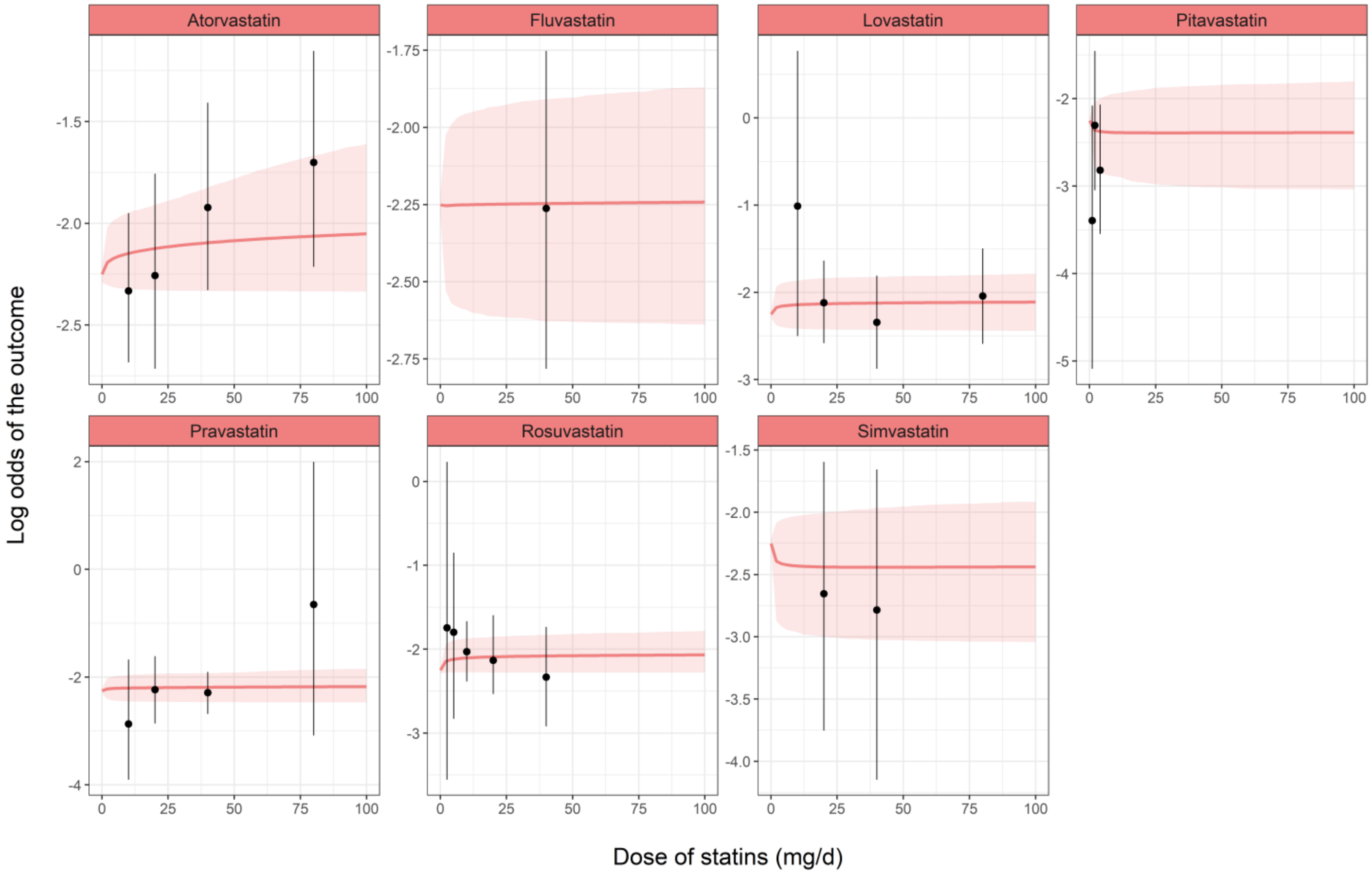

B Clinically-confirmed Muscle Disorders

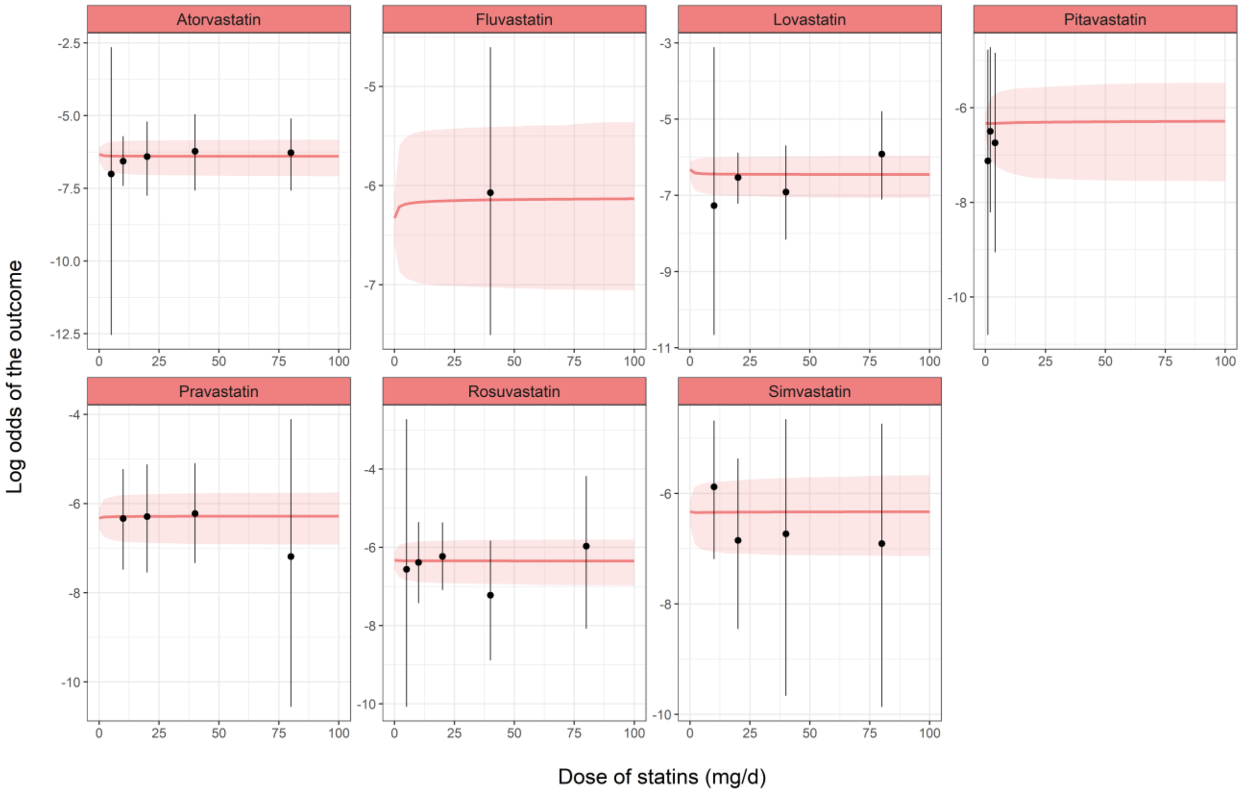

C Liver Dysfunction

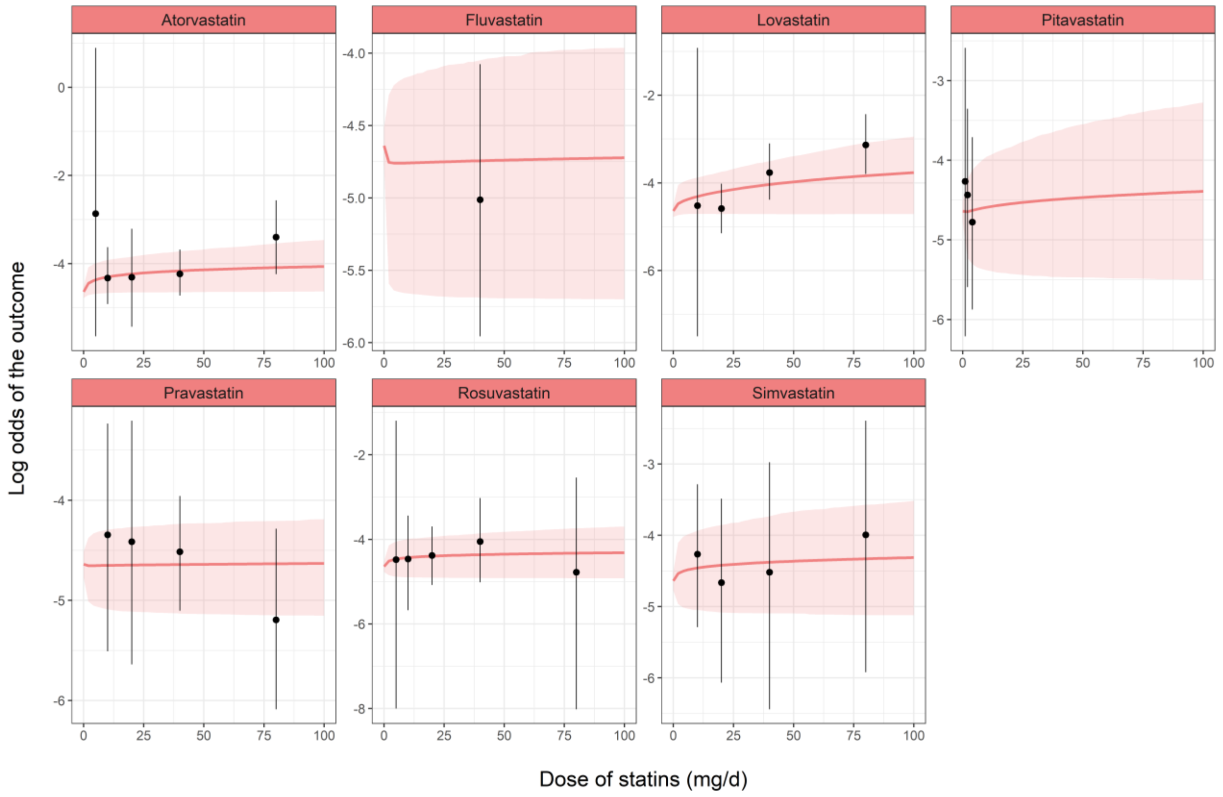

D Renal Insufficiency

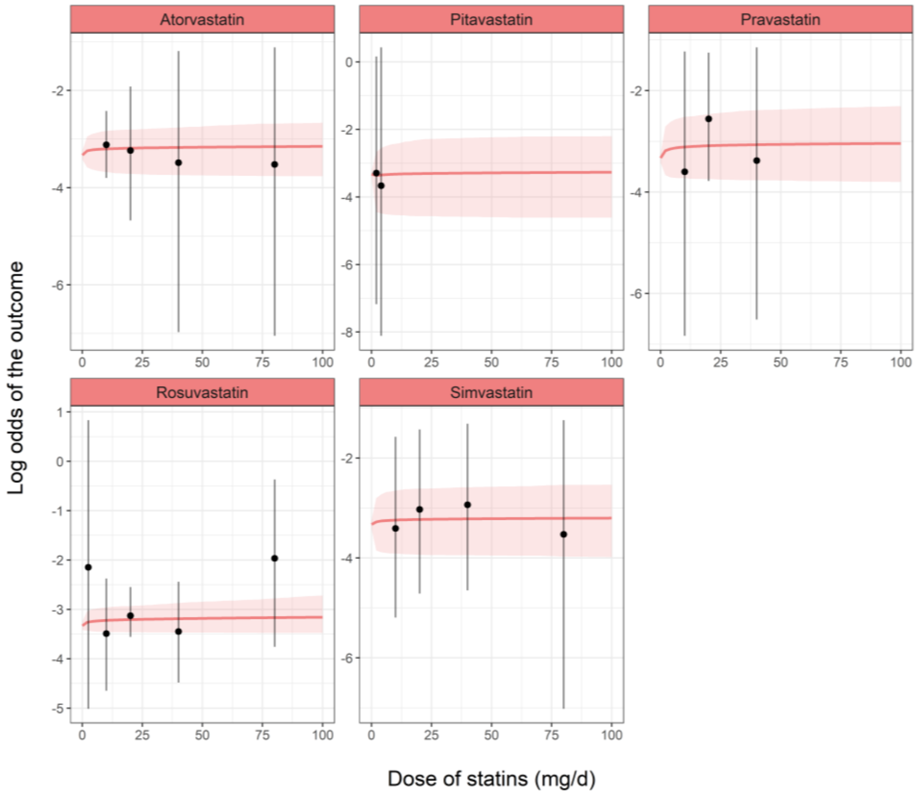

E Diabetes

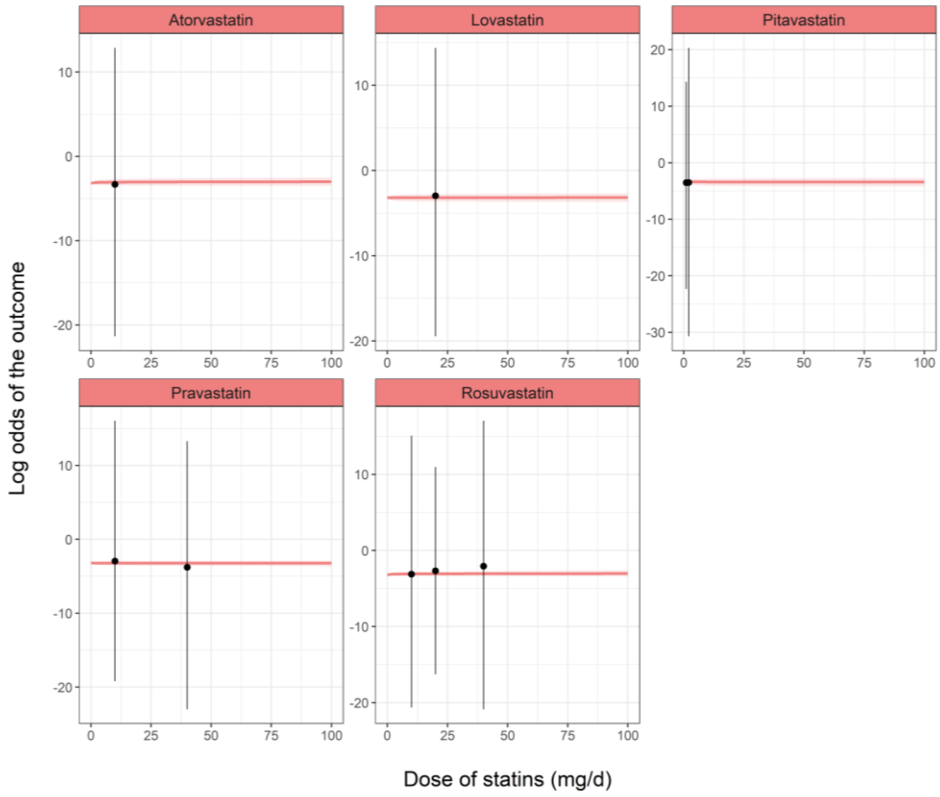

F Eye Conditions

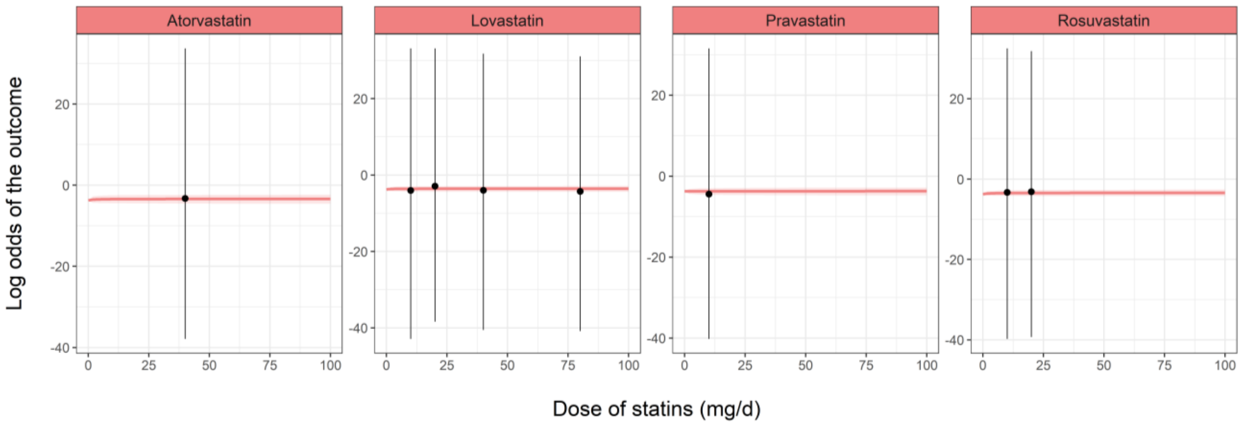

Supplement: Supplementary file 1 — Web appendix: Supplementary material [file cait064948.ww.pdf]
